# Efficacy and Safety of Anti-HER2 Agents in Combination With Chemotherapy for Metastatic HER2-Positive Breast Cancer Patient: A Network Meta-Analysis

**DOI:** 10.3389/fonc.2021.731210

**Published:** 2021-08-19

**Authors:** Xiaohui Zhang, Junsheng Leng, Yidong Zhou, Feng Mao, Yan Lin, Songjie Shen, Qiang Sun

**Affiliations:** ^1^Department of Breast Surgery, Peking Union Medical College Hospital, Peking Union Medical College & Chinese Academy of Medical Sciences (CAMS), Beijing, China; ^2^Department of Orthopedics, Peking Union Medical College Hospital, Peking Union Medical College & Chinese Academy of Medical Sciences (CAMS), Beijing, China

**Keywords:** efficacy, safety, anti-HER2 agents, metastatic HER2-positive breast cancer, network meta-analysis

## Abstract

**Background:**

The presence of anti-HER2 agents, such as trastuzumab, pertuzumab, and trastuzumab emtansine (T-DM1), significantly improved the prognosis of metastatic HER2-positive (HER2+) breast cancers (BC). However, drug resistance and disease progression are still common. In order to further improve the treatment efficacy, new clinical trials about anti-HER2 agents in combination with chemotherapy are growing rapidly. We conducted the network meta-analysis to synthesize evidences of clinical trials to identify the best therapy for metastatic HER2+ BC.

**Methods:**

A systematic search of randomized controlled trials regarding anti-HER2 agents in combination with chemotherapy for advanced or metastatic breast cancers up to May 2020 was conducted in Embase, PubMed, and the Cochrane Library. The primary outcome was progression-free survival (PFS). The secondary outcomes were overall survival (OS), objective response rate (ORR), and safety. Bayesian network meta-analysis was conducted to synthesize the results and rank the therapies.

**Results:**

Twenty-six studies, including 16 studies for first-line treatments and 10 studies for second- or later-line treatments were included in the network meta-analysis. For first-line studies, the THP (taxanes + trastuzumab + pertuzumab) regimen exhibited the highest probability to be the optimal treatment in all efficacy outcomes and moderate safety. For second- or later-line studies, the T-DM1 and XHTuC (capecitabine + trastuzumab + tucatinib) regimens ranked top two in all efficacy outcomes according to the surface under the cumulative ranking (SUCRA) results. T-DM1 ranked first in PFS and OS whereas XHTuC ranked first in ORR. The safety outcomes of T-DM1 and XHTuC were acceptable.

**Conclusions:**

THP was still the optimal first-line treatment for metastatic HER2+ BC. T-DM1 and XHTuC were recommended for second-line treatments.

**Systematic Review Registration:**

INPLASY.com, identifier (INPLASY202090086).

## Introduction

The human epidermal growth factor receptor 2 (HER2) is a tyrosine kinase cell membrane receptor and an important biomarker of breast cancer (BC) molecular subtypes. HER2-positive (HER2+) BC accounts for 15%–20% of total BC and exhibits aggressive behavior and poor prognosis (1). HER2 testing is performed *via* immunohistochemistry (IHC) and fluorescence *in situ* hybridization (FISH) (2). The status of HER2 expression predicts response to anti-HER2 agents.

In 1998, the first HER2-targeted agent Trastuzumab was approved by the US Food and Drug Administration (FDA) for the treatment of metastatic HER2+ BC (3). Trastuzumab is a monoclonal antibody of HER2. Adding trastuzumab to chemotherapy significantly improved time to disease progression, objective response rate (ORR), and survival time for patients with metastatic HER2+ BC (4).

Despite the efficacy of trastuzumab in inhibiting HER2-overexpressing tumor cells, drug resistance is common and the disease eventually progresses in most metastatic HER2+ BC patients. Therefore, other anti-HER2 agents have been evolving to further improve the prognosis of these patients and some agents exhibited good efficacy. For example, pertuzumab, another HER2-targeted agent, enhanced progression-free survival (PFS) and overall survival (OS) in metastatic HER2+ BC patients when in combination with trastuzumab and docetaxel in CLEOPATRA (5), and trastuzumab emtansine (T-DM1), an antibody-drug conjugate of HER2, significantly improved OS in previously treated advanced BC patients in EMILIA (6). Now, there are seven FDA-approved anti-HER2 agents, including trastuzumab, pertuzumab, T-DM1, lapatinib, tucatinib, neratinib, and trastuzumab deruxtecan (1). As for the chemotherapy part, there are many other options than taxanes, such as capecitabine, vinorelbine (7), and doxorubicin (8), while the optimal combination of anti-HER2 agents and chemotherapeutic drugs for metastatic HER2+ BC was not clear.

The new clinical trials about anti-HER2 agents in combination with chemotherapy are growing rapidly and the new evidence might challenge the previous treatment standard. However, it is difficult to determine the best regimen according to these evidences because most of the direct comparisons are not included in these trials.

Network meta-analysis, also known as multiple treatment meta-analysis, is an extension of pairwise meta-analysis. It allows comparisons of multiple interventions with the evidence of all the relevant RCTs (9). The Bayesian approach is a statistical model of network meta-analysis that produces a posterior probability distribution by a prior probability distribution using Bayes theorem and allows the output of ranking and probability results of an overall estimate (10). Therefore, the Bayesian network meta-analysis is suitable for comparisons of various treatments of metastatic HER2-positive BC in multiple trials. In this study, we conducted the Bayesian network meta-analysis to synthesize evidences of previous clinical trials to evaluate the efficacy and safety of each therapy and identify the best therapy for metastatic HER2+ BC.

## Methods

### Study Design

Bayesian network meta-analysis was carried out in this study. The network meta-analysis was conducted and reported according to the preferred reporting items for systematic review and meta-analysis (PRISMA) extension statement (11). The protocol was registered on INPLASY.com (INPLASY202090086).

### Data Sources

A systematic search of published literatures and registered trials was conducted in the following databases on May 30, 2020: Embase (from 1974 to May 2020), PubMed (from 1966 to May 2020), and the Cochrane Central Register of Controlled Trials (CENTRAL) (the Cochrane Library, May 2020). Search strategies for all databases are listed in **Supplementary Data 1**.

### Eligibility Criteria

#### Type of Patients

We included patients (>18 years old) with metastatic or advanced HER2+ BC, defined as histologically or cytologically confirmed BC patients of IHC score of 3 positive (3+) or 2+ and/or HER2 gene amplification by FISH. The stage of the patients was proven to be metastatic or advanced. There was no limitation on sex, comorbidity, and hormone receptor status.

#### Type of Designs

We included relevant randomized controlled trials (RCTs). Systematic reviews or meta-analyses were also included to track their references.

#### Type of Interventions

Anti-HER2 agents in combination with chemotherapy for advanced or metastatic BC were included. The anti-HER2 agents include trastuzumab, pertuzumab, trastuzumab emtansine (T-DM1/TDM1), lapatinib, pyrotinib, afatinib, neratinib, margetuximab, tucatinib, and trastuzumab deruxtecan (DS-8201). We included single use of anti-HER2 agent or a combination of any two types of anti-HER2 agents. Concurrent or sequential chemotherapy were also included. There was no limitation on dosage, frequency, time, method of administration, treatment duration, and combined drug for chemotherapy.

We defined taxanes as docetaxel and/or paclitaxel.

#### Type of Outcomes

The primary outcome was PFS: the time from randomization to death or any disease progression event. TTP (time to progression) was also used as PFS for studies that did not report on PFS.

The secondary outcomes included:

- OS: the time from randomization until death, using an intention to treat analysis;
- ORR: the percentage of patients who had a complete response or partial response;
- Safety: risks of serious adverse events (SAEs), including leucopenia, neutropenia, febrile neutropenia, and cardiac adverse events. Leucopenia, neutropenia, and febrile neutropenia were defined as Grade 3 or 4 adverse events according to Common Terminology Criteria for Adverse Events version 5.0. Specifically, leucopenia was defined as absolute leukocyte count <2000/mm^3^, neutropenia was defined as absolute neutrophil count <1000/mm^3^, and febrile neutropenia was defined as absolute neutrophil count <1000/mm^3^ with a single temperature of >38.3°C or a sustained temperature of ≥38°C for more than 1 h. Cardiac adverse events were defined as cardiac failure or left ventricular ejection fraction (LVEF) <50% or >14% reduction of LVEF after therapy.

There was no limitation on year of publication, publication status, and duration of study follow-up or period of study conduct.

### Exclusion Criteria

- We excluded non-RCTs, observational studies, and single-arm studies.
- We excluded studies including participants with early-stage BC, HER2-negative BC, or a mixed HER2 status without subgroup data in the HER2+ population, a mixed population with all lines of treatment and studies investigating adjuvant/neoadjuvant therapies.
- We excluded papers that were not reported in English or Chinese.
- We excluded reports without full reports and where only abstract was available.
- If multiple publications were reported for the same trial or included the same or overlapping patient groups, only publications with the most recent interested data or with the largest sample size were included.
- We excluded studies with interventions that were only comprised in one study.
- We excluded studies that compared the same drugs between different manufacturers.

### Study Selection

Two authors independently performed study screening. Titles and abstracts of searched results were firstly screened. Full texts of studies that passed title/abstract screening were perused in detail to confirm eligibility. We also checked trial registration status and result reports for trials that passed title/abstract screening to confirm eligibility. Any disagreement between two authors was resolved by discussion. If there was no consensus, a third reviewer was consulted.

### Data Extraction and Quality Assessment

For each study, the following information were extracted by two authors independently: the first author’s name, the published year, source of funding, country, setting, inclusion criteria, exclusion criteria, diagnostic criteria, sample sizes, age of patients, hormone receptor status, line of treatment, disease status, baseline performance status, HER2 status, metastatic sites, previous treatment, time of follow-up, comparison and treatment details. PFS, TTP, and OS were reported as hazard ratios (HRs) with 95% credible intervals (CrIs) and ORR was reported as odds ratios (ORs) with 95% CrIs. Safety (adverse events) was reported as incidence rate.

Two authors independently assessed the risk of bias in the included studies with the help of measures displayed in Cochrane Handbook V.5.1.0 for Systematic Reviews of Interventions (12). The tool included seven domains, which were random sequence generation, allocation concealment, blinding of participants and personnel, blinding of outcome assessment, incomplete outcome data, selective reporting, and other bias (if there was commercial funding, study early discontinuation, or baseline imbalance, we judged the domain as high risk of bias). The judgment for each domain was low risk of bias, high risk of bias, or moderate risk of bias. Any disagreement was coordinated by the third author to reach consensus.

### Statistical Analysis

#### Standard Pairwise Meta-Analysis

Where possible, standard pairwise meta-analysis was performed by R software. Pooled ORs/HRs with 95% CrI were calculated for outcomes. The random effect model was used to perform meta-analysis (12). Heterogeneity of treatment effects across trials were assessed by *p*-value and *I* ^2^ statistics. If the *p*-value <0.1 and *I* ^2^ is >50%, we explored sources of heterogeneity; subgroup analysis and meta-regression were conducted when the factors inducing heterogeneity were identified. Where the factors inducing heterogeneity were not identified, we pooled the data in random effect model; however, our confidence on the study findings was compromised. Funnel plot would be used to test potential publication bias (13). For comparisons with a single study, meta-analysis is not applicable; we presented results from original studies directly.

#### Bayesian Network Meta-Analysis

A Bayesian network meta-analysis was performed by R software. The random effect models with vague priors for multi-arm trials developed by Lu and Ade were used (14). The pooled estimation and the probability of which drug is the best was obtained by the Markov Chains Monte Carlo method. Three Markov Chains were run simultaneously with different arbitrarily chosen initial values. The model convergence was assessed by trace plots and Brooks–Gelman–Rubin plots (15). The results of dichotomous outcomes were reported as posterior medians of OR/HR CrIs. Evidence inconsistency and clinical similarity in patient characteristics and settings across trials were carefully assessed. Network geometry was performed by R software. The plot function graphically showed the direct comparison between the treatment groups comprising the network. The thickness of the edge for connecting nodes means the amount of data. The fixed effect model was created for inconsistency test by entering the network setup data in the “mtc.nodesplit” function. The treatment ranking was shown from the top priority to the lowest priority treatments. The surface under the cumulative ranking area (SUCRA) will be calculated to summarize and report the probability values. SUCRA values will be expressed as percentages; SUCRA value will be 100% for the best treatment, while SUCRA value will be 0% for the worst treatment.

### Subgroup Analysis

We conducted subgroup analysis for the primary outcome (PFS) where data were available according to hormone receptor status of participants: hormone receptor positive (HR+) defined as estrogen receptor positive (ER+) and/or progesterone receptor positive (PR+), and hormone receptor negative (HR−) defined as estrogen receptor negative (ER−) and progesterone receptor negative (PR−).

## Results

### Study Selection

**Figure 1:**
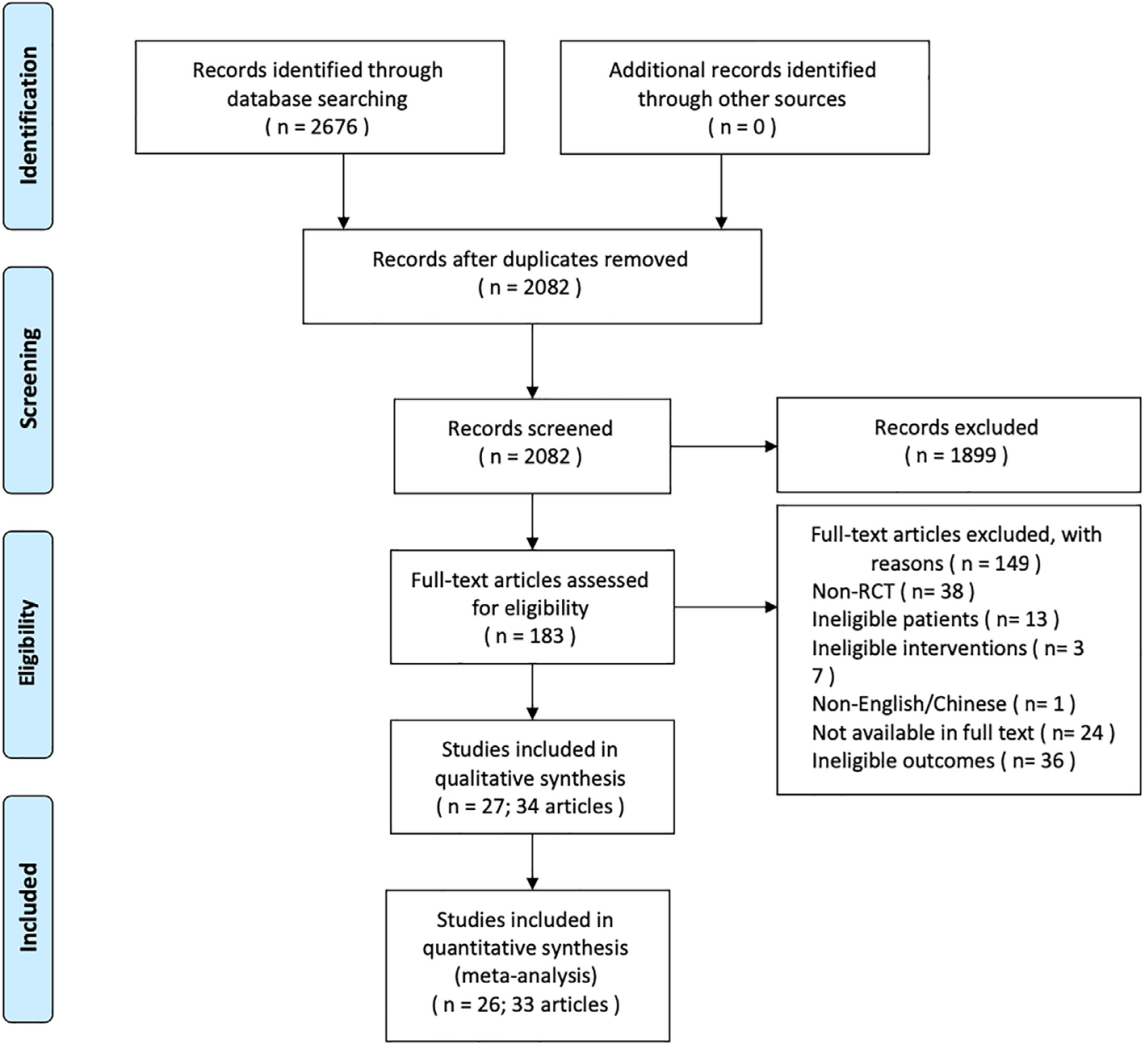
PRISMA flow diagram.

The PRISMA flow diagram is shown in **Figure 1**. The search identified 2,676 related references. After removal of 594 duplicate references, 2,082 records were screened. Thirty-four publications were eligible for inclusion criteria, whereas others were not selected for various reasons (e.g., non-random design, ineligible patients, or interventions). Twenty-seven studies with 34 reports (5–8, 16–45) were included in the systematic reviews, while 26 studies with 33 reports were included for meta-analysis as Tolaney (2020) (45) was not able to provide data for meta-analysis. One hundred forty-nine studies were excluded. A list of excluded records during full-text screening could be found in **Supplementary Data 2**.

### Characteristics of Included Studies

Twenty-seven studies with 34 reports (5–8, 16–45) were included in the systematic reviews, while 26 studies with 33 reports were included for meta-analysis as Tolaney (2020) (45) was not able to provide data for meta-analysis. **Table 1** provides a summary of the included studies. A total of 9,792 participants were included in this meta-analysis. The study sample size ranged from 22 to 1,095. These studies were published between 2005 and 2020. All studies were multi-center and most studies were multi-region.

**Table 1:** Study and patient characteristics.

Study ID	Country/region	*N* of participants	*N* of males	Age (median (range)/mean (SD), years)	Baseline performance status (ECOG PS)	HER2 status (definitions)	Median follow-up time (months)
First Line							
Andersson (23)	Denmark, Sweden, and Norway	284	NR	56 (29–72)	TH/VH: 0–94/96, 1–338/36, 2–9/9	IHC 3+ or FISH+	34
Awada (36)	Australia, Bahamas, Belarus, Belgium, Bulgaria, Canada, China, Croatia, Denmark, France, Germany, Hong Kong, Hungary, India, Israel, Italy, Japan, Korea, Republic of, Latvia, Lithuania, Malaysia, Malta, Poland, Portugal, Romania, Serbia, Singapore, South Africa, Spain, Switzerland, Taiwan, Turkey, Ukraine, UK, USA	479	0	54.1 (11.3)	TN/TH: 0–150/152, 1–86/79, 2–6/5, unknown–0/1	IHC 3+ or IHC 2+ and FISH+/CISH+	23.0
Baselga (28)/Swain (5)/2020 (44)	Argentina, Brazil, Canada, China, Costa Rica, Croatia, Ecuador, Finland, France, Germany, Guatemala, Italy, Japan, Korea, Latvia, Macedonia, Philippines, Poland, Russia, Singapore, Spain, Thailand, UK, USA	808	2	TH/THP: 54 (27–89)/54 (22–82)	TH/THP: 0–248/274, 1–157/125, >=2–1/3	IHC 3+ or FISH+	TH/THP: 98.7/99.9
Baselga (8)	Argentina, Canada, Germany, India, Italy, Poland, Portugal, Russian Federation, Spain, UK, USA	363	0	TAH/TH: 52 (22–79)/53 (30–76)	TAH/TH: 0–113/112, 1–68/70	NR	44
Burstein (19)	USA	85	NR	VH/TH: 55 (36–79)/50 (37–83)	TH/VH: 0–27/22, 1–11/16, 2–3/2	IHC 3+ and/or FISH+	NR
Gasparini (20)	Multi-region	124	0	T/TH: 54 (30–71)/56 (32–72)	TH/T: 0–51/49, 1–8/8, 2–4/3	HercepTest assay (score 2+ or 3+)	16.6
Gianni (30)	Argentina, Australia, Austria, Bosnia and Herzegovina, Brazil, Canada, Czech Republic, France, Italy, Mexico, Romania, Russian Federation, Spain, Turkey, UK, Uruguay	424	0	TBevH/TH: 53 (26–82)/55 (22–83)	0–1	IHC3+ or FISH/CISH+	26
Guan (31)	Russia, Pakistan, Peru, China [mainland and Hong Kong], Thailand, Brazil, and Ukraine	444	5	TL/T: 50.0 (25–74)/50.5 (26–73)	TL/T: 0–103/113, 1–1193/109	FISH+	TL/T: 25.7/23.6
Hamberg (24)	Netherlands	101	0	TH/H-T:50 (32–74)/54 (36–74)	TH/H-T: 0–26/23, 1–26/22, 2–1/1	IHC3+ and/or FISH+	NR
Hurvitz, (32)	Multi-region	137	0	TH/T-DM1: 52 (33–75)/55 (27–82)	TH/T-DM1: 0–45/44, 1–25/23	IHC 3+ or FISH+	14
Hurvitz (35)	Argentina, Australia, Belgium, Brazil, Canada, China, Colombia, Egypt, France, Germany, Greece, Hong Kong, Ireland, Italy, Japan, Korea, Republic of, Lebanon, Mexico, Peru, Puerto Rico, Russian Federation, South Africa, Switzerland, Taiwan, Turkey, UK, USA, Venezuela	719	0	TEveH/TH: 54.0 (23–86)/52.0 (19–82)	TEveH/TH: 0–278/148, 1–202/91	NR	41.3
Marty (16)	Multi-region (11 European countries and Australia)	188	0	TH/T: 53(32–80)/55(24–79)	TH/T: 0 (0–4)/0 (0–2)	IHC 3+ and/or FISH+ (originally defined as IHC 2+ and 3+ but revised later)	TH/T: 40.9/35.9
Perez (38)/2019 (41)	Argentina, Australia, Austria, Bahamas, Belgium, Bosnia and Herzegovina, Brazil, Canada, Colombia, Czechia, Denmark, France, Germany, Greece, Guatemala, Hungary, Italy, Japan, Korea, Republic of, Macedonia, The Former Yugoslav Republic of, Malaysia, Mexico, New Zealand, Panama, Peru, Philippines, Poland, Portugal, Romania, Russian Federation, Spain, Sweden, Switzerland, Taiwan, Thailand, Turkey, UK, USA	1095	0	TH/T-DM1/TdmP: 55 (22–88)/52 (27–82)/52 (27–86)	TH/T-DM1/TdmP: 0–245/239/235, 1–119/128/127	IHC 3+ and/or FISH+	35
Robert (18)	USA, Canada	196	0	TCbH/TH: 55 (35–81)/56 (33–83)	TCbH/TH: 0–5/60, 1–35/35, 2–4/3	IHC 3+ or 2+ (revised to IHC3+ or IHC 2+ and FISH+ after enrollment of 60 participants)	NR
Valero (26)	USA, Australia, Russia, Poland, Germany, France, Spain, Ireland, Canada, Croatia, Belgium, Switzerland	263	0	51	Median Karnofsky performance status TH/TCbH: 90/100	FISH+	NR
Wardley (22)	Multi-region (43 centers)	225	0	TXH/TH: 53 (24–82)/52 (23–78)	0 (0–2)	IHC 3+ or FISH+	24
Second or Other Line							
Geyer (17)	UK, France, Poland, Australia, Israel	324	0	XL/X: 54 (26–80)/51 (28–83)	XL/X: 0–96/89, 1–61/68, unknown-6/4	IHC3+ or IHC2+ and FISH+	NR
Gómez (7)	Argentina, Brazil, Peru	142	0	51 (20–84)	XL/VL/GemL: 0/1–49/44/45, 2–2/1/1	IHC 3+ or FISH+	21
Krop (34)/2017 (37)	USA, Korea, Spain, France, Switzerland, Belgium	602	NR	TPC/T-DM1: 54 (28–85)/53 (27–89)	TPC/T-DM1: 0–82/180, 1–101/200, 2–15/22	IHC 3+ or FISH+	30.5
Lin (25)	USA, Canada, the European Union, and Israel	22	0	XL/LTop: 49 (38–63)/55 (37–69)	XL/LTop: 0–7/1, 1–6/8	IHC 3+ or FISH+	XL/LTop: 16/10
Martin (33)	USA, Western Europe, Australia, South Africa and Canada, Asia-Pacific, India, Eastern Europe, Africa and South America	233	0	N/XL: 52 (28–79)/56 (30–79)	N/XL: 0–70/69, 1–43/39, 2–3/25	IHC 3+ or IHC 2+ and FISH+/CISH+	NR
Murthy (43)/Lin (42)	USA, Canada, Australia, Austria, Belgium, Switzerland, Czech Republic, Germany, Denmark, Spain, France, Great Britain, Israel, Italy and Portugal	612	5	54	XHTuc/XH: 0–204/94, 1–206/108	IHC confirmed or FISH+	14
Takano (40)	Japan	86	0	XH/XL: 57 (34–81)/59 (37–78)	XH/HL: 0–23/31, 1–1/12, 2–2/0	IHC 3+ or FISH+	44.6
Urruticoechea (39)	Asia, Europe, North America, South America	452	0	XH/XHP: 55/54	XH/XHP: 0–145/158, 1–73/68, 2–2/1	IHC 3+ and/or FISH+	XH/XHP: 28.6/25.3
Verma (29)/Diéras (6)	UK, Canada, USA, Germany, Switzerland, Italy, Bosnia and Herzegovina, Brazil, Bulgaria, Denmark, Finland, France, Hong Kong, Korea, Mexico, New Zealand, Poland, Portugal, Russia, Singapore, Slovenia, Sweden, Spain, Taiwan	991	5	XL/T-DM1: 53 (24–83)/53 (25–84)	XL/T-DM1: 0–312/299, 1–176/194, NA–8/2	IHC 3+ and/or FISH+	XL/T-DM1: 41.9/47.8
von Minckwitz (21)/2011 (27)	Germany, Austria, the Netherlands, Slovenia, Denmark, UK	156	0	X/XH: 59 (33–82)/52.5 (28–78)	NR	IHC 3+ or FISH+	20.7
Third or Other Line							
Tolaney (45)	Argentina, Australia, Belgium, Brazil, Canada, France, Germany, Greece, Italy, Mexico, Spain, South Korea, UK, USA	237	0	FHAbe/Habe/CT+H: 55 (47–62)/54(47–62)/57 (47–67)	NR	NR	19

ECOG PS, Eastern Cooperative Oncology Group Performance Status; SD, standard deviation; TH, Taxanes + Trastuzumab; TXH, Capecitabine + Taxanes + Trastuzumab; TCbH, Carboplatin + Taxanes + Trastuzumab; T-DM1, Trastuzumab emtansine; TEveH, Everolimus + Taxanes + Trastuzumab; H-T, Sequential Trastuzumab → Docetaxel; TL, Lapatinib + Taxanes; TBevH, Bevacizumab + Taxanes + Trastuzumab; T, Taxanes, TAH, NPLD + Taxanes + Trastuzumab; THP, Pertuzumab + Taxanes + Trastuzumab; TN, Neratinib + Taxanes; VH, Trastuzumab + Vinorelbine

#### Patient Characteristics

Among enrolled studies, four RCTs included a total of 17 males. Mean age ranged from 50 to 59. Sixteen studies (5, 8, 16, 18–20, 22–24, 26, 28, 30–32, 35, 36, 38, 41, 44) included patients for first-line treatment, 10 studies (6, 7, 17, 21, 25, 27, 29, 33, 34, 37, 39, 40, 42, 43) for second or later lines and one study for third or later lines. Two studies (21, 27, 45) did not report on baseline performance status of the participants in Eastern Cooperative Oncology Group Performance Status (ECOG PS). Twenty-three studies (5–7, 16–19, 21, 22, 24–34, 36–44) defined HER2 status by IHC and/or FISH (**Table 1**).

#### Intervention Characteristics

Among first-line studies, 15 used trastuzumab and taxanes combination as one of the study interventions, three studies used taxanes, two studies used trastuzumab and vinorelbine combination, two studies used T-DM1, and another two studies used carboplatin, trastuzumab, and taxanes combination. One study compared three interventions and the rest compared two interventions.

Among second- or later-line studies, six used capecitabin and lapatinib combination as one of the study interventions, four studies used capecitabine and trastuzumab combination, two studies used T-DM1, two studies used capecitabine monotherapy, and one study compared abemaciclib and trastuzumab combination, with or without fulvestrant and also compared to standard of care (SOC) chemotherapy with trastuzumab. Two studies compared three interventions and the rest compared two interventions.

Detailed intervention characteristics can be found in **Supplementary Table 1**.

#### Outcome Characteristics

Among first-line studies, all 16 reported ORR and safety outcomes; 12 studies (5, 8, 18, 22, 24, 26, 28, 30–32, 35, 36, 38, 41, 44) reported PFS outcomes, and another 4 studies (16, 19, 20, 23) reported TTP instead of PFS, of which 1 (20) study’s HR result was extracted from figures, while another 2 studies (16, 19) did not report HR results and were not included in the meta-analysis; 13 studies (5, 8, 16, 18, 23, 24, 26, 28, 30–32, 35, 36, 38, 41, 44) also reported OS outcomes, of which 1 (35) study’s HR result was extracted from figures. Nine studies (5, 8, 20, 22, 23, 28, 30, 35, 36, 38, 41, 44) had hormone receptor subgroup analysis for PFS, of which one (20) study reported OR result that should be HR result according to the methodology description; another study (22) did not report 95% CI and was not included in the meta-analysis.

Among second- or other-line studies, nine reported (6, 7, 17, 21, 27, 29, 33, 34, 37, 39, 40, 42, 43) PFS, OS, ORR, and safety outcomes, of which one study (7) did not report HR results for PFS or OS outcome and was not included in the meta-analysis for PFS or OS outcome, and another study (21) reported TTP instead of PFS and was used as PFS for analysis, while another study (25) did not report survival outcomes, but reported safety outcomes and CNS ORR [defined as a ≥50% volumetric reduction of CNS lesion(s) in the absence of new or progressive CNS or non-CNS lesions, or increasing steroid requirements], which was not used for ORR analysis. Five studies (6, 29, 34, 37, 39, 40, 42, 43) had hormone receptor subgroup analysis for PFS.

One study (45) in third or other line reported PFS, ORR, and safety outcome and had hormone receptor subgroup analysis for PFS.

Summary of outcomes reported in each study can be found in **Supplementary Table 1**.

### Risk of Bias in Included Studies

Eight studies had moderate/low risk of bias in all seven domains (**Supplementary Table 2**). The major sources of high risk of bias derived from blinding of participants and investigators (*N* = 16), blinding of outcome assessment (*N* = 4), other (*N* = 4), allocation concealment (*N* = 2), and study attrition (*N* = 1).

#### Randomization

Seven studies did not describe randomization method in detail and were rated as moderate risk of bias in this domain. Other studies were rated as low risk of bias. No high risk of bias was assessed in this domain.

#### Allocation Concealment

Twelve studies did not report on allocation concealment and were rated as moderate risk of bias in this domain. Two open-label studies were rated as high risk of bias. Other studies were rated as low risk of bias in this domain.

#### Blinding of Participants and Investigators

Five studies did not report on blinding of participants and investigators and were rated as moderate risk of bias in this domain. Sixteen studies were open-label or did not mask for participants and investigators and were rated as high risk of bias. Other studies were rated as low risk of bias in this domain.

#### Blinding of Outcome Assessment

Five studies did not report on blinding of outcome assessment and were rated as moderate risk of bias in this domain. Four studies did not mask for investigators and were rated as high risk of bias. Other studies were rated as low risk of bias in this domain.

#### Selective Report of Outcomes

Two studies were rated as moderate risk of bias for a deduction in enrollment and might cause bias in outcomes. Other studies were rated low risk of bias for this domain.

#### Study Attrition

One study did not report on attrition and was rated as moderate risk of bias, and another study was rated as high risk of bias for a loss of follow-up of more than 20% of enrollment. Other studies were rated low risk of bias in this domain.

#### Other

Eighteen studies were rated as moderate risk of bias for a possible bias-causing funding, and four studies were rated as high risk for financial support or an early closing of the trial. Other studies were rated low risk of bias in this domain.

### Primary Outcomes

#### First-Line Treatment

**Figure 2:**
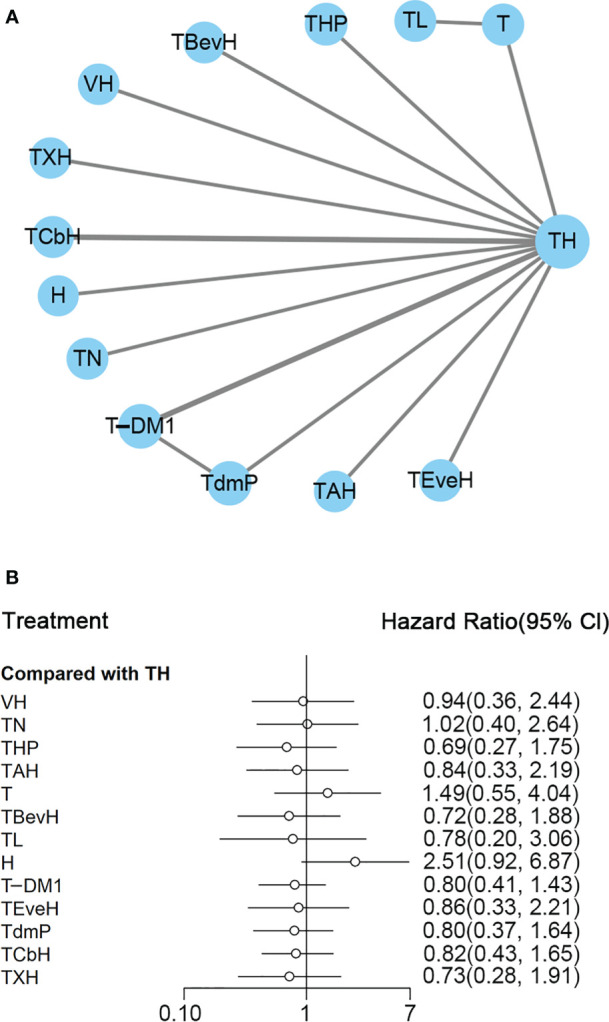
**(A)** Network plot of PFS in first-line studies. **(B)** Forest plot of PFS in first-line studies. Notes: The size of the blue node is proportional to the total number of participants assigned to each intervention. The width of the line is proportional to the number of trials comparing each pair of treatment. The thickness of the edge for connecting nodes means the amount of data. TH, Taxanes + Trastuzumab; VH, Trastuzumab + Vinorelbine; TXH, Capecitabine + Taxanes + Trastuzumab; TCbH, Carboplatin + Taxanes + Trastuzumab; TdmP, Pertuzumab + trastuzumab emtansine; TEveH, Everolimus + Taxanes + Trastuzumab; T-DM1, trastuzumab emtansine; H, Trastuzumab; TL, Lapatinib + Taxanes; TBevH, Bevacizumab + Taxanes + Trastuzumab; T, Taxanes; TAH, NPLD + Taxanes + Trastuzumab; THP, Pertuzumab + Taxanes + Trastuzumab; TN, Neratinib + Taxanes.

A total of 14 studies with 5,662 patients were included, and network plot is shown in **Figure 2A**. One study (24) reported PFS result, and data from PFScomb *versus* PFStras were used in the meta-analysis.

In the network meta-analysis, there was no significant difference between TH (Taxanes + Trastuzumab) and other interventions. Details of network meta-analyses are indicated in **Figure 2B** and **Supplementary Data 3**. Network meta-analyses showed no difference between interventions. The results of direct comparisons are presented in **Supplementary Data 3**. Result showed that THP (Pertuzumab + Taxanes + Trastuzumab, HR 0.69, 95% CI 0.59–0.81), TBevH (Bevacizumab + Taxanes + Trastuzumab, HR 0.72, 95% CI 0.54–0.94), TCbH (Carboplatin + Taxanes + Trastuzumab, HR 0.71, 95% CI 0.64–0.79), and TXH (Capecitabine + Taxanes + Trastuzumab, HR 0.725, 95% CI 0.53–0.99) had better PFS than TH (Taxanes + Trastuzumab); however, H (Trastuzumab) had even worse PFS than TH (HR 2.51, 95% CI 1.61–3.91). TL (Lapatinib + Taxanes) had better PFS than T (Taxanes, HR 0.52, 95% CI 0.42–0.64). There was no significant difference between other intervention groups.

**Figure 3:**
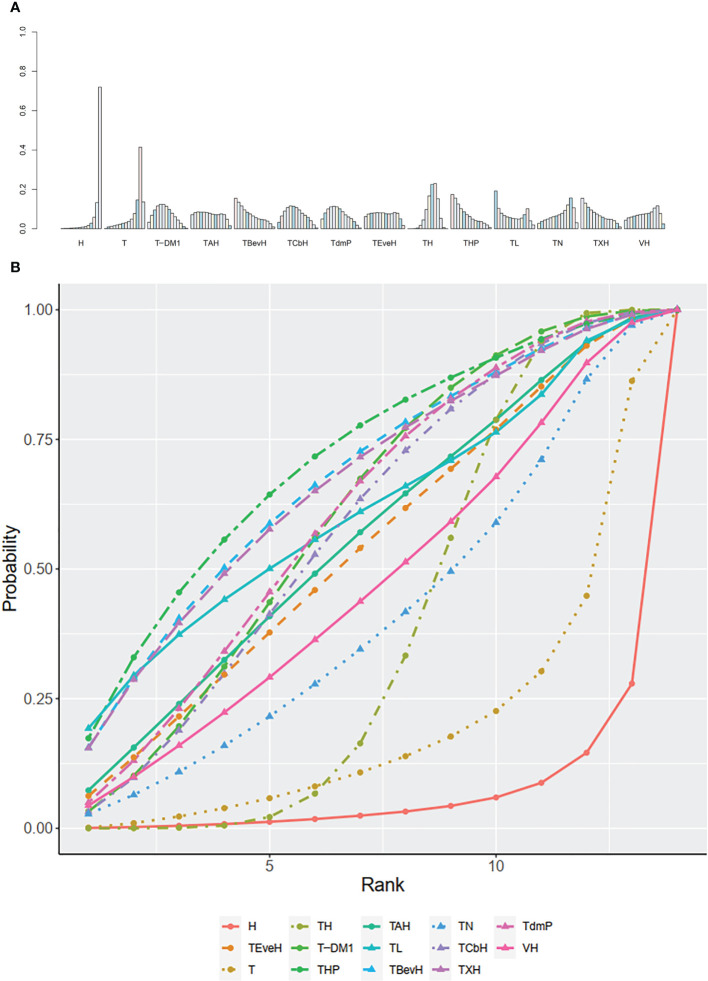
**(A)** Ranking histogram for PFS in first-line studies. **(B)** Result of SUCRA for PFS in first-line studies.

The ranking histogram showed that TL (Lapatinib + Taxanes, 19.2% probability), THP (Pertuzumab + Taxanes + Trastuzumab, 17.4% probability), TBevH (Bevacizumab + Taxanes + Trastuzumab, 15.5% probability), and TXH (Capecitabine + Taxanes + Trastuzumab, 15.5% probability) ranked first; H (Trastuzumab, 72.0% probability) ranked last (**Figure 3A**). The result of SUCRA showed that THP (Pertuzumab + Taxanes + Trastuzumab, 70.5% probability) ranked first; TBevH (Bevacizumab + Taxanes + Trastuzumab, 67.1% probability) ranked second; TXH (Capecitabine + Taxanes + Trastuzumab, 66.5% probability) ranked third; H (Trastuzumab, 5.5% probability) ranked last (**Figure 3B**).

The trace plot and density plot showed a good convergence degree (**Supplementary Data 4A**).

Except for those interventions where the loop is not able to be constructed, the inconsistency test result can be found in **Supplementary Data 4B**, which showed no significant inconsistency between direct and indirect results.

##### HR+ Subgroup Analysis

A total of eight studies with 2,229 patients were included.

In network meta-analysis, there was no significant difference between TH (Taxanes + Trastuzumab) and other interventions, and details of network meta-analyses were indicated in **Supplementary Data 5A–C**. Network meta-analyses showed no difference between interventions. The results of direct comparisons were presented in **Supplementary Data 5C**. Results showed that THP (Pertuzumab + Taxanes + Trastuzumab) had better PFS than TH (Taxanes + Trastuzumab, HR 0.72, 95% CI 0.55–0.59). There was no significant difference between other intervention groups.

The ranking histogram showed that VH (Trastuzumab + Vinorelbine, 27.7% probability), THP (Pertuzumab + Taxanes + Trastuzumab, 24.0% probability), and TdmP (Pertuzumab + trastuzumab emtansine, 23.7% probability) ranked first; TBevH (Bevacizumab + Taxanes + Trastuzumab, 16.7% probability) ranked third; T (Taxanes, 58.6% probability) ranked last (**Supplementary Data 5D-1**). The result of SUCRA showed that THP (Pertuzumab + Taxanes + Trastuzumab, 75.1% probability) ranked first; VH (Trastuzumab + Vinorelbine, 74.9% probability) ranked second; TdmP (Pertuzumab + trastuzumab emtansine, 68.6% probability) ranked third; T (Taxanes, 16.0% probability) ranked last (**Supplementary Data 5D-2**).

The trace plot and density plot showed a good convergence degree (**Supplementary Data 5E**).

The inconsistency test is not available as the no-intervention loop was constructed.

##### HR− Subgroup Analysis

A total of eight studies with 2,017 patients were included.

In the network meta-analysis, there was no significant difference between TH (Taxanes + Trastuzumab) and other interventions, and details of network meta-analyses are indicated in **Supplementary Data 6A–C**. Network meta-analyses showed no difference between interventions. The results of direct comparisons were presented in **Supplementary Data 6C**. Results showed that THP (Pertuzumab + Taxanes + Trastuzumab, HR 0.55, 95% CI 0.42–0.72) and TEveH (Everolimus + Taxanes + Trastuzumab, HR 0.61, 95% CI 0.42–0.87) had better PFS than TH (Taxanes + Trastuzumab). There was no significant difference between other intervention groups.

The ranking histogram showed that THP (Pertuzumab + Taxanes + Trastuzumab, 42.2% probability) and TEveH (Everolimus + Taxanes + Trastuzumab, 27.8% probability) ranked first, and TAH (NPLD + Taxanes + Trastuzumab, 21.2% probability) ranked third; T (Taxanes, 35.6% probability) and VH (Trastuzumab + Vinorelbine, 33.6% probability) ranked last (**Supplementary Data 6D-1**). The result of SUCRA showed that THP (Pertuzumab + Taxanes + Trastuzumab, 84.5% probability) ranked first; TEveH (Everolimus + Taxanes + Trastuzumab, 78.3% probability) ranked second; TAH (NPLD + Taxanes + Trastuzumab, 65.7% probability) ranked third; and VH (Trastuzumab + Vinorelbine, 21.4% probability) ranked last (**Supplementary Data 6D-2**).

The trace plot and density plot showed a good convergence degree (**Supplementary Data 6E**).

The inconsistency test is not available as the no-intervention loop was constructed.

#### Second- or Other-Line Treatment

**Figure 4:**
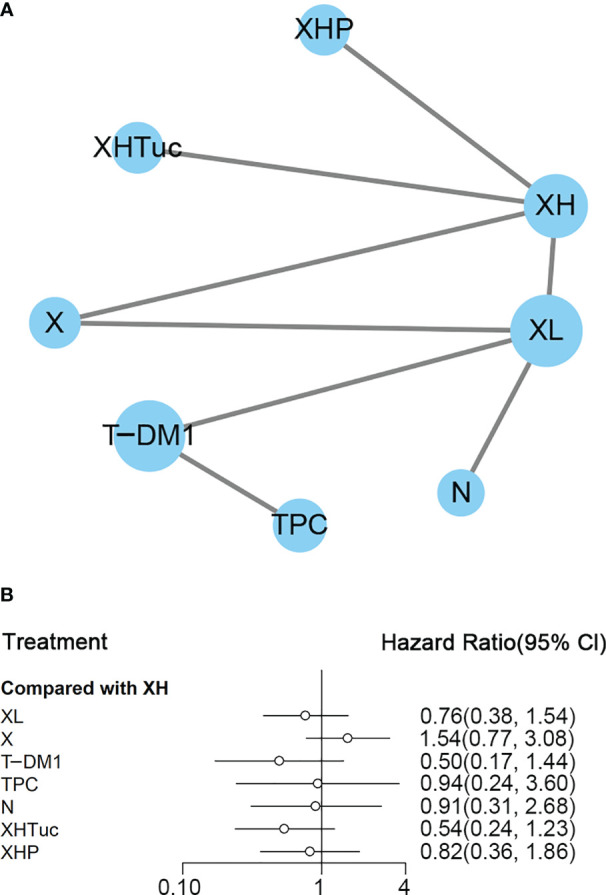
**(A)** Network plot of PFS in second- or other-line studies. **(B)** Forest plot of PFS in second- or other-line studies Notes: The size of blue node is proportional to the total number of participants assigned to each intervention. The width of the line is proportional to the number of trials comparing each pair of treatment. The thickness of the edge for connecting nodes means the amount of data. XH, Capecitabine + Trastuzumab; XHTuc, Capecitabine + Trastuzumab + Tucatinib; N, Neratinib; TPC, Physician’s choice; T-DM1, trastuzumab emtansine; X, Capecitabine; XL, Capecitabine + Lapatinib; XHP, Capecitabine + Pertuzumab + Trastuzumab.

A total of eight studies with 3,324 patients were included. PFS data from 480 patients firstly randomized in the studies of Murthy (2020) (43)/Lin (2020) (42) were used in this network analysis. The network plot is shown in **Figure 4A**. Murthy (43)/Lin (42) (XHTuc *vs*. XH) reported PFS outcomes for both the first 480 patients and brain metastatic patients, respectively; we used the outcome of the first 480 patients in this meta-analysis

In the network meta-analysis, there was no significant difference between XH (Capecitabine + Trastuzumab) and other interventions, and details of network meta-analyses are indicated in **Figure 4B** and **Supplementary Data 7**. Network meta-analysis showed a beneficial effect on PFS in XL (Capecitabine + Lapatinib) compared with X (Capecitabine, HR 0.50, 95% CI 0.25–0.99) (**Supplementary Data 7**); however, there was no difference between other interventions (**Supplementary Data 7**). The result of direct comparisons showed a beneficial effect on PFS in XH (Capecitabine + Trastuzumab, HR 0.69, 95% CI 0.48–0.97) and XL (Capecitabine + Lapatinib, HR 0.47, 95% CI 0.33–0.67) groups compared with the X (Capecitabine) group; XHTuc (Capecitabine + Trastuzumab + Tucatinib) had better PFS than XH (Capecitabine + Trastuzumab, HR 0.54, 95% CI 0.42–0.71). However, T-DM1 had better PFS than TPC (Physician’s choice, HR 0.528, 95% CI 0.422–0.661) and XL (Capecitabine + Lapatinib, HR 0.65, 95% CI 0.55–0.77) (**Supplementary Data 7**). There were no differences found between the other groups.

**Figure 5:**
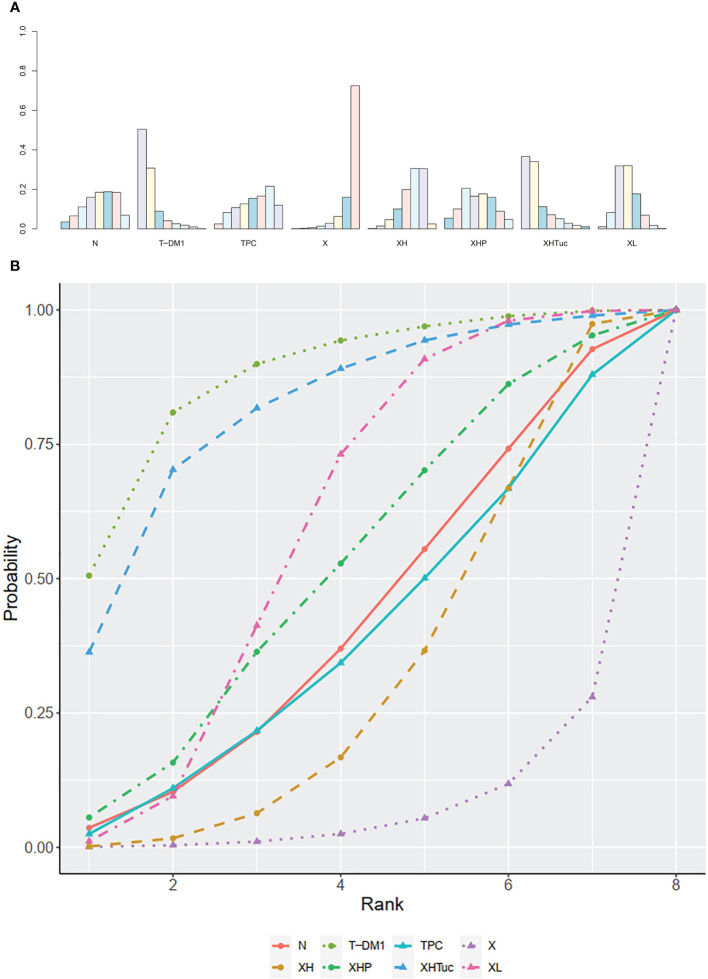
**(A)** Ranking histogram for PFS in second- or other-line studies. **(B)** Result of SUCRA for PFS in second- or other-line studies.

The ranking histogram showed that T-DM1 (trastuzumab emtansine, 50.4% probability) and XHTuc (Capecitabine + Trastuzumab + Tucatinib, 36.6% probability) ranked first, and XL (Capecitabine + Lapatinib, 32.0% probability) ranked third; X (Capecitabine, 72.5% probability) ranked last (**Figure 5A**). The result of SUCRA showed that T-DM1 (trastuzumab emtansine, 87.3% probability) ranked first, XHTuc (Capecitabine + Trastuzumab + Tucatinib, 81.2% probability) ranked second, XL (Capecitabine + Lapatinib, 59.1% probability) ranked third, and X (Capecitabine, 7.0% probability) ranked last (**Figure 5B**).

The trace plot and density plot showed a good convergence degree (**Supplementary Data 8A**). Except for those interventions where the loop is not able to be constructed, the inconsistency test result can be found in **Supplementary Data 8B**, which showed no significant inconsistency between direct and indirect results.

##### HR+ Subgroup Analysis

A total of five studies with 1,448 patients were included. PFS data from 480 patients firstly randomized in the studies of Murthy (2020) (43)/Lin (2020) (42) were used in this network analysis.

In the network meta-analysis, there was no significant difference between XH (Capecitabine + Trastuzumab) and other interventions, and details of network meta-analyses are indicated in **Supplementary Data 9A–C**. Network meta-analyses showed no difference between interventions. The result of direct comparisons in **Supplementary Data 9C** showed a beneficial effect on PFS in T-DM1 (trastuzumab emtansine) compared to TPC (Physician’s choice, HR 0.56, 95% CI 0.41–0.76) and XL (Capecitabine + Lapatinib, HR 0.72, 95% CI 0.58–0.91); XHTuc (Capecitabine + Trastuzumab + Tucatinib) had better PFS than XH (Capecitabine + Trastuzumab, HR 0.58, 95% CI 0.42–0.8). There were no differences found between the other groups.

The ranking histogram showed that T-DM1 (trastuzumab emtansine, 43.4% probability) and XHTuc (Capecitabine + Trastuzumab + Tucatinib, 43.1% probability) ranked first and XL (Capecitabine + Lapatinib, 31.5% probability) ranked third; TPC (Physician’s choice, 42.9% probability) ranked last (**Supplementary Data 9D-1**). The result of SUCRA showed that T-DM1 (trastuzumab emtansine, 79.0% probability) ranked first, XHTuc (Capecitabine + Trastuzumab + Tucatinib, 76.1% probability) ranked second, XL (Capecitabine + Lapatinib, 49.0% probability) ranked third, and XH (Capecitabine + Trastuzumab, 27.3% probability) ranked last (**Supplementary Data 9D-2**).

The trace plot and density plot showed a good convergence degree (**Supplementary Data 9E**).

The inconsistency test is not available as the no-intervention loop was constructed.

##### HR− Subgroup Analysis

A total of five studies with 1,130 patients were included. PFS data from 480 patients firstly randomized in the studies of Murthy (2020) (43)/Lin (2020) (42) were used in this network analysis.

In network meta-analysis, there was no significant difference between XH (Capecitabine + Trastuzumab) and other interventions, and details of network meta-analyses were indicated in **Supplementary Data 10A–C**. Network meta-analyses showed no difference between interventions. The result of direct comparisons in **Supplementary Data 10C** showed a beneficial effect on PFS in T-DM1 (trastuzumab emtansine) compared to TPC (Physician’s choice, HR 0.51, 95% CI 0.37–0.71) and XL (Capecitabine + Lapatinib, HR 0.56, 95% CI 0.44–0.72); XHTuc (Capecitabine + Trastuzumab + Tucatinib) had better PFS than XH (Capecitabine + Trastuzumab, HR 0.54, 95% CI 0.34–0.60); and XHP (Capecitabine + Pertuzumab + Trastuzumab) had better PFS than XH (Capecitabine + Trastuzumab, HR 0.72, 95% CI 0.51–1.00). There were no differences found between the other groups.

The ranking histogram showed that T-DM1 (trastuzumab emtansine, 43.8% probability) and XHTuc (Capecitabine + Trastuzumab + Tucatinib, 38.5% probability) ranked first and XHP (Capecitabine + Pertuzumab + Trastuzumab, 23.4% probability) ranked second; XH (Capecitabine + Trastuzumab, 30.7% probability) and TPC (Physician’s choice, 39.5% probability) ranked last (**Supplementary Data 10D-1**). The result of SUCRA showed that T-DM1 (trastuzumab emtansine, 78.9% probability) ranked first, XHTuc (Capecitabine + Trastuzumab + Tucatinib, 73.5% probability) ranked second, XHP (Capecitabine + Pertuzumab + Trastuzumab, 55.0% probability) ranked third, and XH (Capecitabine + Trastuzumab, 26.8% probability) ranked last (**Supplementary Data 10D-2**).

The trace plot and density plot showed a good convergence degree (**Supplementary Data 10E**).

The inconsistency test is not available as the no-intervention loop was constructed.

#### Third- or Other-Line Treatment

Tolaney (45) reported investigator-assessed PFS of abemaciclib plus trastuzumab plus fulvestrant *versus* trastuzumab plus SOC chemotherapy (HR 0.673, 95% CI 0.451–1.003, *n* = 158) and also PFS of abemaciclib plus trastuzumab *versus* trastuzumab plus SOC chemotherapy (HR 0.943, 95% CI 0.643–1.383, *n* = 158).

In the progesterone receptor-positive subgroup, PFS of abemaciclib plus trastuzumab plus fulvestrant *versus* trastuzumab plus SOC chemotherapy was HR 0.726, 95% CI 0.458–1.152, *n* = 106; PFS of abemaciclib plus trastuzumab *versus* trastuzumab plus SOC chemotherapy was HR 1.098, 95% CI 0.695–1.733, *n* = 102.

In the progesterone receptor-negative subgroup, PFS of abemaciclib plus trastuzumab plus fulvestrant *versus* trastuzumab plus SOC chemotherapy was HR 0.858, 95% CI 0.413–1.779, *n* = 47; PFS of abemaciclib plus trastuzumab *versus* trastuzumab plus SOC chemotherapy was HR 0.989, 95% CI 0.497–1.969, *n* = 51.

### Secondary Outcomes

#### Overall Survival

##### First-Line Treatment

**Figure 6:**
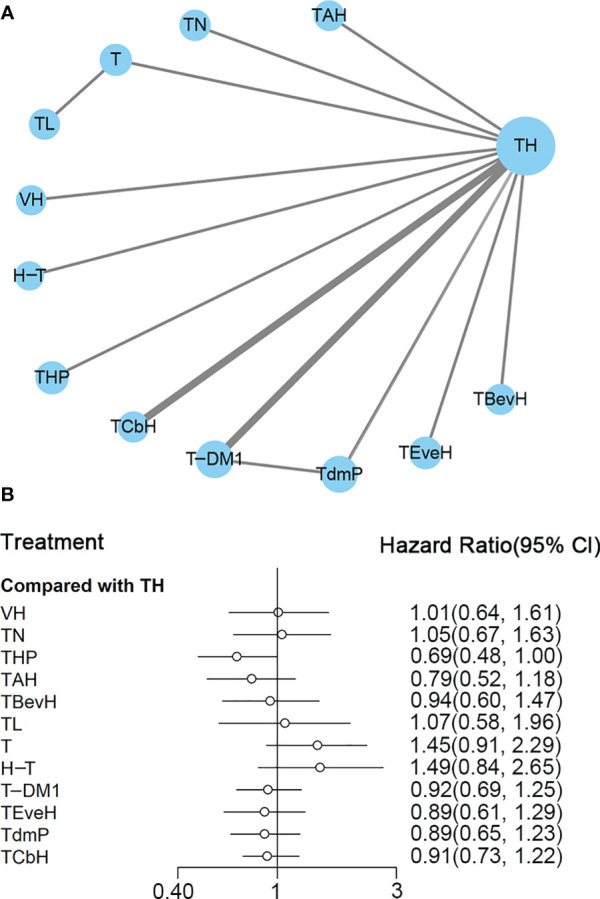
**(A)** Network plot of OS in first-line studies. **(B)** Forest plot of OS in first-line studies. Notes: The size of the blue node is proportional to the total number of participants assigned to each intervention. The width of the line is proportional to the number of trials comparing each pair of treatment. The thickness of the edge for connecting nodes means the amount of data. TH, Taxanes + Trastuzumab; VH, Trastuzumab + Vinorelbine; TCbH, Carboplatin + Taxanes + Trastuzumab; TdmP, Pertuzumab + trastuzumab emtansine; TEveH, Everolimus + Taxanes + Trastuzumab; T-DM1, trastuzumab emtansine; H-T, Sequential Trastuzumab → Docetaxel; TL, Lapatinib + Taxanes; TBevH, Bevacizumab + Taxanes + Trastuzumab; T, Taxanes; TAH, NPLD + Taxanes + Trastuzumab; THP, Pertuzumab + Taxanes + Trastuzumab; TN, Neratinib + Taxanes.

A total of 13 studies with 5,501 patients were included. The network plot is shown in **Figure 6A**.

In the network meta-analysis, there was no significant difference between TH (Taxanes + Trastuzumab) and other interventions, and details of network meta-analyses are indicated in **Figure 6B** and **Supplementary Data 11**. Network meta-analysis showed that THP (Pertuzumab + Taxanes + Trastuzumab) had better OS than T (Taxanes, HR 0.48, 95% CI 0.27–0.86) and H-T (Sequential Trastuzumab → Docetaxel, HR 0.46, 95% CI 0.24–0.91); however, there was no difference between other interventions (**Supplementary Data 11**). The results of direct comparisons are presented in **Supplementary Data 11**. THP (Pertuzumab + Taxanes + Trastuzumab, HR 0.69, 95% CI 0.58–0.82) and TCbH (Carboplatin + Taxanes + Trastuzumab, HR 0.90, 95% CI 0.88–0.92) had better OS than TH (Taxanes + Trastuzumab), and TH (Taxanes + Trastuzumab, HR 0.69, 95% CI 0.49–0.98) and TL (Lapatinib + Taxanes, HR 0.74, 95% CI 0.58–0.94) had better OS than T (Taxanes). There was no significant difference between other intervention groups.

**Figure 7:**
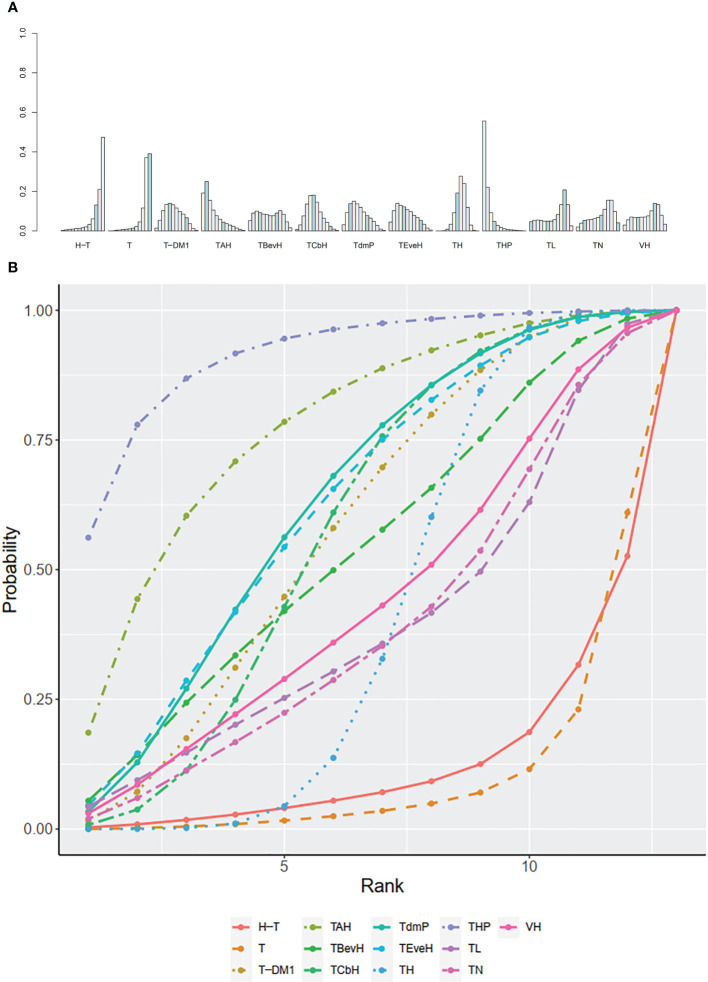
**(A)** Ranking histogram for OS in first-line studies. **(B)** Result of SUCRA for OS in first-line studies.

The ranking histogram showed that THP (Pertuzumab + Taxanes + Trastuzumab, 55.6% probability) ranked first, and TAH (NPLD + Taxanes + Trastuzumab, 25.0% probability) ranked second; H-T (Sequential Trastuzumab → Docetaxel, 47.4% probability) and T (Taxanes, 39.1% probability) ranked last (**Figure 7A**). The result of SUCRA showed that THP (Pertuzumab + Taxanes + Trastuzumab, 91.7% probability) ranked first, TAH (NPLD + Taxanes + Trastuzumab, 77.3% probability) ranked second, TdmP (Pertuzumab + trastuzumab emtansine 62.9% probability) ranked third, and T (Taxanes, 10.0% probability) ranked last (**Figure 7B**).

The trace plot and density plot showed a good convergence degree (**Supplementary Data 12A**). The inconsistency test result can be found in **Supplementary Data 12B**, except for those interventions where the loop is not able to be constructed.

##### Second- or Other-Line Treatment

**Figure 8:**
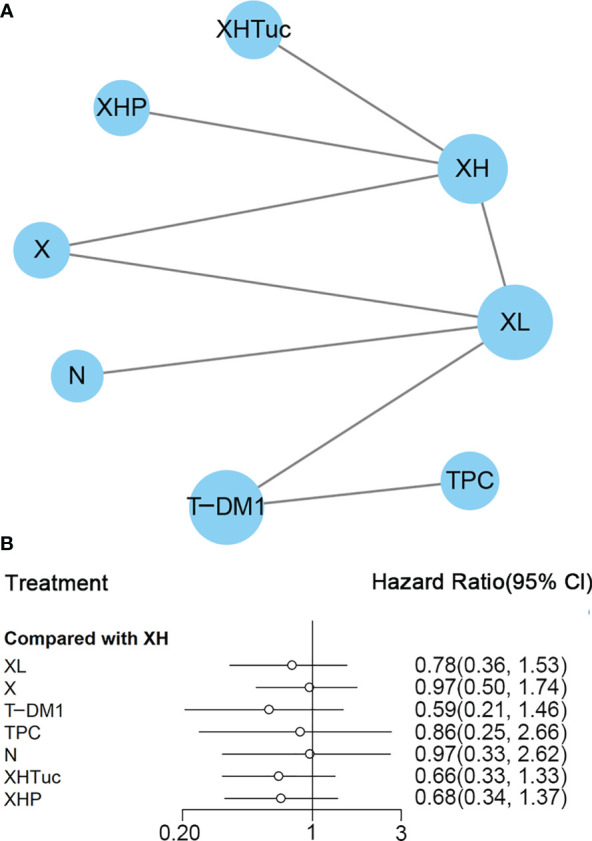
**(A)** Network plot of OS in second- or other-line studies. **(B)** Forest plot of OS in second or other line studies. Notes: The size of the blue node is proportional to the total number of participants assigned to each intervention. The width of the line is proportional to the number of trials comparing each pair of treatment. The thickness of the edge for connecting nodes means the amount of data. XH, Capecitabine + Trastuzumab; XHP, Capecitabine + Pertuzumab + Trastuzumab; XHTuc, Capecitabine + Trastuzumab + Tucatinib; N, Neratinib; TPC, Physician’s choice; T_DM1, trastuzumab emtansine; X, Capecitabine; XL, Capecitabine + Lapatinib.

A total of eight studies with 3,456 patients were included. The network plot is shown in **Figure 8A**.

In the network meta-analysis, there was no significant difference between XH (Capecitabine + Trastuzumab) and other interventions, and details of network meta-analyses are indicated in **Figure 8B** and **Supplementary Data 13**. Network meta-analyses showed no difference between interventions. The results of direct comparisons are presented in **Supplementary Data 13**. XHP (Capecitabine + Pertuzumab + Trastuzumab, HR 0.68, 95% CI 0.51–0.90) and XHTuc (Capecitabine + Trastuzumab + Tucatinib, HR 0.66, 95% CI 0.45–0.88) had better OS than XH (Capecitabine + Trastuzumab), T-DM1 had better OS than TPC (Physician’s choice, HR 0.68, 95% CI 0.54–0.85) and XL (Capecitabine + Lapatinib, HR 0.75, 95% CI 0.64–0.88), and there was no significant difference between other intervention groups.

**Figure 9:**
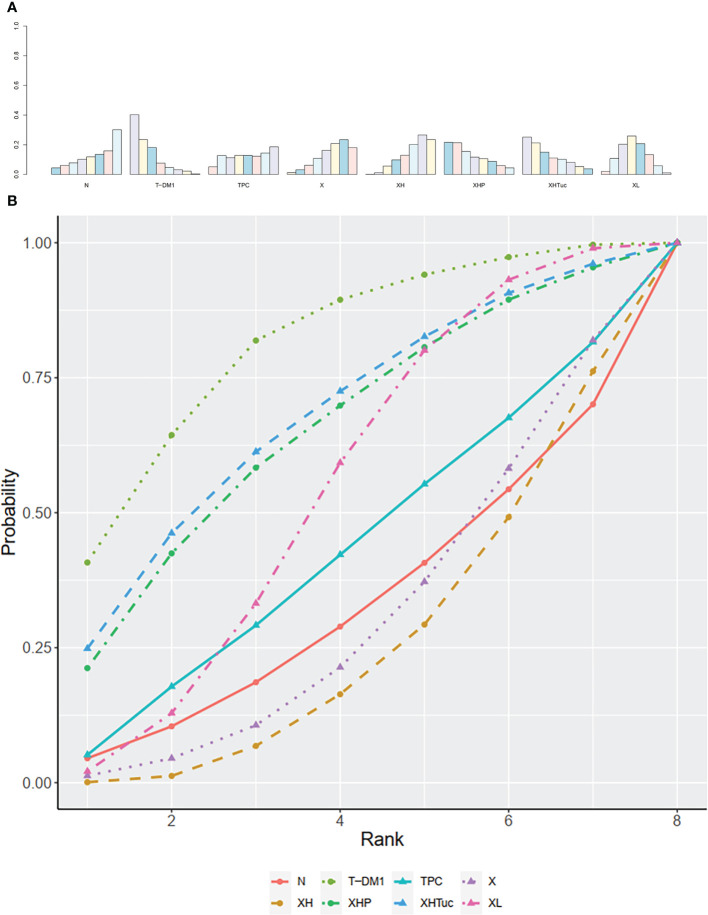
**(A)** Ranking histogram for OS in second- or other-line studies. **(B)** Result of SUCRA for OS in second- or other-line studies.

The ranking histogram showed that T-DM1 (trastuzumab emtansine, 40.2% probability), XHP (Capecitabine + Pertuzumab + Trastuzumab, 21.6% probability), and XHTuc (Capecitabine + Trastuzumab + Tucatinib, 25.2% probability) ranked first; N (Neratinib, 30.1% probability) and TPC (Physician’s choice, 18.6% probability) ranked last (**Figure 9A**). The result of SUCRA showed that T-DM1 (trastuzumab emtansine, 81.0% probability) ranked first, XHTuc (Capecitabine + Trastuzumab + Tucatinib, 68.0% probability) ranked second, XHP (Capecitabine + Pertuzumab + Trastuzumab, 65.6% probability) ranked third, and T (Taxanes, 25.6% probability) ranked last (**Figure 9B**).

The trace plot and density plot showed a good convergence degree (**Supplementary Data 14A**).

The inconsistency test result can be found in **Supplementary Data 14B**, except for those interventions where the loop is not able to be constructed.

##### Third- or Other-Line Treatment

OS outcome was not reported in Tolaney (45).

##### HR Subgroup Analysis

No HR subgroup analysis was done in the outcome of OS due to insufficient data.

#### Objective Response Rate

##### First-Line Treatment

**Figure 10:**
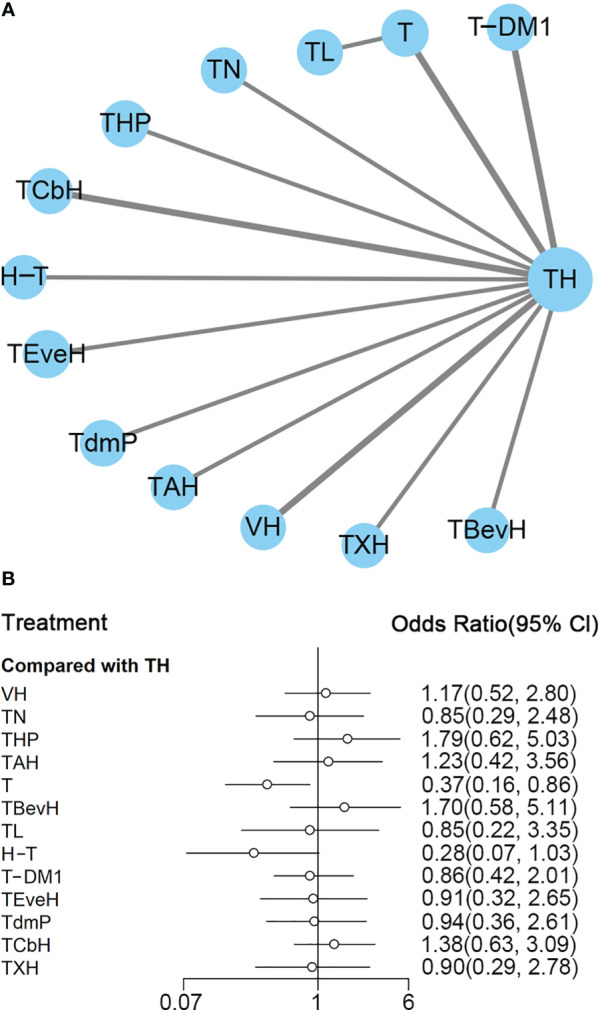
**(A)** Network plot of ORR in first-line studies. **(B)** Forest plot of ORR in first-line studies. Notes: The size of the blue node is proportional to the total number of participants assigned to each intervention. The width of the line is proportional to the number of trials comparing each pair of treatment. The thickness of the edge for connecting nodes means the amount of data. TH, Taxanes + Trastuzumab; TXH, Capecitabine + Taxanes + Trastuzumab; TCbH, Carboplatin + Taxanes + Trastuzumab; TEveH, Everolimus + Taxanes + Trastuzumab; T-DM1, trastuzumab emtansine; H-T, Sequential Trastuzumab → Docetaxel; TL, Lapatinib + Taxanes; TBevH, Bevacizumab + Taxanes + Trastuzumab; T, Taxanes, TAH, NPLD + Taxanes + Trastuzumab; THP, Pertuzumab + Taxanes + Trastuzumab; TN, Neratinib + Taxanes; VH, Trastuzumab + Vinorelbine; TdmP, Pertuzumab + trastuzumab emtansine.

A total of 16 studies with 5,935 patients were included. The network plot is shown in **Figure 10A**.

In the network meta-analysis, there was no significant difference between TH (Taxanes + Trastuzumab) and other interventions, except T (Taxanes, OR 0.37, 95% CI 0.16–0.86) with a lower outcome of ORR than TH. Details of network meta-analyses are indicated in **Figure 10B** and **Supplementary Data 15**. Network meta-analysis showed that THP (Pertuzumab + Taxanes + Trastuzumab) had higher ORR than H-T (Sequential Trastuzumab → Docetaxel, OR 6.36, 95% CI 1.22–34.45) and T (Taxanes, OR 4.91, 95% CI 1.23–18.32); however, H-T (Sequential Trastuzumab → Docetaxel) had lower ORR than TCbH (HR 0.20, 95% CI 0.04–0.92) (**Supplementary Data 15**). The results of direct comparisons are presented in **Supplementary Data 15**. Results from direct comparisons showed that THP (Pertuzumab + Taxanes + Trastuzumab, OR 1.79, 95% CI 1.26–2.55) and TBevH (Bevacizumab + Taxanes + Trastuzumab, OR 1.69, 95% CI 1.06–2.69) had higher ORR than TH, while TH had higher ORR than H-T (Sequential Trastuzumab → Docetaxel, OR 3.57, 95% CI 1.47–9.09) and T (Taxanes, OR 2.70, 95% CI 1.69–4.35). TL (Lapatinib + Taxanes) had higher ORR than T (Taxanes, OR 2.32, 95% CI 1.57–3.42). There was no significant difference between other intervention groups.

**Figure 11:**
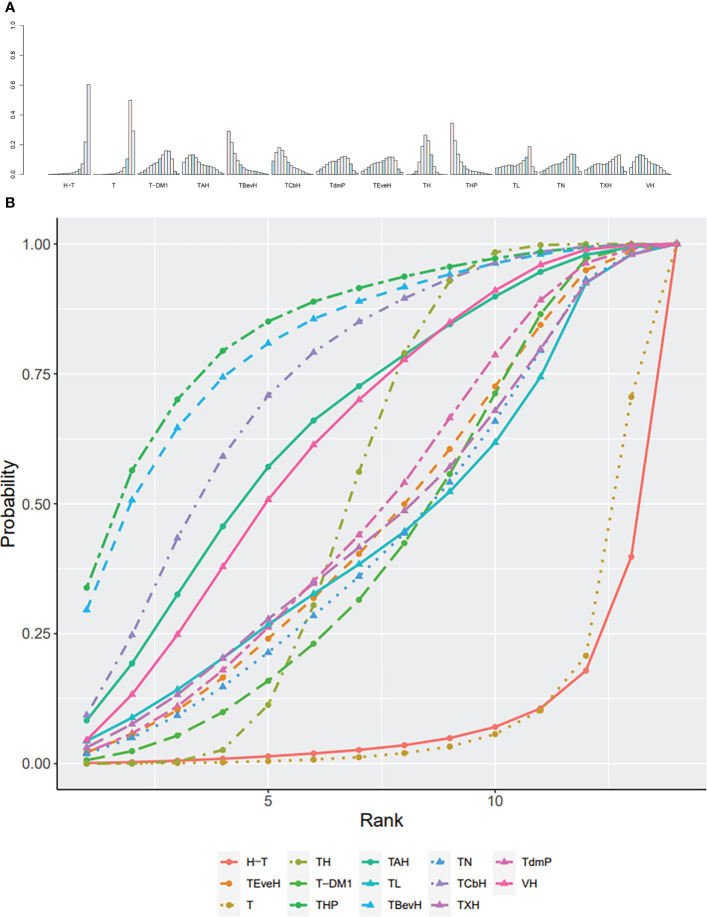
**(A)** Ranking histogram for ORR in first-line studies. **(B)** Result of SUCRA for ORR in first-line studies.

The ranking histogram showed that THP (Pertuzumab + Taxanes + Trastuzumab, 34.4% probability) and TBevH (Bevacizumab + Taxanes + Trastuzumab, 29.1% probability) ranked first and TCbH (Carboplatin + Taxanes + Trastuzumab, 18.1% probability) ranked third; H-T (Sequential Trastuzumab → Docetaxel, 60.4% probability) ranked last (**Figure 11A**). The result of SUCRA showed that THP (Pertuzumab + Taxanes + Trastuzumab, 84.1% probability) ranked first, TBevH (Bevacizumab + Taxanes + Trastuzumab, 81.2% probability) ranked second, TCbH (Carboplatin + Taxanes + Trastuzumab, 72.7% probability) ranked third, and H-T (Sequential Trastuzumab → Docetaxel, 6.8% probability) ranked last (**Figure 11B**).

The trace plot and density plot showed a good convergence degree (**Supplementary Data 16**).

The inconsistency test is not available as the no-intervention loop was constructed.

##### Second- or Other-Line Treatment

**Figure 12:**
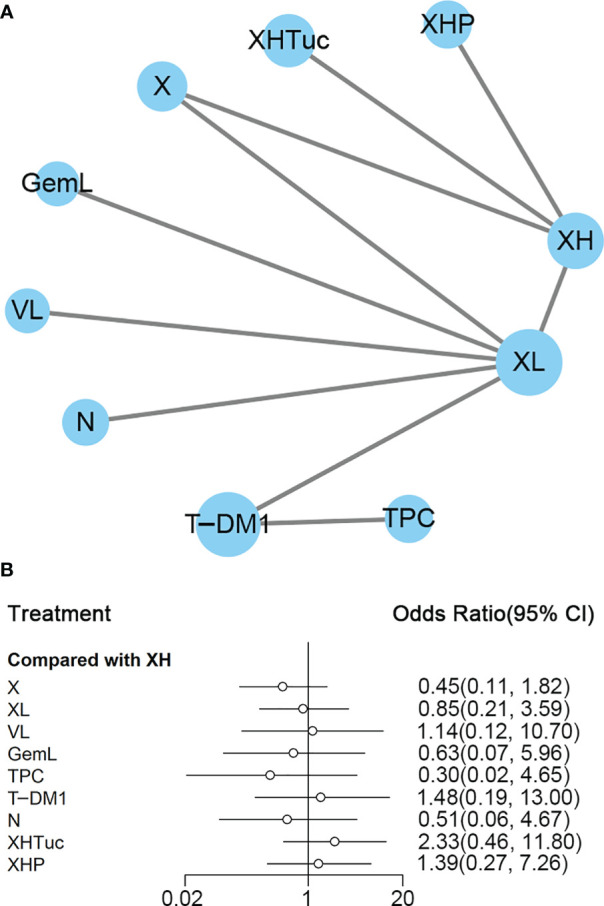
**(A)** Network plot of ORR in second- or other -line studies. **(B)** Forest plot of ORR in second- or other-line studies. Notes: The size of the blue node is proportional to the total number of participants assigned to each intervention. The width of the line is proportional to the number of trials comparing each pair of treatment. The thickness of the edge for connecting nodes means the amount of data. XH, Capecitabine + Trastuzumab; XHP, Capecitabine + Pertuzumab + Trastuzumab; XHTuc, Capecitabine + Trastuzumab + Tucatinib; N, Neratinib; LTop, Lapatinib + topotecan; TPC, Physician’s choice; T-DM1, trastuzumab emtansine; VL, Lapatinib + Vinorelbine; XL, Capecitabine + Lapatinib; X, Capecitabine.

A total of nine studies with 3,598 patients were included (**Figure 12A**). CNS ORR reported in the Lin (42) study was defined as a ≥50% volumetric reduction of CNS lesion(s) in the absence of new or progressive CNS or non-CNS lesions, or increasing steroid requirements was not used for ORR analysis in this network.

In the network meta-analysis, there was no significant difference between XH (Capecitabine + Trastuzumab) and other interventions, and details of network meta-analyses are indicated in **Figure 12B** and **Supplementary Data 17**. Network meta-analyses showed no difference between interventions. The results of direct comparisons are presented in **Supplementary Data 17**. There was no significant difference between groups, except that T-DM1 had higher ORR than XL (Capecitabine + Lapatinib, OR 1.74, 95% CI 1.29–2.33) and TPC (Physician’s choice, OR 5.00, 95% CI 2.73–9.17); XH (Capecitabine + Trastuzumab) had higher ORR than X (Capecitabine, OR 2.53, 95% CI 1.28–5.02); XHTuc (Capecitabine + Trastuzumab + Tucatinib, OR 2.31, 95% CI 1.52–3.51) had higher ORR than XH (Capecitabine + Trastuzumab).

**Figure 13:**
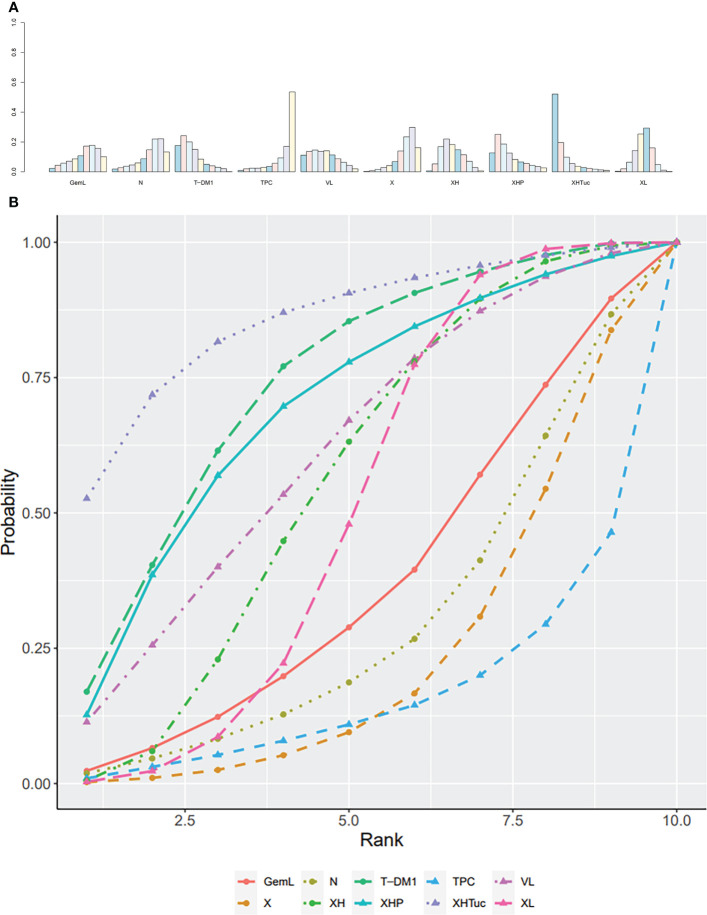
**(A)** Ranking histogram for ORR in second- or other-line studies. **(B)** Result of SUCRA for ORR in second- or other-line studies.

The ranking histogram showed that XHTuc (Capecitabine + Trastuzumab + Tucatinib, 52.2% probability) ranked first, and XHP (Capecitabine + Pertuzumab + Trastuzumab, 25.1% probability) and T-DM1 (trastuzumab emtansine, 24.3% probability) ranked second; TPC (Physician’s choice, 53.6% probability) ranked last (**Figure 13A**). The result of SUCRA showed that XHTuc (Capecitabine + Trastuzumab + Tucatinib, 84.9% probability) ranked first, T-DM1 (trastuzumab emtansine, 73.5% probability) ranked second, XHP (Capecitabine + Pertuzumab + Trastuzumab, 68.3% probability) ranked third, and TPC (Physician’s choice, 18.9% probability) ranked last (**Figure 13B**).

The trace plot and density plot showed a good convergence degree (**Supplementary Data 18A**).

The inconsistency test result can be found in **Supplementary Data 18B**, except for those interventions where the loop is not able to be constructed.

##### Third- or Other-Line Treatment

In the Tolaney (45) study, comparing Abemaciclib + Fulvestrant + Trastuzumab *versus* Abemaciclib + Trastuzumab *versus* SOC chemotherapy + Trastuzumab, ORR at a median 19-month follow-up in each group was 33%, 14%, and 14%, respectively, showing a significant advantage in the Abemaciclib + Fulvestrant + Trastuzumab group.

##### HR Subgroup Analysis

No HR subgroup analysis was done in the outcome of ORR due to insufficient data.

#### Safety Outcomes

##### First-Line Treatment

**Figure 14:**
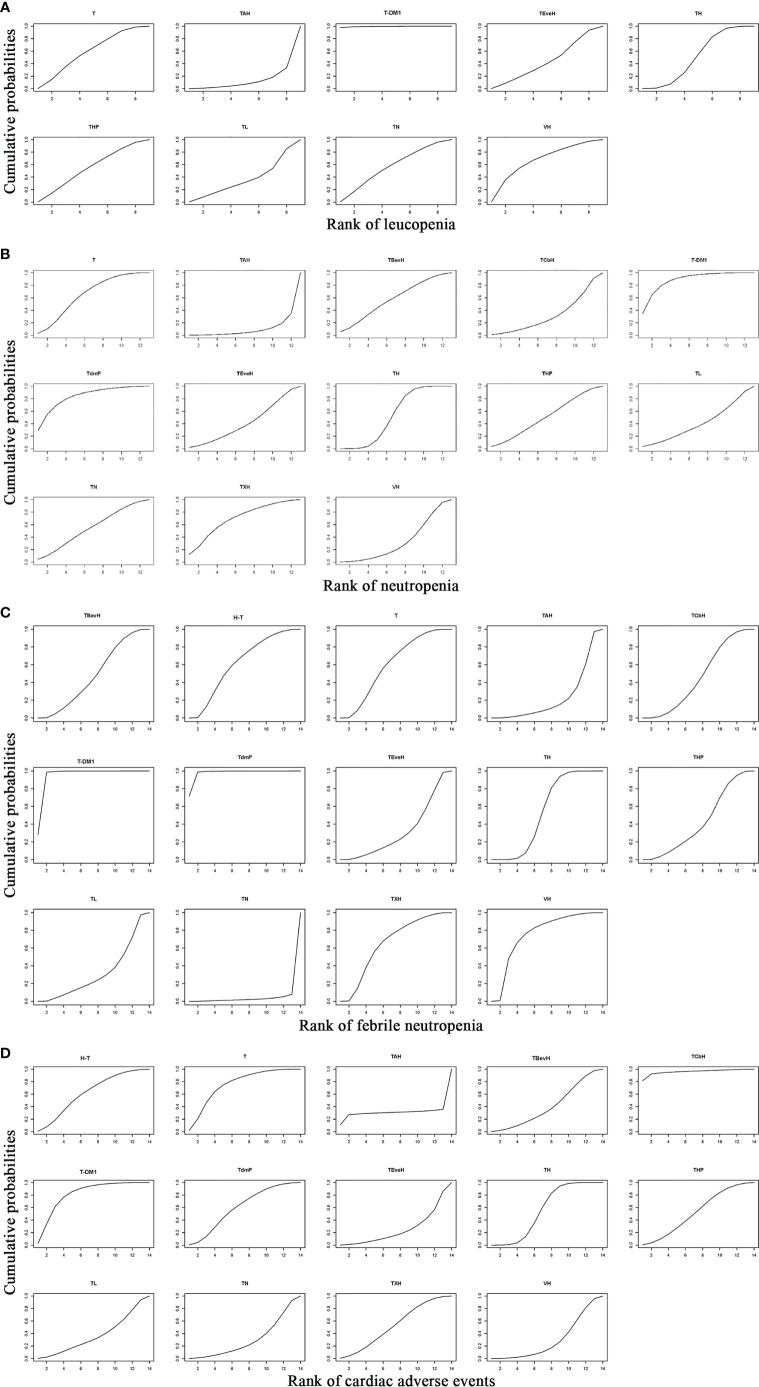
Result of SUCRA for **(A)** leucopenia, **(B)** neutropenia, **(C)** febrile neutropenia, and **(D)** cardiac adverse events. Notes: The larger the SUCRA, the lower the probability of adverse events. H-T, Sequential Trastuzumab → Docetaxel; T, Taxanes; TAH, NPLD + Taxanes + Trastuzumab; TBevH: Bevacizumab + Taxanes + Trastuzumab; TCbH, Carboplatin + Taxanes + Trastuzumab; T-DM1, trastuzumab emtansine; TdmP, Pertuzumab + trastuzumab emtansine; TEveH, Everolimus + Taxanes + Trastuzumab; TH, Taxanes + Trastuzumab; THP, Pertuzumab + Taxanes + Trastuzumab; TL, Lapatinib + Taxanes; TN, Neratinib + Taxanes; TXH, Capecitabine + Taxanes + Trastuzumab; VH, Trastuzumab + Vinorelbine.

For leucopenia, the result of SUCRA showed that T-DM1 (trastuzumab emtansine, 99.5% probability) ranked first, VH (Trastuzumab + Vinorelbine, 63.4% probability) ranked second, T (Taxanes, 55.1% probability) ranked third, and TAH (NPLD + Taxanes + Trastuzumab, 9.7% probability) ranked last (**Figure 14A**).

For neutropenia, the result of SUCRA showed that T-DM1 (trastuzumab emtansine, 87.4% probability) ranked first, TdmP (Pertuzumab + trastuzumab emtansine, 82.5% probability) ranked second, TXH (Capecitabine + Taxanes + Trastuzumab, 67.8% probability) ranked third, and TAH (NPLD + Taxanes + Trastuzumab, 7.6% probability) ranked last (**Figure 14B**).

For febrile neutropenia, the result of SUCRA showed that TdmP (Pertuzumab + trastuzumab emtansine, 97.7% probability) ranked first, T-DM1 (trastuzumab emtansine, 94.2% probability) ranked second, VH (Trastuzumab + Vinorelbine, 72.2% probability) ranked third, and TN (Neratinib + Taxanes, 2.5% probability) ranked last (**Figure 14C**).

For cardiac adverse events, the result of SUCRA showed that TCbH (Carboplatin + Taxanes + Trastuzumab, 95.7% probability) ranked first, T-DM1 (Trastuzumab emtansine, 79.9% probability) ranked second, T (Taxanes, 73.7% probability) ranked third, and TEveH (Everolimus + Taxanes + Trastuzumab, 22.9% probability) ranked last (**Figure 14D**).

##### Second- or Other-Line Treatment

For leucopenia, the result of SUCRA showed that XH (Capecitabine + Trastuzumab, 98.9% probability) ranked first and GemL (Gemcitabine + Lapatinib, 16.3% probability) ranked last (**Supplementary Data 19A**).

For neutropenia, the result of SUCRA showed that T-DM1 (Trastuzumab emtansine, 76.4% probability) ranked first and GemL (Gemcitabine + Lapatinib, 10.1% probability) ranked last (**Supplementary Data 19B**).

For febrile neutropenia, the result of SUCRA showed that T-DM1 (Trastuzumab emtansine, 86.7% probability) ranked first and VL (Lapatinib + Vinorelbine, 7.0% probability) ranked last (**Supplementary Data 19C**).

For cardiac adverse events, the result of SUCRA showed that X (Capecitabine, 71.5% probability) ranked first, T-DM1 (Trastuzumab emtansine, 56.1% probability) ranked second, and XHP (Capecitabine + Pertuzumab + Trastuzumab, 26.9% probability) ranked last (**Supplementary Data 19D**).

##### Third- or Other-Line Treatment

For leucopenia, the result of SUCRA showed that Habe (Abemaciclib + Trastuzumab, 92.9% probability) ranked first, CTH (SOC chemotherapy + Trastuzumab, 30.6% probability) ranked second, and FHAbe (Abemaciclib + Fulvestrant + Trastuzumab, 26.5% probability) ranked last.

For neutropenia, the result of SUCRA showed that Habe (Abemaciclib + Trastuzumab, 73.0% probability) ranked first, CTH (SOC chemotherapy + Trastuzumab, 40.6% probability) ranked second, and FHAbe (Abemaciclib + Fulvestrant + Trastuzumab, 36.4% probability) ranked last.

For febrile neutropenia, the result of SUCRA showed that Habe (Abemaciclib + Trastuzumab, 98.6% probability) ranked first, FHAbe (Abemaciclib + Fulvestrant + Trastuzumab, 42.2% probability) ranked second, and CTH (SOC chemotherapy + Trastuzumab, 9.2% probability) ranked last.

For cardiac adverse events, the result of SUCRA showed that FHAbe (Abemaciclib + Fulvestrant + Trastuzumab, 86.1% probability) ranked first, CTH (SOC chemotherapy + Trastuzumab, 63.1% probability) ranked second, and Habe (Abemaciclib + Trastuzumab, 0.8% probability) ranked last.

### Publication Bias and Sensitivity Analyses

The funnel plots were not performed because the number of included studies in one comparison was less than 10. Overall, sensitivity analyses showed that the results were stable.

## Discussion

In our network meta-analysis, we compared PFS, OS, and ORR of previously reported HER-2 targeted treatments, including first-line, second-line, and third- or other-line treatments. We conducted ranking histogram and SUCRA for each efficacy outcome. The ranking histogram showed the probability of each ranking of a regimen and the result of SUCRA showed the ranking of all involved regimens. The results of ranking histogram and SUCRA showed good consistency in the meta-analysis.

The network meta-analysis revealed that THP (Pertuzumab + Taxanes + Trastuzumab) ranked first in both ranking probability and SUCRA in PFS, OS, and ORR among first-line treatments. Regimens containing other anti-HER2 agents, such as TL (lapatinib + taxanes), TN (neratinib + taxanes), and T-DM1 and TdmP (T-DM1 + pertuzumab) were inferior to THP in all efficacy outcomes, suggesting that THP was still the optimal option to reach the best efficacy for metastatic HER2+ BC. The anti-tumor mechanism of Trastuzumab is binding to subdomain 4 of HER2 extracellular domain to activate antibody-dependent cellular cytotoxicity (ADCC) and cellular phagocytosis and directly suppress HER2, thereby inhibiting HER2-overexpressing tumor cells (46). Pertuzumab binds to subdomain 2, a different site of HER2 extracellular domain than trastuzumab, to inhibit overexpression of HER2. Therefore, adding pertuzumab to trastuzumab provides dual blockade of HER2 and enhanced antitumor efficacy (47). In CLEOPATRA, the combination of pertuzumab, trastuzumab, and docetaxel significantly improved PFS and OS in comparison with trastuzumab plus docetaxel in metastatic BC patients (5). In PUFFIN (48), the efficacy of THP was also verified in Chinese patients. For safety outcomes, THP exhibited moderate risk of hematologic and cardiac toxicity compared with other first-line therapies. The frequent SAEs of THP were neutropenia (49%), leukopenia (12%), and diarrhea (10%) in CLEOPATRA (44).

For second-line therapies, the efficacy of T-DM1 and XHTuC was superior to other regimens according to the network meta-analysis. In both ranking probability and SUCRA results, T-DM1 ranked first in both PFS and OS and the second in ORR. T-DM1 is an antibody–drug conjugate of trastuzumab linked to the cytotoxic agent maytansinoid (DM1) (49). T-DM1 not only retains the anti-tumor function of trastuzumab, but also delivers microtubule destabilizer DM1 to targeted tumor cells (46). It is recommended for the second-line therapy of metastatic HER2-positive BC according to the EMILIA trial (6). Our results confirmed that T-DM1 could significantly improve PFS and OS compared with other second-line therapies. T-DM1 also exhibited lower risk of hematologic and cardiac toxicity when comparing with other second- or later-line therapies, suggesting excellent safety of T-DM1.The frequent SAEs of T-DM1 were thrombocytopenia (14%), increased AST (4%), anemia (4%), and increased ALT (3%) in EMILIA (50).

Tucatinib is an oral HER2-specific tyrosine kinase inhibitor. It is highly selective for HER2 and inhibits epidermal growth factor receptor (EGFR) minimally (51). In the network meta-analysis, XHTuC (capecitabine + trastuzumab + tucatinib) ranked first in SUCRA for ORR and second in SUCRA for PFS and OS, showing an excellent effect in striking HER2-overexpressing tumor cells among second-line therapies. In the HER2CLIMB trial (43), XHTuC was conducted in HER2+ metastatic BC patients who underwent previous treatments of trastuzumab, pertuzumab, and T-DM1. Adding tucatinib to capecitabine and trastuzumab exhibited enhanced PFS (7.8 months *versus* 5.6 months), OS (21.9 months *versus* 17.4 months), and ORR (40.6% *versus* 22.8%) in comparison with the combination of capecitabine and trastuzumab. For patients with brain metastases, XHTuC also significantly improved PFS (7.6 months *versus* 5.4 months) compared with XH (Capecitabine + Trastuzumab) (43). These results suggested that XHTuC might be a good candidate for second-line therapy, especially for brain metastatic BC. The SAEs of XHTuC reported by HER2CLIMB were palmar–plantar erythrodysesthesia syndrome (13.1%), diarrhea (12.9%), increased ALT (5.4%), fatigue (4.7%), and increased AST (4.5%) (43). When comparing with other second- or later-line therapies, the risk of hematologic and cardiac toxicity of XHTuc was moderate. However, the clinical trial regarding tucatinib is still limited and further research including the direct comparison of tucatinib regimen and other therapies is still needed.

Previous research suggested that alterations to anti-HER2 agents and chemotherapeutic drugs might both be effective for patients who did not benefit from first-line therapies. We performed the network meta-analysis to figure out which alteration was better for patients with previous THP treatment. The results showed that changing anti-HER2 agents to T-DM1 or tucatinib exhibited better efficacy than other second-line treatments. However, drug resistance still exists in second-line therapies and new agents with minimal drug resistance are still needed.

For third- or other-line therapies, there was only one study (monarcHER) included so the network meta-analysis was unable to perform. The monarcHER study revealed that FHAbe (abemaciclib + fulvestrant + trastuzumab) improved PFS and OS compared with CTH (chemotherapy + trastuzumab) in HR+/HER2+ advanced BC patients who had received at least two previous anti-HER2 treatments (45). The anti-tumor mechanism of Abemaciclib is to inhibit the CDK4/6 pathway, which mediated drug resistance to anti-HER2 agents (52). The frequent SAE of FHAbe included neutropenia (27%), leucopenia (10%), thrombocytopenia (10%), diarrhea (9%), and anemia (9%) (45). The standard of third-line therapies has not been well defined and more research is needed to evaluate the efficacy of third-line treatments.

Notably, some recently approved anti-HER2 agents exhibited excellent efficacy in single-arm studies. For example, trastuzumab deruxtican (DS-8201), an antibody–drug conjugate, achieved a median PFS of 16.4 months and an ORR of 60.9% when applied to metastatic BC patients who had received trastuzumab and T-DM1 in DESTINY-Breast01 (53). The promising effect suggested the application of trastuzumab deruxtican in third-line and even first- or second-line therapies. However, interstitial lung diseases were observed in 13.6% of the patients, and the safety property should be further evaluated.

According to the expression of HR, HER2+ BC can be divided into HR+/HER2+ and HR−/HER2+ BC. In HR+/HER2+ BC, the signaling pathway of HR and HER2 has complex crosstalk between each other (54). On the one hand, the crosstalk between HR and HER2 decreases the efficacy of endocrine therapy. On the other hand, the expression of HR is associated with resistance to anti-HER2 agents (55). Therefore, it is believed that combining endocrine therapy with anti-HER2 therapy could decrease drug resistance to both therapies (56). In a metastatic HER2+ BC setting, THP was the best option in first-line therapy and T-DM1 was the best option in second-line therapy in PFS outcomes according to SUCRA, regardless of HR status. So, HR status did not impact the standard of anti-HER2 agents and chemotherapy in metastatic HER2+ BC in first- and second-line therapies, while in third- or later-line therapies, the regimen of FHAbe was restricted to HR+ BC in monarcHER (45). Besides, the combination of endocrine therapy and anti-HER2 agents was an alternative for patients with metastatic HR+/HER2+ BC who did not tolerate chemotherapy in third- or later-line therapies.

For adverse events, hematologic adverse events (leucopenia, neutropenia, and febrile neutropenia) and cardiac adverse events were included in the network meta-analysis. Other adverse events were not included because of insufficient data. In first-line therapies, TAH and TN exhibited the highest risk of hematologic toxicity and TEveH exhibited the highest risk of cardiac toxicity, whereas T-DM1 ranked top two in all adverse events, showing the greatest safety. However, THP could achieve the best efficacy among first-line therapies with acceptable safety, and it was still recommended as the standard first-line therapy by synthesizing efficacy and safety results. In second-line therapies, T-DM1 also performed the best in safety outcomes, whereas GemL and VL showed the highest hematologic toxicity and XHP showed the highest cardiac toxicity. Considering efficacy and safety results, T-DM1 was the best option in second-line therapies while XHTuC was still recommended with excellent efficacy and acceptable safety. In third-line therapies, the risk of hematologic toxicity was higher while the risk of cardiac adverse events was lower for FHAbe compared with HAbe, and FHAbe was still recommended considering efficacy outcomes.

A strength of our study is that regimens of the combination of anti-HER2 agents and chemotherapy were included in the analysis so that the efficacy and safety outcomes of all these regimens could be compared. The first, second, and third or later treatment lines were all analyzed. Besides, the hematological toxicity and cardiac adverse events were also included in the network meta-analysis.

There are several limitations in the study. First, a small amount of locally advanced BC patients was included in some RCTs in our study and could not be excluded, leading to a slight heterogeneity. However, the proportion of locally advanced BC patients was low and the heterogeneity was acceptable. Second, 16 studies were open-label or did not mask for participants and investigators and were rated as high risk of bias in blinding of participants and investigators. The results could be influenced by subjectivity and should be interpreted cautiously. Third, some RCTs of new anti-HER2 agents were excluded because of the mixture of different treatment lines. For example, a phase II study regarding pyrotinib was not included because it contained patients with a previous treatment of trastuzumab and those without (57). Further related studies with specific treatment groups are needed.

In conclusion, THP is still recommended for the standard first-line therapy for metastatic HER2+ BC, regardless of HR status. For second-line therapies, T-DM1 and XHTuC exhibit better efficacy than other regimens and acceptable safety compared with other second-line therapies. Further clinical trials are still needed, especially for second- or later-line therapies.

## Data Availability Statement

The original contributions presented in the study are included in the article/**Supplementary Material**. Further inquiries can be directed to the corresponding author.

## Author Contributions

XZ and JL conceived and designed the research. XZ and JL prepared the manuscript. YZ, FM, YL, and SS collected the data. XZ and JL analyzed the data. QS made the final revisions. All authors contributed to the article and approved the submitted version.

## Conflict of Interest

The authors declare that the research was conducted in the absence of any commercial or financial relationships that could be construed as a potential conflict of interest.

## Publisher’s Note

All claims expressed in this article are solely those of the authors and do not necessarily represent those of their affiliated organizations, or those of the publisher, the editors and the reviewers. Any product that may be evaluated in this article, or claim that may be made by its manufacturer, is not guaranteed or endorsed by the publisher.

## Abbreviations

AE, adverse event; BC, breast cancer; CENTRAL, Cochrane Central Register of Controlled Trials; CLEOPATRA, Clinical Evaluation of Pertuzumab and Trastuzumab trial; CrI, credible interval; CT+H, SOC chemotherapy + Trastuzumab; DS-8201, Trastuzumab deruxtecan; ECOG PS, Eastern Cooperative Oncology Group performance status; ER, (o)estrogen receptor; FHAbe, Abemaciclib + Fulvestrant + Trastuzumab; FISH, fluorescence *in situ* hybridization; H, Trastuzumab; HAbe, Abemaciclib + Trastuzumab; HER2, human epidermal growth factor receptor 2; HR, hazard ratio; HR+, hormone receptor positive; HR−, hormone receptor negative; H-T, Sequential Trastuzumab → Docetaxel; IHC, immunohistochemistry; IQR, interquartile range; LTop, Lapatinib+topotecan; MD, mean difference; N, Neratinib; NPLD, nonpegylated liposomal doxorubicin; NR, not reported; N/A, not available; OR, odds ratio; ORR, objective response rate; OS, overall survival; PFS, progression-free survival; PR, progesterone receptor; PRISMA, preferred reporting items for systematic review and meta-analysis; RCT, randomized controlled trials; ROB, risk of bias; SAE, serious adverse event; SD, standard difference; SOC, standard of care; SUCRA, surface under the cumulative ranking curve; T, Taxanes; TAH, NPLD + Taxanes + Trastuzumab; TBevH, Bevacizumab + Taxanes + Trastuzumab; TCbH, Carboplatin + Taxanes + Trastuzumab; T-DM1, trastuzumab emtansine; TdmP, Pertuzumab+trastuzumab emtansine; TEveH, Everolimus + Taxanes + Trastuzumab; TH, Taxanes + Trastuzumab; THP, Pertuzumab + Taxanes + Trastuzumab; TL, Lapatinib + Taxanes; TN, Neratinib+Taxanes; TPC, Physician’s choice; TTP, time to progression; TXH, Capecitabine + Taxanes + Trastuzumab; VH, Trastuzumab+Vinorelbine; VL, Lapatinib + Vinorelbine; X, Capecitabine; XH, Capecitabine + Trastuzumab; XHP, Capecitabine + Pertuzumab + Trastuzumab; XHTuc, Capecitabine + Trastuzumab + Tucatinib; XL, Capecitabine+Lapatinib.
