# Supplementary material for: Efficacy and Safety of Anti-HER2 Agents in Combination With Chemotherapy for Metastatic HER2-Positive Breast Cancer Patient: A Network Meta-Analysis
Source: Front Oncol. 2021 Aug 19;11:731210. doi: 10.3389/fonc.2021.731210 (PMC8416996; doi:10.3389/fonc.2021.731210)
Supplement: Supplementary file 1 [file DataSheet_1.zip › Supplementary data 5E Trace plot and density plot of PFS in first line HR+ studies.pdf]

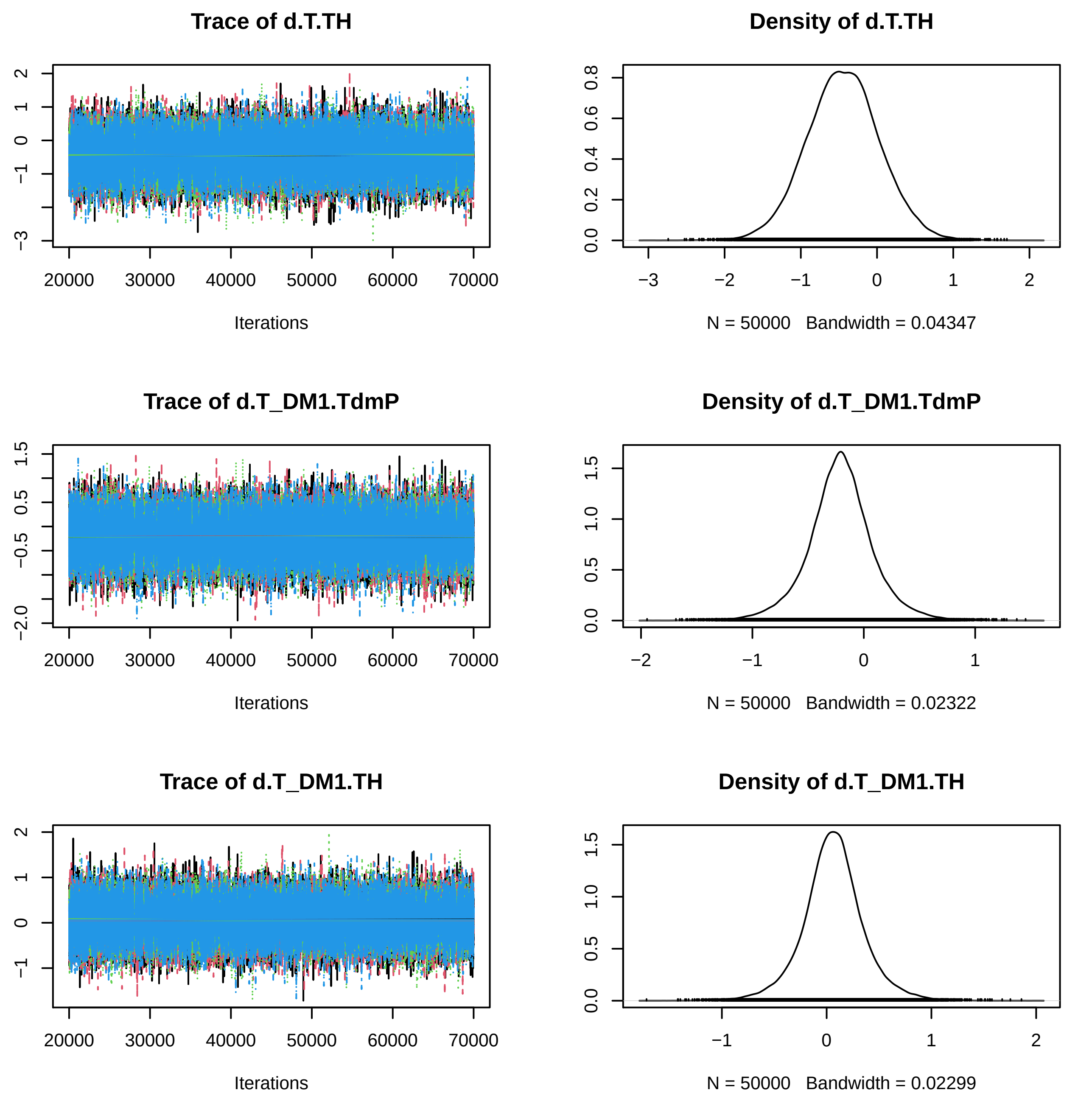
Trace of d.T.TH
Iterations
Density of d.T.TH
N = 50000 Bandwidth = 0.04347
Trace of d.T_DM1.TdmP
Iterations
Density of d.T_DM1.TdmP
N = 50000 Bandwidth = 0.02322
Trace of d.T_DM1.TH
Iterations
Density of d.T_DM1.TH
N = 50000 Bandwidth = 0.02299

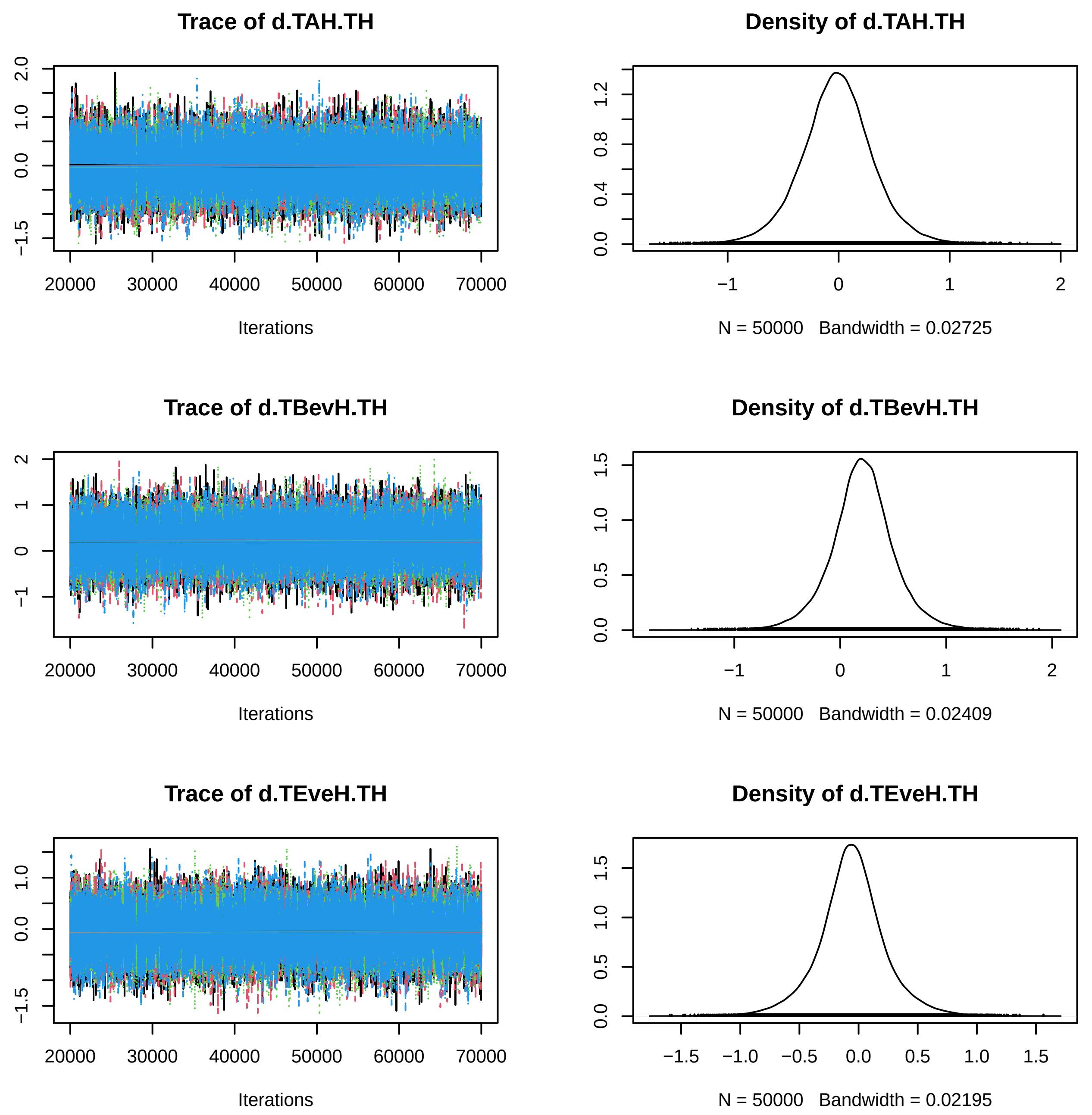
Trace of d.TAH.TH
Iterations
N = 50000 Bandwidth = 0.02725
Density of d.TAH.TH
Trace of d.TBevH.TH
Iterations
Density of d.TBevH.TH
N = 50000 Bandwidth = 0.02409
Trace of d.TEveH.TH
Iterations
Density of d.TEveH.TH
N = 50000 Bandwidth = 0.02195

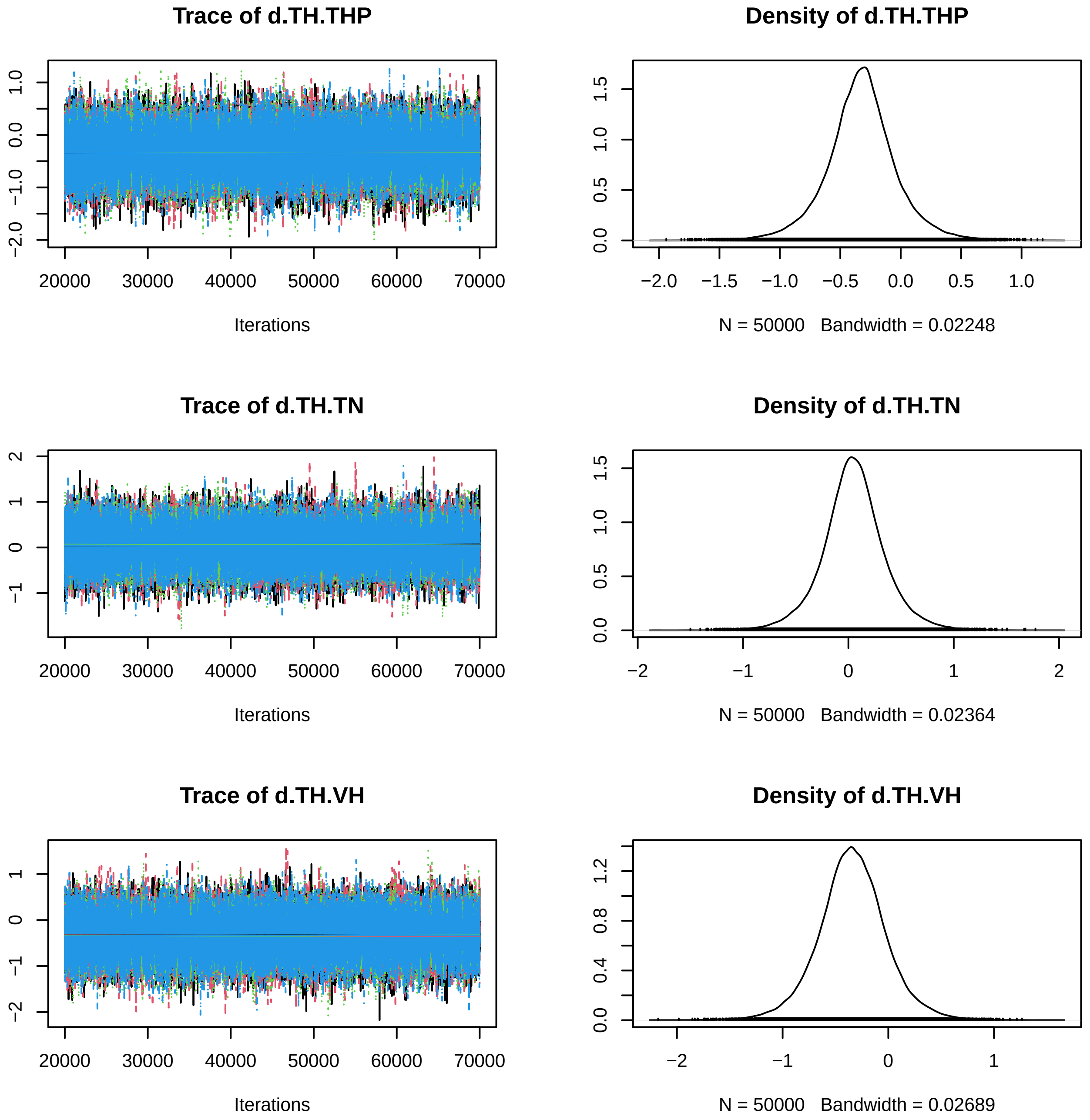
Trace of d.TH.THP
Density of d.TH.THP
Iterations
N = 50000 Bandwidth = 0.02248
Trace of d.TH.TN
Density of d.TH.TN
Iterations
N = 50000 Bandwidth = 0.02364
Trace of d.TH.VH
Density of d.TH.VH
Iterations
N = 50000 Bandwidth = 0.02689
20000
30000
40000
50000
60000
70000

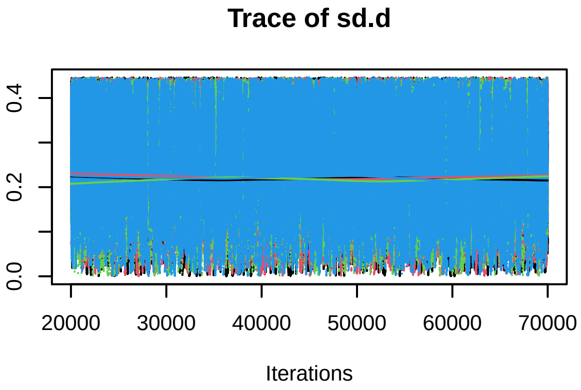
Trace of sd.d
0.0
0.2
0.4
20000
30000
40000
50000
60000
70000
Iterations

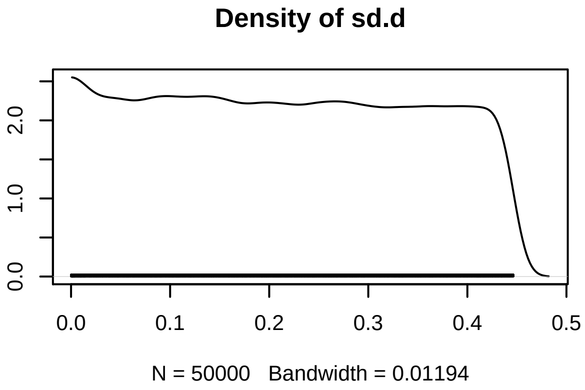
Density of sd.d
0.0
1.0
2.0
0.0
0.1
0.2
0.3
0.4
0.5
N = 50000   Bandwidth = 0.01194
