# Supplementary material for: Efficacy and Safety of Anti-HER2 Agents in Combination With Chemotherapy for Metastatic HER2-Positive Breast Cancer Patient: A Network Meta-Analysis
Source: Front Oncol. 2021 Aug 19;11:731210. doi: 10.3389/fonc.2021.731210 (PMC8416996; doi:10.3389/fonc.2021.731210)
Supplement: Supplementary file 1 [file DataSheet_1.zip › Supplementary data 1 Search strategy.docx]

Supplementary data 1 Search strategy

Search date: 30-5-2020

PubMed (638)

<http://www.ncbi.nlm.nih.gov/pubmed?otool=leiden>

(("Trastuzumab"[Mesh] OR "pertuzumab"[Supplementary Concept] OR "Ado-Trastuzumab Emtansine"[Mesh] OR "Lapatinib"[Mesh] OR "pyrotinib"[Supplementary Concept] OR "Afatinib"[Mesh] OR "neratinib"[Supplementary Concept] OR "margetuximab"[Supplementary Concept] OR "tucatinib"[Supplementary Concept] OR "trastuzumab deruxtecan"[Supplementary Concept] OR "trastuzumab"[tw] OR "Herceptin"[tw] OR "Trazimera"[tw] OR "pertuzumab"[tw] OR "Perjeta"[tw] OR "rhumab 2C4"[tw] OR "rhumab-2C4"[tw] OR "Omnitarg"[tw] OR "T-DM1"[tw] OR "TDM1"[tw] OR "Ado Trastuzumab Emtansine"[tw] OR "Trastuzumab Emtansine"[tw] OR "Kadcyla"[tw] OR "huN901-DM1"[tw] OR "huN901 DM1"[tw] OR "Trastuzumab-DM1 Conjugate"[tw] OR "Trastuzumab DM1 Conjugate"[tw] OR "Trastuzumab-DM1"[tw] OR "Trastuzumab DM1"[tw] OR "lapatinib"[tw] OR "Tykerb"[tw] OR "GW282974X"[tw] OR "GW572016"[tw] OR "GW-572016"[tw] OR "GW 572016"[tw] OR "pyrotinib"[tw] OR "afatinib"[tw] OR "Afatinib Maleate"[tw] OR "BIBW 2992"[tw] OR "BIBW2992"[tw] OR "BIBW-2992"[tw] OR "Gilotrif"[tw] OR "neratinib"[tw] OR "Nerlynx"[tw] OR "HKI 272"[tw] OR "HKI272"[tw] OR "HKI-272"[tw] OR "margetuximab"[tw] OR "MGAH22"[tw] OR "tucatinib"[tw] OR "trastuzumab deruxtecan"[tw] OR "DS-8201a"[tw] OR "DS-8201"[tw]) AND ("Breast Neoplasms"[Mesh] OR "Breast Neoplasms"[tw] OR "Breast Neoplasm"[tw] OR "Breast Cancers"[tw] OR "Breast Cancer"[tw] OR "Breast Tumors"[tw] OR "Breast Tumor"[tw] OR "Breast Tumours"[tw] OR "Breast Tumour"[tw] OR "Breast Carcinomas"[tw] OR "Breast Carcinoma"[tw] OR "Breast Adenocarcinomas"[tw] OR "Breast Adenocarcinoma"[tw] OR "Breast Malignancies"[tw] OR "Breast Malignancy"[tw] OR "Cancer of Breast"[tw] OR "Cancer of the Breast"[tw] OR "Mammary Neoplasms"[tw] OR "Mammary Neoplasm"[tw] OR "Mammary Cancers"[tw] OR "Mammary Cancer"[tw] OR "Mammary Tumors"[tw] OR "Mammary Tumor"[tw] OR "Mammary Tumours"[tw] OR "Mammary Tumour"[tw] OR "Mammary Carcinomas"[tw] OR "Mammary Carcinoma"[tw] OR "Mammary Adenocarcinomas"[tw] OR "Mammary Adenocarcinoma"[tw] OR "Mammary Malignancies"[tw] OR "Mammary Malignancy"[tw]) AND ("HER2-positive"[tw] OR HER2 positiv*[tw] OR "HER-2-positive"[tw] OR HER 2 positiv*[tw] OR "human epidermal growth factor receptor 2-positive"[tw] OR "human epidermal growth factor receptor 2-positiv*"[tw] OR "human epidermal growth factor receptor2-positive"[tw] OR "human epidermal growth factor receptor2-positiv*"[tw] OR "human epidermal growth factor receptor-2-positive"[tw] OR "human epidermal growth factor receptor-2-positiv*"[tw] OR ERBB 2-positive [tw] OR ERBB 2-positiv* [tw] OR ERBB2-positive [tw] OR ERBB2-positiv* [tw] OR ERBB-2-positive [tw] OR ERBB-2-positiv* [tw] OR (("HER-2"[tw] OR "HER2"[tw] OR "HER 2"[tw] OR "human epidermal growth factor receptor-2"[tw] OR "human epidermal growth factor receptor2"[tw] OR "human epidermal growth factor receptor 2"[tw] OR "ERBB2"[tw] OR "ERBB-2"[tw] OR "ERBB 2"[tw]) AND ("amplified"[tw] OR "amplification"[tw] OR "gene amplification"[tw] OR "enriched"[tw] OR "over expression"[tw] OR "over expressing"[tw] OR "over expressed"[tw] OR "overexpression"[tw] OR "overexpressing"[tw] OR "overexpressed"[tw] OR "protein overexpression"[tw] OR "mutations"[tw] OR "mutant"[tw] OR "mutated"[tw]))) AND ("Neoplasm Metastasis"[mesh] OR "metastasis"[tw] OR "metastatic"[tw] OR metasta*[tw] OR "advanced"[tw])) AND ("Randomized Controlled Trial"[Publication Type] OR "Randomized Controlled Trials as Topic"[Mesh] OR "Randomized"[tw] OR "Randomised"[tw] OR "RCT"[tw] OR "RCTs"[tw] OR "control groups"[mesh] OR "control group"[tw] OR "control groups"[tw] OR "controlled clinical trial"[pt] OR "controlled clinical trials as topic"[mesh] OR "cross-over studies"[mesh] OR "cross over study"[tw] OR "cross over studies"[tw] OR "double-blind method"[mesh] OR "double blind"[tw] OR "placebos"[mesh] OR placebo*[tw] OR placebos*[tw] OR "RaCT"[tw] OR "RaCTs"[tw] OR "random allocation"[mesh] OR "Research Design"[MeSH:noexp] OR "Research design"[tw] OR "Research designs"[tw] OR "single blind"[tw] OR "single-blind method"[mesh] OR ((single*[tw] OR double*[tw] OR triple*[tw]) AND (blind*[tw] OR mask*[tw])) OR volunteer*[tw] OR "trial"[ti])

**Embase (1237)**

<http://ovidsp.ovid.com/ovidweb.cgi?T=JS&PAGE=main&MODE=ovid&D=oemezd>

(("Trastuzumab"/ OR "pertuzumab"/ OR "Ado-Trastuzumab Emtansine"/ OR "Lapatinib"/ OR "pyrotinib"/ OR "Afatinib"/ OR "neratinib"/ OR "margetuximab"/ OR "tucatinib"/ OR "trastuzumab deruxtecan"/ OR "trastuzumab".mp OR "Herceptin".mp OR "Trazimera".mp OR "pertuzumab".mp OR "Perjeta".mp OR "rhumab 2C4".mp OR "rhumab-2C4".mp OR "Omnitarg".mp OR "T-DM1".mp OR "TDM1".mp OR "Ado Trastuzumab Emtansine".mp OR "Trastuzumab Emtansine".mp OR "Kadcyla".mp OR "huN901-DM1".mp OR "huN901 DM1".mp OR "Trastuzumab-DM1 Conjugate".mp OR "Trastuzumab DM1 Conjugate".mp OR "Trastuzumab-DM1".mp OR "Trastuzumab DM1".mp OR "lapatinib".mp OR "Tykerb".mp OR "GW282974X".mp OR "GW572016".mp OR "GW-572016".mp OR "GW 572016".mp OR "pyrotinib".mp OR "afatinib".mp OR "Afatinib Maleate".mp OR "BIBW 2992".mp OR "BIBW2992".mp OR "BIBW-2992".mp OR "Gilotrif".mp OR "neratinib".mp OR "Nerlynx".mp OR "HKI 272".mp OR "HKI272".mp OR "HKI-272".mp OR "margetuximab".mp OR "MGAH22".mp OR "tucatinib".mp OR "trastuzumab deruxtecan".mp OR "DS-8201a".mp OR "DS-8201".mp) AND (exp "Breast Cancer"/ OR "Breast Neoplasms".mp OR "Breast Neoplasm".mp OR "Breast Cancers".mp OR "Breast Cancer".mp OR "Breast Tumors".mp OR "Breast Tumor".mp OR "Breast Tumours".mp OR "Breast Tumour".mp OR "Breast Carcinomas".mp OR "Breast Carcinoma".mp OR "Breast Adenocarcinomas".mp OR "Breast Adenocarcinoma".mp OR "Breast Malignancies".mp OR "Breast Malignancy".mp OR "Cancer of Breast".mp OR "Cancer of the Breast".mp OR "Mammary Neoplasms".mp OR "Mammary Neoplasm".mp OR "Mammary Cancers".mp OR "Mammary Cancer".mp OR "Mammary Tumors".mp OR "Mammary Tumor".mp OR "Mammary Tumours".mp OR "Mammary Tumour".mp OR "Mammary Carcinomas".mp OR "Mammary Carcinoma".mp OR "Mammary Adenocarcinomas".mp OR "Mammary Adenocarcinoma".mp OR "Mammary Malignancies".mp OR "Mammary Malignancy".mp) AND ("HER2-positive".mp OR HER2-positiv*.mp OR "HER-2-positive".mp OR HER2-positiv*.mp OR "human epidermal growth factor receptor 2-positive".mp OR "human epidermal growth factor receptor 2-positiv*".mp OR "human epidermal growth factor receptor2-positive".mp OR "human epidermal growth factor receptor2-positiv*".mp OR "human epidermal growth factor receptor-2-positive".mp OR "human epidermal growth factor receptor-2-positiv*".mp OR "ERBB2-positive".mp OR "ERBB2-positiv*".mp OR "ERBB-2-positive".mp OR "ERBB-2-positiv*".mp OR "ERBB 2-positive".mp OR "ERBB 2-positiv*".mp OR (("HER-2".mp OR "HER2".mp OR "HER 2".mp OR "human epidermal growth factor receptor-2".mp OR "human epidermal growth factor receptor2".mp OR "human epidermal growth factor receptor 2".mp OR "ERBB2".mp OR "ERBB-2".mp OR "ERBB 2".mp) AND ("amplified".mp OR "amplification".mp OR "gene amplification".mp OR "enriched".mp OR "over expression".mp OR "over expressing".mp OR "over expressed".mp OR "overexpression".mp OR "overexpressing".mp OR "overexpressed".mp OR "protein overexpression".mp OR "mutations".mp OR "mutant".mp OR "mutated".mp))) AND (exp "Metastasis"/ OR "metastasis".mp OR "metastatic".mp OR metasta*.mp OR "advanced".mp)) AND (exp "Randomized Controlled Trial"/ OR "randomized controlled trial (topic)"/ OR "Randomized".mp OR "Randomised".mp OR "RCT".mp OR "RCTs".mp OR "control group"/ OR "control group".mp OR "control groups".mp OR exp "controlled clinical trial"/ OR exp "controlled clinical trial (topic)"/ OR "crossover procedure"/ OR "cross over study".mp OR "cross over studies".mp OR "double blind procedure"/ OR "double blind".mp OR exp "placebo"/ OR placebo*.mp OR placebos*.mp OR "RaCT".mp OR "RaCTs".mp OR "randomization"/ OR "Research design".mp OR "Research designs".mp OR "single blind".mp OR "single blind procedure"/ OR ((single*.mp OR double*.mp OR triple*.mp) ADJ5 (blind*.mp OR mask*.mp)) OR volunteer*.mp OR "trial".ti) NOT (conference review or conference abstract).pt

**Cochrane library (801)**

<https://www.cochranelibrary.com/advanced-search/search-manager>

(("Trastuzumab" OR "pertuzumab" OR "Ado-Trastuzumab Emtansine" OR "Lapatinib" OR "pyrotinib" OR "Afatinib" OR "neratinib" OR "margetuximab" OR "tucatinib" OR "trastuzumab deruxtecan" OR "trastuzumab" OR "Herceptin" OR "Trazimera" OR "pertuzumab" OR "Perjeta" OR "rhumab 2C4" OR "rhumab-2C4" OR "Omnitarg" OR "T-DM1" OR "TDM1" OR "Ado Trastuzumab Emtansine" OR "Trastuzumab Emtansine" OR "Kadcyla" OR "huN901-DM1" OR "huN901 DM1" OR "Trastuzumab-DM1 Conjugate" OR "Trastuzumab DM1 Conjugate" OR "Trastuzumab-DM1" OR "Trastuzumab DM1" OR "lapatinib" OR "Tykerb" OR "GW282974X" OR "GW572016" OR "GW-572016" OR "GW 572016" OR "pyrotinib" OR "afatinib" OR "Afatinib Maleate" OR "BIBW 2992" OR "BIBW2992" OR "BIBW-2992" OR "Gilotrif" OR "neratinib" OR "Nerlynx" OR "HKI 272" OR "HKI272" OR "HKI-272" OR "margetuximab" OR "MGAH22" OR "tucatinib" OR "trastuzumab deruxtecan" OR "DS-8201a" OR "DS-8201") AND ("Breast Cancer" OR "Breast Neoplasms" OR "Breast Neoplasm" OR "Breast Cancers" OR "Breast Cancer" OR "Breast Tumors" OR "Breast Tumor" OR "Breast Tumours" OR "Breast Tumour" OR "Breast Carcinomas" OR "Breast Carcinoma" OR "Breast Adenocarcinomas" OR "Breast Adenocarcinoma" OR "Breast Malignancies" OR "Breast Malignancy" OR "Cancer of Breast" OR "Cancer of the Breast" OR "Mammary Neoplasms" OR "Mammary Neoplasm" OR "Mammary Cancers" OR "Mammary Cancer" OR "Mammary Tumors" OR "Mammary Tumor" OR "Mammary Tumours" OR "Mammary Tumour" OR "Mammary Carcinomas" OR "Mammary Carcinoma" OR "Mammary Adenocarcinomas" OR "Mammary Adenocarcinoma" OR "Mammary Malignancies" OR "Mammary Malignancy") AND ("HER2 positive" OR "HER 2 positive" OR "HER-2 positive" OR "human epidermal growth factor receptor 2-positive" OR "human epidermal growth factor receptor2-positive" OR "human epidermal growth factor receptor-2-positive" OR "ERBB2-positive" OR "ERBB-2-positive" OR "ERBB 2-positive" OR (("HER-2" OR "HER2" OR "HER 2" OR "human epidermal growth factor receptor-2" OR "human epidermal growth factor receptor2" OR "human epidermal growth factor receptor 2" OR "ERBB2" OR "ERBB-2" OR "ERBB 2") AND ("amplified" OR "amplification" OR "gene amplification" OR "enriched" OR "over expression" OR "over expressing" OR "over expressed" OR "overexpression" OR "overexpressing" OR "overexpressed" OR "protein overexpression" OR "mutations" OR "mutant" OR "mutated"))) AND ("Metastasis" OR "metastasis" OR "metastatic" OR metasta* OR "advanced")):ti,ab,kw NOT (conference abstract):pt
