# Supplementary material for: Efficacy and Safety of Anti-HER2 Agents in Combination With Chemotherapy for Metastatic HER2-Positive Breast Cancer Patient: A Network Meta-Analysis
Source: Front Oncol. 2021 Aug 19;11:731210. doi: 10.3389/fonc.2021.731210 (PMC8416996; doi:10.3389/fonc.2021.731210)
Supplement: Supplementary file 1 [file DataSheet_1.zip › Supplementary data 2 References of included and excluded studies.docx]

**Supplementary data 2 References of included and excluded studies**

**2A: References of included studies**

1. Andersson M, Lidbrink E, Bjerre K, et al. Phase III randomized study comparing docetaxel plus trastuzumab with vinorelbine plus trastuzumab as first-line therapy of metastatic or locally advanced human epidermal growth factor receptor 2-positive breast cancer: the HERNATA study. *J Clin Oncol.* Jan-20;29(3):264-271.

2. Awada A, Colomer R, Inoue K, et al. Neratinib Plus Paclitaxel vs Trastuzumab Plus Paclitaxel in Previously Untreated Metastatic ERBB2-Positive Breast Cancer: The NEfERT-T Randomized Clinical Trial. *JAMA Oncol.* Dec-1;2(12):1557-1564.

3. Baselga J, Cortés J, Kim SB, et al. Pertuzumab plus trastuzumab plus docetaxel for metastatic breast cancer. *N Engl J Med.* Jan-12 2012;366(2):109-119.

4. Baselga J, Manikhas A, Cortés J, et al. Phase III trial of nonpegylated liposomal doxorubicin in combination with trastuzumab and paclitaxel in HER2-positive metastatic breast cancer. *Annals of oncology : official journal of the european society for medical oncology.* 2014 2014;25(3):592‐598.

5. Burstein HJ, Keshaviah A, Baron AD, et al. Trastuzumab plus vinorelbine or taxane chemotherapy for HER2-overexpressing metastatic breast cancer: the trastuzumab and vinorelbine or taxane study. *Cancer.* Sep-1;110(5):965-972.

6. Diéras V, Miles D, Verma S, et al. Trastuzumab emtansine versus capecitabine plus lapatinib in patients with previously treated HER2-positive advanced breast cancer (EMILIA): a descriptive analysis of final overall survival results from a randomised, open-label, phase 3 trial. 2017(1474-5488 (Electronic)).

7. Gasparini G, Gion M, Mariani L, et al. Randomized Phase II Trial of weekly paclitaxel alone versus trastuzumab plus weekly paclitaxel as first-line therapy of patients with Her-2 positive advanced breast cancer. *Breast cancer research and treatment.* 2007 2007;101(3):355‐365.

8. Geyer CE, Forster J, Lindquist D, et al. Lapatinib plus capecitabine for HER2-positive advanced breast cancer. *New England journal of medicine.* 2006 2006;355(26):2733‐2743.

9. Gianni L, Romieu GH, Lichinitser M, et al. AVEREL: a randomized phase III Trial evaluating bevacizumab in combination with docetaxel and trastuzumab as first-line therapy for HER2-positive locally recurrent/metastatic breast cancer. *J Clin Oncol.* May-10;31(14):1719-1725.

10. Gómez HL, Neciosup S, Tosello C, et al. A Phase II Randomized Study of Lapatinib Combined With Capecitabine, Vinorelbine, or Gemcitabine in Patients With HER2-Positive Metastatic Breast Cancer With Progression After a Taxane (Latin American Cooperative Oncology Group 0801 Study). *Clinical breast cancer.* 2016 2016;16(1):38‐44.

11. Guan Z, Xu B, DeSilvio ML, et al. Randomized trial of lapatinib versus placebo added to paclitaxel in the treatment of human epidermal growth factor receptor 2-overexpressing metastatic breast cancer. *J Clin Oncol.* Jun-1;31(16):1947-1953.

12. Hamberg P, Bos MMEM, Braun HJJ, et al. Randomized phase II study comparing efficacy and safety of combination-therapy trastuzumab and docetaxel vs. sequential therapy of trastuzumab followed by docetaxel alone at progression as first-line chemotherapy in patients with HER2<sup>+</sup> metastatic breast cancer: Hertax trial. *Clinical Breast Cancer.* April;11(2):103-113.

13. Hurvitz. Phase II randomized study of trastuzumab emtansine versus trastuzumab plus docetaxel in patients with human epidermal growth factor receptor 2-positive metastatic breast cancer (Journal of Clinical Oncology (2013) 31 (1157-1163)). *Journal of Clinical Oncology.* 10-Aug 2013;31(23):2977.

14. Hurvitz SA, Andre F, Jiang Z, et al. Combination of everolimus with trastuzumab plus paclitaxel as first-line treatment for patients with HER2-positive advanced breast cancer (BOLERO-1): a phase 3, randomised, double-blind, multicentre trial. *Lancet Oncol.* Jul 2015;16(7):816-829.

15. Krop IE, Kim SB, González-Martín A, et al. Trastuzumab emtansine versus treatment of physician's choice for pretreated HER2-positive advanced breast cancer (TH3RESA): a randomised, open-label, phase 3 trial. 2014(1474-5488 (Electronic)).

16. Krop IE, Kim SB, Martin AG, et al. Trastuzumab emtansine versus treatment of physician's choice in patients with previously treated HER2-positive metastatic breast cancer (TH3RESA): final overall survival results from a randomised open-label phase 3 trial. 2017(1474-5488 (Electronic)).

17. Lin NU, Borges V, Anders C, et al. Intracranial Efficacy and Survival With Tucatinib Plus Trastuzumab and Capecitabine for Previously Treated HER2-Positive Breast Cancer With Brain Metastases in the HER2CLIMB Trial. *J Clin Oncol.* May-29:Jco2000775.

18. Lin NU, Eierman W, Greil R, et al. Randomized phase II study of lapatinib plus capecitabine or lapatinib plus topotecan for patients with HER2-positive breast cancer brain metastases. *J Neurooncol.* Dec;105(3):613-620.

19. Martin M, Bonneterre J, Geyer CE, Jr., et al. A phase two randomised trial of neratinib monotherapy versus lapatinib plus capecitabine combination therapy in patients with HER2+ advanced breast cancer. *Eur J Cancer.* Dec;49(18):3763-3772.

20. Marty M, Cognetti F, Maraninchi D, et al. Randomized phase II trial of the efficacy and safety of trastuzumab combined with docetaxel in patients with human epidermal growth factor receptor 2-positive metastatic breast cancer administered as first-line treatment: the M77001 study group. *Journal of clinical oncology.* 2005 2005;23(19):4265‐4274.

21. Murthy RK, Loi S, Okines A, et al. Tucatinib, Trastuzumab, and Capecitabine for HER2-Positive Metastatic Breast Cancer. *N Engl J Med.* Feb-13 2020;382(7):597-609.

22. Perez EA, Barrios C, Eiermann W, et al. Trastuzumab emtansine with or without pertuzumab versus trastuzumab plus taxane for human epidermal growth factor receptor 2-positive, advanced breast cancer: Primary results from the phase III MARIANNE study. *Journal of Clinical Oncology.* 10-Jan;35(2):141-148.

23. Perez EA, Barrios C, Eiermann W, et al. Trastuzumab emtansine with or without pertuzumab versus trastuzumab with taxane for human epidermal growth factor receptor 2-positive advanced breast cancer: Final results from MARIANNE. *Cancer.* Nov-15;125(22):3974-3984.

24. Robert N, Leyl, Jones B, et al. Randomized phase III study of trastuzumab, paclitaxel, and carboplatin compared with trastuzumab and paclitaxel in women with HER-2-overexpressing metastatic breast cancer. *Journal of clinical oncology.* 2006 2006;24(18):2786‐2792.

25. Swain SM, Baselga J, Kim SB, et al. Pertuzumab, trastuzumab, and docetaxel in HER2-positive metastatic breast cancer. *New England journal of medicine.* 2015 2015;372(8):724‐734.

26. Swain SM, Miles D, Kim SB, et al. Pertuzumab, trastuzumab, and docetaxel for HER2-positive metastatic breast cancer (CLEOPATRA): end-of-study results from a double-blind, randomised, placebo-controlled, phase 3 study. *The Lancet Oncology.* April 2020;21(4):519-530.

27. Takano T, Tsurutani J, Takahashi M, et al. A randomized phase II trial of trastuzumab plus capecitabine versus lapatinib plus capecitabine in patients with HER2-positive metastatic breast cancer previously treated with trastuzumab and taxanes: wJOG6110B/ELTOP. *Breast (edinburgh, scotland).* 2018 2018;40:67‐75.

28. Tolaney SM, Wardley AM, Zambelli S, et al. Abemaciclib plus trastuzumab with or without fulvestrant versus trastuzumab plus standard-of-care chemotherapy in women with hormone receptor-positive, HER2-positive advanced breast cancer (monarcHER): a randomised, open-label, phase 2 trial. *Lancet Oncol.* Apr-27.

29. Urruticoechea A, Rizwanullah M, Im SA, et al. Randomized phase III trial of trastuzumab plus capecitabine with or without pertuzumab in patients with human epidermal growth factor receptor 2-positive metastatic breast cancer who experienced disease progression during or after trastuzumab-based therapy. *Journal of Clinical Oncology.* 10-Sep;35(26):3030-3038.

30. Valero V, Forbes J, Pegram MD, et al. Multicenter phase III randomized trial comparing docetaxel and trastuzumab with docetaxel, carboplatin, and trastuzumab as first-line chemotherapy for patients with HER2-gene-amplified metastatic breast cancer (BCIRG 007 study): two highly active therapeutic regimens. *Journal of clinical oncology.* 2011 2011;29(2):149‐156.

31. Verma S, Miles D, Gianni L, et al. Trastuzumab emtansine for HER2-positive advanced breast cancer. *New England journal of medicine.* 2012 2012;367(19):1783‐1791.

32. von Minckwitz G, du Bois A, Schmidt M, et al. Trastuzumab beyond progression in human epidermal growth factor receptor 2-positive advanced breast cancer: a german breast group 26/breast international group 03-05 study. *Journal of clinical oncology.* 2009 2009;27(12):1999‐2006.

33. von Minckwitz G, Schwedler K, Schmidt M, et al. Trastuzumab beyond progression: overall survival analysis of the GBG 26/BIG 3-05 phase III study in HER2-positive breast cancer. *European journal of cancer (oxford, england : 1990).* 2011 2011;47(15):2273‐2281.

34. Wardley AM, Pivot X, Morales-Vasquez F, et al. Randomized phase II trial of first-line trastuzumab plus docetaxel and capecitabine compared with trastuzumab plus docetaxel in HER2-positive metastatic breast cancer. *Journal of clinical oncology.* 2010 2010;28(6):976‐983.

**2B: References of excluded studies**

- Reason for record exclusion: *non-RCTs;* N=38

[1. BOLERO-1: a randomized, phase III, double-blind, placebo-controlled multicenter trial of everolimus in combination with trastuzumab and paclitaxel as first-line therapy in women with HER2-positive (HER2+), locally advanced or metastatic breast cancer (BC).](http://www.who.int/trialsearch/Trial2.aspx?TrialID=EUCTR2011-003308-18-IT) *[Clinical advances in hematology & oncology.](http://www.who.int/trialsearch/Trial2.aspx?TrialID=EUCTR2011-003308-18-IT)* [2012 2012;10(9):14‐15.](http://www.who.int/trialsearch/Trial2.aspx?TrialID=EUCTR2011-003308-18-IT)

[2. Bachelot T, Garcia-Saenz JA, Verma S, et al. Sunitinib in combination with trastuzumab for the treatment of advanced breast cancer: activity and safety results from a phase II study.](http://www.who.int/trialsearch/Trial2.aspx?TrialID=EUCTR2011-003308-18-IT) *[BMC cancer.](http://www.who.int/trialsearch/Trial2.aspx?TrialID=EUCTR2011-003308-18-IT)* [2014 2014;14(1).](http://www.who.int/trialsearch/Trial2.aspx?TrialID=EUCTR2011-003308-18-IT)

[3. Blackwell KL, Pegram MD, Tan-Chiu E, et al. Single-agent lapatinib for HER2-overexpressing advanced or metastatic breast cancer that progressed on first- or second-line trastuzumab-containing regimens.](http://www.who.int/trialsearch/Trial2.aspx?TrialID=EUCTR2011-003308-18-IT) *[Annals of Oncology.](http://www.who.int/trialsearch/Trial2.aspx?TrialID=EUCTR2011-003308-18-IT)* [2009 2009;20(6):1026-1031.](http://www.who.int/trialsearch/Trial2.aspx?TrialID=EUCTR2011-003308-18-IT)

[4. Burstein HJ, Lieberman G, Slamon DJ, Winer EP, Klein P. Isolated central nervous system metastases in patients with HER2-overexpressing advanced breast cancer treated with first-line trastuzumab-based therapy.](http://www.who.int/trialsearch/Trial2.aspx?TrialID=EUCTR2011-003308-18-IT) *[Ann Oncol.](http://www.who.int/trialsearch/Trial2.aspx?TrialID=EUCTR2011-003308-18-IT)* [Nov;16(11):1772-1777.](http://www.who.int/trialsearch/Trial2.aspx?TrialID=EUCTR2011-003308-18-IT)

[5. Crown JP, Moulton B, O'Donovan N. A phase III randomized study of Paclitaxel and Trastuzumab versus Paclitaxel, Trastuzumab and Lapatinib in first line treatment of HER2 positive metastatic breast cancer 102.](http://www.who.int/trialsearch/Trial2.aspx?TrialID=EUCTR2011-003308-18-IT) *[Cancer research.](http://www.who.int/trialsearch/Trial2.aspx?TrialID=EUCTR2011-003308-18-IT)* [2012 2012;72(24):Abstract no: OT1‐1‐06.](http://www.who.int/trialsearch/Trial2.aspx?TrialID=EUCTR2011-003308-18-IT)

[6. Euctr IT. Randomized Phase II study of pertuzumab, trastuzumab and vinorelbine in patients with HER2-positive breast cancer.](http://www.who.int/trialsearch/Trial2.aspx?TrialID=EUCTR2011-003308-18-IT) *<http://www.who.int/trialsearch/Trial2.aspx?TrialID=EUCTR2011-003308-18-IT>.* 2012 2012.

7. Hurvitz SA, Dirix L, Kocsis J, et al. Phase II randomized study of trastuzumab emtansine versus trastuzumab plus docetaxel in patients with human epidermal growth factor receptor 2-positive metastatic breast cancer. *J Clin Oncol.* Mar-20;31(9):1157-1163.

8. Jackisch C. Treatment options in ErbB2 (HER2)-overexpressing breast cancer. *Breast Care.* May;3:1.

9. Jprn U. Multicenter pahse II tiral of combination therapy using abraxane and trastuzumab +-pertuzumab for HER2-positive locally advanced or metastatic breast cancer. [*http://www.who.int/trialsearch/Trial2.aspx?TrialID=JPRN-UMIN000006838*](http://www.who.int/trialsearch/Trial2.aspx?TrialID=JPRN-UMIN000006838)*.* 2011 2011.

10. Láng I, Bell R, Feng FY, et al. Trastuzumab retreatment after relapse on adjuvant trastuzumab therapy for human epidermal growth factor receptor 2-positive breast cancer: final results of the Retreatment after HErceptin Adjuvant trial. *Clin Oncol (R Coll Radiol).* Feb;26(2):81-89.

11. Liu S, Chen B, Burugu S, et al. Role of Cytotoxic Tumor-Infiltrating Lymphocytes in Predicting Outcomes in Metastatic HER2-Positive Breast Cancer: A Secondary Analysis of a Randomized Clinical Trial. *JAMA Oncology.* 2017;3(11):e172085-e172085.

12. McArthur HL, Morris PG, Hudis CA. Combination trastuzumab and chemotherapy may have a role in women with small, node-negative, human epidermal growth factor receptor 2-positive breast cancer. *Journal of Clinical Oncology.* 01-Jan;33(1):124-125.

13. Metzger O, Leone JP, Li T, et al. Phase I dose-escalation trial of tucatinib in combination with trastuzumab in patients with HER2-positive breast cancer brain metastases. *Ann Oncol.* May-24.

14. Moulder SL, Arteaga CL. A phase I/II trial of trastuzumab and gefitinib in patients with metastatic breast cancer that overexpresses HER2/neu (ErbB-2). *Clinical Breast Cancer.* June;4(2):142-145.

15. Nasrazadani A, Brufsky A. Neratinib: the emergence of a new player in the management of HER2+ breast cancer brain metastasis. *Future Oncol.* Mar;16(7):247-254.

16. Nct. ErbB2 Over-expressing Metastatic Breast Cancer Study Using Paclitaxel, Trastuzumab, and Lapatinib. *https://clinicaltrials.gov/show/NCT00272987.* 2006 2006.

17. Pegram MD, Pienkowski T, Northfelt DW, et al. Results of two open-label, multicenter phase II studies of docetaxel, platinum salts, and trastuzumab in HER2-positive advanced breast cancer. *J Natl Cancer Inst.* May-19;96(10):759-769.

18. Perez EA, Suman VJ, Rowl, et al. Two concurrent phast II trials of paclitaxel/carboplatin/trastuzumab (weekly of every-3-week schedule) as first-line therapy in women with HER2-overexpressing metastatic breast cancer: NCCTG study 983252. *Clinical breast cancer.* 2005 2005;6(5):425‐432.

19. Poncet B, Bachelot T, Colin C, et al. Use of the monoclonal antibody anti-HER2 trastuzumab in the treatment of metastatic breast cancer a cost-effectiveness analysis. *American Journal of Clinical Oncology: Cancer Clinical Trials.* August;31(4):363-368.

20. Ricci F, Tourneau CL. Trastuzumab emtansine in HER2-positive metastatic breast cancer: What is the best sequence? *Chinese Clinical Oncology.* 2018 2018;7(1).

21. Rugo H, Brammer M, Zhang F, Lalla D. Effect of trastuzumab on health-related quality of life in patients with HER2-positive metastatic breast cancer: Data from three clinical trials. *Clinical Breast Cancer.* 01-Aug;10(4):288-293.

22. Rugo HS. Highlights from the 2019 San Antonio breast cancer symposium. *Clinical Advances in Hematology and Oncology.* March;18(3):157-159.

23. Rugo HS, Barve A, Waller CF, et al. Effect of a proposed trastuzumab biosimilar compared with trastuzumab on overall response rate in patients with ERBB2 (HER2)-positive metastatic breast cancer: A randomized clinical trial. *JAMA - Journal of the American Medical Association.* 2017 2017;317(1):37-47.

24. Ryan Q, Ibrahim A, Cohen MH, et al. FDA drug approval summary: Lapatinib in combination with capecitabine for previously treated metastatic breast cancer that overexpresses HER-2. *Journal of Immunology.* 15-May;192(10):1114-1119.

25. Sayan M, Abou Yehia Z, Gupta A, Toppmeyer D, Ohri N, Haffty BG. Acute Cardiotoxicity With Concurrent Trastuzumab and Hypofractionated Radiation Therapy in Breast Cancer Patients. *Frontiers in Oncology.* 01-Oct;9.

26. Schramm A, Friedl TW, Schochter F, et al. Therapeutic intervention based on circulating tumor cell phenotype in metastatic breast cancer: concept of the DETECT study program. *Archives of gynecology and obstetrics.* 2016 2016;293(2):271‐281.

27. Sweetlove M. Phase III trial of lapatinib: Discontinued due to inferior efficacy. *Pharmaceutical Medicine.* 2012 2012;26(5):321-325.

28. Tamura K, Tsurutani J, Takahashi S, et al. Trastuzumab deruxtecan (DS-8201a) in patients with advanced HER2-positive breast cancer previously treated with trastuzumab emtansine: a dose-expansion, phase 1 study. *The lancet. Oncology.* 2019 2019.

29. Untch M, Jackisch C. Therapy with lapatinib: Current status in women with advanced breast cancer. *Breast Care.* April;2(2):76-80.

30. Untch M, Muscholl M, Tjul, et al. First-line trastuzumab plus epirubicin and cyclophosphamide therapy in patients with human epidermal growth factor receptor 2-positive metastatic breast cancer: cardiac safety and efficacy data from the Herceptin, Cyclophosphamide, and Epirubicin (HERCULES) trial. *Journal of clinical oncology.* 2010 2010;28(9):1473‐1480.

31. Villanueva MT. TH3RESA trial, overcoming hurdles in breast cancer. *Nature Reviews Clinical Oncology.* July;11(7):379.

32. Walshe JM, Denduluri N, Berman AW, Rosing DR, Swain SM. A phase II trial with trastuzumab and pertuzumab in patients with HER2-overexpressed locally advanced and metastatic breast cancer. *Clin Breast Cancer.* Feb;6(6):535-539.

33. Xu B, Kim SB, Inoue K, et al. Neratinib-based therapy in patients with metastatic HER2-positive breast cancer from Asia. *Future Oncology.* 2019 2019;15(28):3243-3253.

34. Yamamoto Y, Iwata H, Ueno T, et al. A randomized, open-label, Phase III trial of pertuzumab retreatment in HER2-positive locally advanced/metastatic breast cancer patients previously treated with pertuzumab, trastuzumab and chemotherapy: the Japan Breast Cancer Research Group-M05 PRECIOUS study. *Jpn J Clin Oncol.* Sep-1;48(9):855-859.

35. Yamamura J, Kamigaki S, Hamakawa T, et al. Efficacy and safety of pertuzumab for HER2-positive metastatic breast cancer. [Japanese]. *Japanese Journal of Cancer and Chemotherapy.* 01-Jun;42(6):713-717.

36. Yaqub F. T-DM1 for HER2-positive metastatic breast cancer. *Lancet Oncol.* Mar;14(3):e94.

37. Yardley DA, Daniel D, Stipanov M, et al. A phase II trial of oxaliplatin and trastuzumab in the treatment of HER2-positive metastatic breast cancer. *Cancer Invest.* Oct;28(8):865-871.

38. Zhao M, Pan X, Layman R, et al. A Phase II study of bevacizumab in combination with trastuzumab and docetaxel in HER2 positive metastatic breast cancer. *Investigational new drugs.* 2014 2014;32(6):1285‐1294.

- Reason for record exclusion: *ineligible patients;* N=13

[1. Euctr AT. A randomised, multicentre, double-blind, placebo-controlled, 2-arm, Phase III study of oral GW572016 in combination with paclitaxel in subjects previously untreated for advanced or metastatic breast cancer. *http://www.who.int/trialsearch/Trial2.aspx?TrialID=EUCTR2004-001999-40-AT*](http://www.who.int/trialsearch/Trial2.aspx?TrialID=EUCTR2004-001999-40-AT)*.* 2004 2004.

2. Fabi A, Ferretti G, Salesi N, et al. Can HER2 overexpression predict response to pegylated liposomal doxorubicin in metastatic breast cancer patients? [4]. *Annals of Oncology.* March;16(3):516-517.

3. Fountzilas G, Dafni U, Dimopoulos MA, et al. A randomized phase III study comparing three anthracycline-free taxane-based regimens, as first line chemotherapy, in metastatic breast cancer: a Hellenic Cooperative Oncology Group study. *Breast cancer research and treatment.* 2009 2009;115(1):87‐99.

4. Irct201208116302N. Comparative analysis of trastuzumab(Aryogen) with Herceptin®. [*http://www.who.int/trialsearch/Trial2.aspx?TrialID=IRCT201208116302N4*](http://www.who.int/trialsearch/Trial2.aspx?TrialID=IRCT201208116302N4)*.* 2013 2013.

5. Joensuu H, Bono P, Kataja V, et al. Fluorouracil, epirubicin, and cyclophosphamide with either docetaxel or vinorelbine, with or without trastuzumab, as adjuvant treatments of breast cancer: final results of the FinHer Trial. *Journal of clinical oncology.* 2009 2009;27(34):5685‐5692.

6. Joensuu H, Kellokumpu-Lehtinen PL, Bono P, et al. Adjuvant docetaxel or vinorelbine with or without trastuzumab for breast cancer. *New England Journal of Medicine.* 23-Feb;354(8):809-820.

7. Melcher CA, Janni JW, Schneeweiss A, Fasching PA, Hagenbeck CD, Aktas B. DETECT III - A multicenter, randomized, phase III study to compare standard therapy alone versus standard therapy plus lapatinib in patients with initially HER2-negative metastatic breast cancer but with HER2-positive circulating tumorcells 101. *Cancer research.* 2012 2012;72(24):Abstract no: OT1‐1‐10.

8. Nct. Paclitaxel With or Without Trastuzumab in Treating Patients With or Without HER-2/Neu Breast Cancer That is Inoperable, Recurrent, or Metastatic. *https://clinicaltrials.gov/show/NCT00003440.* 1999 1999.

9. Nct. Gemcitabine/ Trastuzumab and Gemcitabine/ Cisplatin/ Trastuzumab in Patients With Metastatic Breast Cancer. *https://clinicaltrials.gov/show/NCT00201760.* 2005 2005.

10. Nct. Carboplatin or Docetaxel in Treating Women With Metastatic Genetic Breast Cancer. *https://clinicaltrials.gov/show/NCT00321633.* 2006 2006.

11. Nct. Continued HER2 Suppression With Lapatinib Plus Trastuzumab Versus Trastuzumab Alone. *https://clinicaltrials.gov/show/NCT00968968.* 2009 2009.

12. Spector NL, Xia W, Burris H, et al. Study of the biologic effects of lapatinib, a reversible inhibitor of ErbB1 and ErbB2 tyrosine kinases, on tumor growth and survival pathways in patients with advanced malignancies. *Journal of clinical oncology.* 2005 2005;23(11):2502‐2512.

13. Tan-Chiu E, Yothers G, Romond E, et al. Assessment of cardiac dysfunction in a randomized trial comparing doxorubicin and cyclophosphamide followed by paclitaxel, with or without trastuzumab as adjuvant therapy in node-positive, human epidermal growth factor receptor 2-overexpressing breast cancer: NSABP B-31. *J Clin Oncol.* Nov-1;23(31):7811-7819.

- Reason for record exclusion: *ineligible interventions;* N=37

[1. André F, O'Regan R, Ozguroglu M, et al. Everolimus for women with trastuzumab-resistant, HER2-positive, advanced breast cancer (BOLERO-3): a randomised, double-blind, placebo-controlled phase 3 trial.](http://www.who.int/trialsearch/Trial2.aspx?TrialID=EUCTR2015-004189-27-ES) *[Lancet Oncol.](http://www.who.int/trialsearch/Trial2.aspx?TrialID=EUCTR2015-004189-27-ES)* [May;15(6):580-591.](http://www.who.int/trialsearch/Trial2.aspx?TrialID=EUCTR2015-004189-27-ES)

[2. Apsangikar P, Chaudhry S, Naik M, Deoghare S, Joseph J. A comparative phase III clinical study to evaluate efficacy and safety of TrastuRel™ (biosimilar trastuzumab) and innovator trastuzumab in patients with metastatic human epidermal growth factor receptor 2 (HER2)-overexpressing breast cancer.](http://www.who.int/trialsearch/Trial2.aspx?TrialID=EUCTR2015-004189-27-ES) *[Indian J Cancer.](http://www.who.int/trialsearch/Trial2.aspx?TrialID=EUCTR2015-004189-27-ES)* [Oct-Dec;54(4):664-668.](http://www.who.int/trialsearch/Trial2.aspx?TrialID=EUCTR2015-004189-27-ES)

[3. Bischoff J, Barinoff J, Mundhenke C, et al. A randomized phase II study to determine the efficacy and tolerability of two doses of eribulin plus lapatinib in trastuzumab-pretreated patients with HER-2-positive metastatic breast cancer (E-VITA).](http://www.who.int/trialsearch/Trial2.aspx?TrialID=EUCTR2015-004189-27-ES) *[Anticancer Drugs.](http://www.who.int/trialsearch/Trial2.aspx?TrialID=EUCTR2015-004189-27-ES)* [Apr;30(4):394-401.](http://www.who.int/trialsearch/Trial2.aspx?TrialID=EUCTR2015-004189-27-ES)

[4. Blackwell KL, Burstein HJ, Storniolo AM, et al. Randomized study of Lapatinib alone or in combination with trastuzumab in women with ErbB2-positive, trastuzumab-refractory metastatic breast cancer.](http://www.who.int/trialsearch/Trial2.aspx?TrialID=EUCTR2015-004189-27-ES) *[J Clin Oncol.](http://www.who.int/trialsearch/Trial2.aspx?TrialID=EUCTR2015-004189-27-ES)* [Mar-1;28(7):1124-1130.](http://www.who.int/trialsearch/Trial2.aspx?TrialID=EUCTR2015-004189-27-ES)

[5. Blackwell KL, Burstein HJ, Storniolo AM, et al. Overall survival benefit with lapatinib in combination with trastuzumab for patients with human epidermal growth factor receptor 2-positive metastatic breast cancer: final results from the EGF104900 Study.](http://www.who.int/trialsearch/Trial2.aspx?TrialID=EUCTR2015-004189-27-ES) *[J Clin Oncol.](http://www.who.int/trialsearch/Trial2.aspx?TrialID=EUCTR2015-004189-27-ES)* [Jul-20;30(21):2585-2592.](http://www.who.int/trialsearch/Trial2.aspx?TrialID=EUCTR2015-004189-27-ES)

[6. Cortés J, Dieras V, Ro J, et al. Afatinib alone or afatinib plus vinorelbine versus investigator's choice of treatment for HER2-positive breast cancer with progressive brain metastases after trastuzumab, lapatinib, or both (LUX-Breast 3): a randomised, open-label, multicentre, phase 2 trial.](http://www.who.int/trialsearch/Trial2.aspx?TrialID=EUCTR2015-004189-27-ES) *[The lancet. Oncology.](http://www.who.int/trialsearch/Trial2.aspx?TrialID=EUCTR2015-004189-27-ES)* [2015 2015;16(16):1700‐1710.](http://www.who.int/trialsearch/Trial2.aspx?TrialID=EUCTR2015-004189-27-ES)

[7. Drooger JC, van Tinteren H, de Groot SM, et al. A randomized phase 2 study exploring the role of bevacizumab and a chemotherapy-free approach in HER2-positive metastatic breast cancer: the HAT study (BOOG 2008-2003), a Dutch Breast Cancer Research Group trial.](http://www.who.int/trialsearch/Trial2.aspx?TrialID=EUCTR2015-004189-27-ES) *[Cancer.](http://www.who.int/trialsearch/Trial2.aspx?TrialID=EUCTR2015-004189-27-ES)* [2016 2016.](http://www.who.int/trialsearch/Trial2.aspx?TrialID=EUCTR2015-004189-27-ES)

[8. Euctr ES. A Study to Evaluate the Efficacy and Safety of Trastuzumab Emtansine in Combination With Atezolizumab or Atezolizumab-Placebo in Participants With HER2-Positive Locally Advanced or Metastatic Breast Cancer who Have Received Prior Trastuzumab and Taxane Based Therapy.](http://www.who.int/trialsearch/Trial2.aspx?TrialID=EUCTR2015-004189-27-ES) *<http://www.who.int/trialsearch/Trial2.aspx?TrialID=EUCTR2015-004189-27-ES>.* 2016 2016.

9. Euctr IT. A randomized Phase III, double-blind, placebo-controlled multicenter trial of daily everolimus in combination with trastuzumab and vinorelbine, in pretreated women with HER2/neu over-expressing locally advanced or metastatic breast cancer. - BOLERO-3. [*http://www.who.int/trialsearch/Trial2.aspx?TrialID=EUCTR2008-008697-31-IT*](http://www.who.int/trialsearch/Trial2.aspx?TrialID=EUCTR2008-008697-31-IT)*.* 2009 2009.

10. Gelmon KA, Boyle FM, Kaufman B, et al. Lapatinib or Trastuzumab Plus Taxane Therapy for Human Epidermal Growth Factor Receptor 2-Positive Advanced Breast Cancer: final Results of NCIC CTG MA.31. *Journal of clinical oncology.* 2015 2015;33(14):1574‐1583.

11. Gomez HL, Doval DC, Chavez MA, et al. Efficacy and safety of lapatinib as first-line therapy for ErbB2-amplified locally advanced or metastatic breast cancer. *Journal of Clinical Oncology.* 2008 2008;26(18):2999-3005.

12. Huober J, Fasching PA, Barsoum M, et al. Higher efficacy of letrozole in combination with trastuzumab compared to letrozole monotherapy as first-line treatment in patients with HER2-positive, hormone-receptor-positive metastatic breast cancer - results of the eLEcTRA trial. *Breast (edinburgh, scotland).* 2012 2012;21(1):27‐33.

13. Inoue K, Nakagami K, Mizutani M, et al. Randomized phase III trial of trastuzumab monotherapy followed by trastuzumab plus docetaxel versus trastuzumab plus docetaxel as first-line therapy in patients with HER2-positive metastatic breast cancer: the JO17360 Trial Group. *Breast cancer research and treatment.* 2010 2010;119(1):127‐136.

14. Janni W, Sarosiek T, Karaszewska B, et al. A phase II, randomized, multicenter study evaluating the combination of lapatinib and vinorelbine in women with ErbB2 overexpressing metastatic breast cancer. *Breast Cancer Res Treat.* Feb;143(3):493-505.

15. Janni W, Sarosiek T, Karaszewska B, et al. Final overall survival analysis of a phase II trial evaluating vinorelbine and lapatinib in women with ErbB2 overexpressing metastatic breast cancer. *Breast (edinburgh, scotland).* 2015 2015;24(6):769‐773.

16. Johnston S, Pippen J, Pivot X, et al. Lapatinib combined with letrozole versus letrozole and placebo as first-line therapy for postmenopausal hormone receptor-positive metastatic breast cancer. *Journal of clinical oncology.* 2009 2009;27(33):5538‐5546.

17. Johnston SR, Gómez H, Stemmer SM, et al. A randomized and open-label trial evaluating the addition of pazopanib to lapatinib as first-line therapy in patients with HER2-positive advanced breast cancer. *Breast Cancer Res Treat.* Feb;137(3):755-766.

18. Kaufman B, Mackey JR, Clemens MR, et al. Trastuzumab plus anastrozole versus anastrozole alone for the treatment of postmenopausal women with human epidermal growth factor receptor 2-positive, hormone receptor-positive metastatic breast cancer: results from the randomized phase III TAnDEM study. *J Clin Oncol.* Nov-20;27(33):5529-5537.

19. Krop IE, Modi S, LoRusso PM, et al. Phase 1b/2a study of trastuzumab emtansine (T-DM1), paclitaxel, and pertuzumab in HER2-positive metastatic breast cancer. *Breast Cancer Res.* Mar-15;18(1):34.

20. Ma F, Ouyang Q, Li W, et al. Pyrotinib or Lapatinib Combined With Capecitabine in HER2-Positive Metastatic Breast Cancer With Prior Taxanes, Anthracyclines, and/or Trastuzumab: a Randomized, Phase II Study. *Journal of clinical oncology.* 2019 2019;37(29):2610‐2619.

21. Nct. A Study to Evaluate the Efficacy and Safety of Herceptin® (Trastuzumab) in Combination With Arimidex® (Anastrozole) an Aromatase Inhibitor Compared to Arimidex® Alone in Patients With Metastatic Breast Cancer. *https://clinicaltrials.gov/show/NCT00022672.* 2001 2001.

22. Nct. Lapatinib In Combination With Trastuzumab Versus Lapatinib Monotherapy In Subjects With HER2-positive Metastatic Breast Cancer. *https://clinicaltrials.gov/show/NCT00320385.* 2006 2006.

23. Nct. A Study of Herceptin (Trastuzumab) in Combination With 2nd-Line Chemotherapy in Patients With HER2 Positive Metastatic Breast Cancer. *https://clinicaltrials.gov/show/NCT00444587.* 2007 2007.

24. Nct. Lapatinib Plus Capecitabine Versus Trastuzumab Plus Capecitabine in ErbB2 (HER2) Positive Metastatic Breast Cancer. *https://clinicaltrials.gov/show/NCT00820222.* 2009 2009.

25. Nct. Lux-Breast 3; Afatinib Alone or in Combination With Vinorelbine in Patients With Human Epidermal Growth Factor Receptor 2 (HER2) Positive Breast Cancer Suffering From Brain Metastases. *https://clinicaltrials.gov/show/NCT01441596.* 2011 2011.

26. Nct. A Study of Pertuzumab in Combination With Trastuzumab Plus an Aromatase Inhibitor in Participants With Metastatic Human Epidermal Growth Factor Receptor 2 (HER2)-Positive and Hormone Receptor-Positive Advanced Breast Cancer. *https://clinicaltrials.gov/show/NCT01491737.* 2011 2011.

27. Pagani O, Klingbiel D, Ruhstaller T, et al. Do all patients with advanced HER2 positive breast cancer need upfront-chemo when receiving trastuzumab? Randomized phase III trial SAKK 22/99. *Annals of oncology : official journal of the european society for medical oncology.* 2017 2017;28(2):305‐312.

28. Pivot X, Manikhas A, Żurawski B, et al. CEREBEL (EGF111438): A Phase III, Randomized, Open-Label Study of Lapatinib Plus Capecitabine Versus Trastuzumab Plus Capecitabine in Patients With Human Epidermal Growth Factor Receptor 2-Positive Metastatic Breast Cancer. *J Clin Oncol.* May-10;33(14):1564-1573.

29. Ranganathan A, Moore Z, O'Shaughnessy JA. Trastuzumab prolongs progression-free survival in patients with hormone-sensitive and HER2-overexpressing metastatic breast cancer. *Clinical Breast Cancer.* February;7(6):450-451.

30. Rimawi M, Ferrero JM, de la Haba-Rodriguez J, et al. First-Line Trastuzumab Plus an Aromatase Inhibitor, With or Without Pertuzumab, in Human Epidermal Growth Factor Receptor 2-Positive and Hormone Receptor-Positive Metastatic or Locally Advanced Breast Cancer (PERTAIN): A Randomized, Open-Label Phase II Trial. *J Clin Oncol.* Oct-1;36(28):2826-2835.

31. Slamon DJ, Leyl, Jones B, et al. Use of chemotherapy plus a monoclonal antibody against HER2 for metastatic breast cancer that overexpresses HER2. *N Engl J Med.* Mar-15;344(11):783-792.

32. Tryfonidis K, Marreaud S, Khaled H, et al. Cardiac safety, efficacy, and correlation of serial serum HER2-extracellular domain shed antigen measurement with the outcome of the combined trastuzumab plus CMF in women with HER2-positive metastatic breast cancer: results from the EORTC 10995 phase II study. *Breast cancer research and treatment.* 2017 2017;163(3):507‐515.

33. Vogel CL, Cobleigh MA, Tripathy D, et al. First-line Herceptin monotherapy in metastatic breast cancer. *Oncology.* 2001 2001;61:37‐42.

34. Vogel CL, Cobleigh MA, Tripathy D, et al. Efficacy and safety of trastuzumab as a single agent in first-line treatment of HER2-overexpressing metastatic breast cancer. *Journal of clinical oncology.* 2002 2002;20(3):719‐726.

35. Wildiers H, Tryfonidis K, Dal Lago L, et al. Pertuzumab and trastuzumab with or without metronomic chemotherapy for older patients with HER2-positive metastatic breast cancer (EORTC 75111-10114): an open-label, randomised, phase 2 trial from the Elderly Task Force/Breast Cancer Group. *Lancet Oncol.* Mar;19(3):323-336.

36. Harbeck N, Huang CS, Hurvitz S, et al. Afatinib plus vinorelbine versus trastuzumab plus vinorelbine in patients with HER2-overexpressing metastatic breast cancer who had progressed on one previous trastuzumab treatment (LUX-Breast 1): an open-label, randomised, phase 3 trial. *Lancet Oncol.* Mar;17(3):357-366.

37. Pegram MD, Bondarenko I, Zorzetto MMC, et al. PF-05280014 (a trastuzumab biosimilar) plus paclitaxel compared with reference trastuzumab plus paclitaxel for HER2-positive metastatic breast cancer: a randomised, double-blind study. *British journal of cancer.* 2019 2019;120(2):172‐182.

- Reason for record exclusion: *non-English/Chinese;* N=1

1. Schwartz GN. Effects of G-CSF, Trastuzumab, and Vinorelbine on Immune Cell Function in Patients With Her-2 Positive Metastatic Breast Cancer. Physician data query (PDQ). 2006 2006.

- Reason for record exclusion: *not available in full text;* N=24

1. Baselga J, Swain SM. CLEOPATRA: a phase III evaluation of pertuzumab and trastuzumab for HER2-positive metastatic breast cancer. *Clin Breast Cancer.* Dec-1;10(6):489-491.

2. Burstein HJ, Barry WT, Cirrincione C, Chew HK, Tolaney S, Lake D. Abstract PD05-01: CALGB 40302: fulvestrant with or without Lapatinib as Therapy for Hormone Receptor Positive Advanced Breast Cancer: a Double-Blinded, Placebo-Controlled, Randomized Phase III Study. 2010 2010;70.

3. Castan JC, Verma S, Hurvitz S, et al. HERMIONE: A phase 2, randomized, open label trial comparing MM-302 plus trastuzumab with chemotherapy of physician's choice plus trastuzumab, in anthracycline naive HER2-positive, locally advanced/metastatic breast cancer patients previously treated with pertuzumab and T-DM1. *Annals of Oncology.* 1-October;27:vi99.

4. Extra JM, Cognetti F, Chan S, et al. Randomised phase II trial (M77001) of trastuzumab (Herceptin) plus docetaxel versus docetaxel alone, as first-line therapy in patients with HER2-positive metastatic breast cancer. *European journal of cancer.* 2003 2003;1(5):S202.

5. Forbes JF, Pienkowski T, Valero V, et al. BCIRG 007: randomized phase III trial of trastuzumab plus docetaxel with or without carboplatin first line in HER2 positive metastatic breast cancer (MBC). *Journal of clinical oncology: ASCO annual meeting proceedings.* 2006 2006;24:LBA516.

6. Guan Z, Xu B, Arpornwirat W, Tong Z, Lorvidhaya V, Wang L. Abstract P3-14-24: overall Survival Benefit Observed with Lapatinib (L) Plus Paclitaxel (P) as First-Line Therapy in Patients with HER2-Overexpressing Metastatic Breast Cancer (MBC). 2010 2010;70.

7. Hamberg P, Bontenbal M, Vernhout RM, Bos MM, Braun HJ, ErdkampF. Combined trastuzumab (HER)/docetaxel (TAX) versus sequential trastuzumab followed by docetaxel at progression as first line chemotherapy for Her2-positive metastatic breast cancer: preliminary results (multicenter BOOGstudy; 2002-02). 2007 2007.

8. Huober J, Fasching P, Paepke S, Kubista E, Barsoum M, Wallwiener D. Letrozole in Combination with Trastuzumab Is Superior to Letrozole Monotherapy as First Line Treatment in Patients with Hormone-Receptor-Positive, HER2- Positive Metastatic Breast Cancer (MBC) - Results of the eLEcTRA Trial. 2010 2010;69(24).

9. Huober J, Ribi K, Weder P, et al. Pertuzumab (P) + trastuzumab (T) with or without chemotherapy both followed by T-DM1 in case of progression in patients with HER2-positive metastatic breast cancer (MBC) - The PERNETTA trial (SAKK 22/10), a randomized open label phase II study (SAKK, UNICANCER, BOOG). *Annals of oncology : official journal of the European Society for Medical Oncology.* 01-May;30:iii47.

10. Joensuu H, Ould-Kaci M. LUX-Breast 3: randomized Phase II trial of afatinib (BIBW 2992) alone or with vinorelbine versus investigator's choice of treatment in patients (pts) with HER2-positive breast cancer (BC) with progressive brain metastases after trastuzumab and/or lapatinib-based therapy* 98. *Cancer research.* 2012 2012;72(24):Abstract no: OT1‐1‐15.

11. Koeberle D, Ruhstaller T, Jost L, Pagani O, Zaman K, von Moos R. Abstract P3-14-16: can Resistance to Trastuzumab Be Reversed by Endocrine Therapy? Results from a Proof-of-Principle Trial in Postmenopausal Patients with Hormonal Receptor (HR) Positive, HER-2 Positive Advanced Breast Cancer (SAKK 23/03). 2010 2010;70.

12. LeoAdi, GomezH, AzizZ, ZvirbuleZ, ArbushitesM, OlivaCr. Lapatinib (L) with paclitaxel compared to paclitaxel as first-line treatment for patients with metastatic breast cancer: a phase III randomized, double-blind study of 580 patients. *Proceedings of the american society of clinical oncology.* 2007 2007;25(18).

13. Lin N, Danso M, David A, Muscato J, Ellis C, DeSilvio M. HALT MBC: hER2 Suppression with the Addition of Lapatinib to Trastuzumab in HER2-Positive Metastatic Breast Cancer (LPT112515) 92. *Cancer research.* 2011 2011;71(24):Abstract no: OT1‐02‐02.

14. Lin N, Danso MA, David AK, Muscato J, Rayson D, Houck WA. Human epidermal growth factor receptor 2 (HER2) suppression with the addition of lapatinib to trastuzumab in HER2-positive metastatic breast cancer (LTP112515) 103. *Cancer research.* 2012 2012;72(24):Abstract no: OT1‐1‐07.

15. Miles D, Baselga J, Amadori D, Sunpaweravong P, Semiglazov V, Knott A. Pertuzumab (P) in combination with trastuzumab (T) and docetaxel (D) in elderly patients with HER2-positive metastatic breast cancer in the CLEOPATRA study 84. *Cancer research.* 2012 2012;72(24):Abstract no: P5‐18‐01.

16. Naughton MJ, Gu L, Wang XF, Seidman AD. Quality of life (QOL) companion to CALGB 9840: a phase III study of paclitaxel (P) via weekly 1 hour (hr) versus standard 3 hour infusion every 3 weeks with trastuzumab in the treatment of patients with/without HER-2/neu-overexpressing metastatic breast cancer. *Journal of clinical oncology.* 2006 2006;24(18):46s.

17. Osoba D, Slamon D, Burchmore M, Murphy M. Health-related quality of life (HRQL) in women with HER2-positive metastatic breast cancer: effect of treatment with trastuzumab (Herceptin) plus chemotherapy versus chemotherapy alone. *European journal of cancer.* 2001 2001;37:S189.

18. Robert NJ, Leyl, B. J, et al. Randomized phase III trial study of trastuzumab, paclitaxel, and carboplatin versus trastuzumab and paclitaxel in women with HER-2 overexpressing metastatic breast cancer: an update including survival. *Annual meeting proceedings of the american society of clinical oncology.* 2004 2004:20.

19. Robert NJ, Slamon D, Leyl, et al. Toxicity profiles: a comparitive study of Herceptin (trastuzumab) and Taxol (paclitaxel) versus Herceptin, Taxol and carboplatin in HER-2 positive patients with advanced breast cancer. *Breast cancer research and treatment.* 2001 2001;69(3):304.

20. RobertNj, Leyl, Jones B, et al. Randomized phase III study of trastuzumab, paclitaxel, and carboplatin versus trastuzumab and paclitaxel in women with HER-2 overexpressing metastatic breast cancer: an update including survival. *Proceedings of the american society of clinical oncology.* 2004 2004;22(14).

21. Slamon D, Gomez HL, Kabbinavar FF, et al. Randomized study of pazopanib + lapatinib vs. lapatinib alone in patients with HER2-positive advanced or metastatic breast cancer. *Journal of clinical oncology: ASCO annual meeting proceedings.* 2008 2008;26:45.

22. Swain SM, Kim SB, Cortes J, Ro J, Semiglazov V, Campone M. Confirmatory overall survival (OS) analysis of CLEOPATRA: a randomized, double-blind, placebo-controlled Phase III study with pertuzumab (P), trastuzumab (T), and docetaxel (D) in patients (pts) with HER2-positive first-line (1L) metastatic breast cancer (MBC) 87. *Cancer research.* 2012 2012;72(24):Abstract no: P5‐18‐26.

23. von MG, Zielinski C, Maarteense E, et al. Capecitabine vs. capecitabine + trastuzumab in patients with HER2-positive metastatic breast cancer progressing during trastuzumab treatment: the TBP phase III study (GBG 26/BIG 3 05). *Journal of clinical oncology: ASCO annual meeting proceedings.* 2008 2008;26:47.

24. Yamashita T, Masuda N, Saji S, et al. Trastuzumab, pertuzumab, and eribulin mesylate versus trastuzumab, pertuzumab, and a taxane as a first-line or second-line treatment for HER2-positive, locally advanced or metastatic breast cancer: study protocol for a randomized controlled, non-inferiority, phase III trial in Japan (JBCRG-M06/EMERALD). *Trials.* May-7;21(1):391.

- Reason for record exclusion: *ineligible outcomes;* N=36

[1. Baselga J, Kim SB, Im SA, Hegg R, Im YH, Roman L. A Phase III, Randomized, Double-Blind, Placebo-Controlled Registration Trial To Evaluate the Efficacy and Safety of Pertuzumab + Trastuzumab + Docetaxel vs. Placebo + Trastuzumab + Docetaxel in Patients with Previously Untreated HER2-Positive Metastatic Breast Cancer (CLEOPATRA) 14.](http://www.who.int/trialsearch/Trial2.aspx?TrialID=CTRI/2010/091/001181) *[Cancer research.](http://www.who.int/trialsearch/Trial2.aspx?TrialID=CTRI/2010/091/001181)* [2011 2011;71(24):Abstract no: S5‐5.](http://www.who.int/trialsearch/Trial2.aspx?TrialID=CTRI/2010/091/001181)

[2. Ctri. A clinical trial to study the effect and safety of CT-P6 as compared to Herceptin in patients with metastatic breast cancer.](http://www.who.int/trialsearch/Trial2.aspx?TrialID=CTRI/2010/091/001181) *<http://www.who.int/trialsearch/Trial2.aspx?TrialID=CTRI/2010/091/001181>.* 2010 2010.

3. Ctri. Phase III clinical trial comparing safety and efficacy of BCD-022 (CJSC BIOCAD, Russia) used with paclitaxel to HerceptinÂ® used with paclitaxel in the first-line treatment of HER2 positive metastatic breast cancer patients. [*http://www.who.int/trialsearch/Trial2.aspx?TrialID=CTRI/2014/07/004722*](http://www.who.int/trialsearch/Trial2.aspx?TrialID=CTRI/2014/07/004722)*.* 2014 2014.

4. Euctr FR. A randomized, 3 arm, multicentre, phase III study to evaluate the efficacy and the safety of T-DM1 combined with pertuzumab or T-DM1 combined with pertuzumab-placebo (blinded for pertuzumab), versus the combination of trastuzumab plus taxane, as first line treatment in HER2- positive progressive or recurrent locally advanced or metastatic breast cancer (MBC). [*http://www.who.int/trialsearch/Trial2.aspx?TrialID=EUCTR2009-017905-13-FR*](http://www.who.int/trialsearch/Trial2.aspx?TrialID=EUCTR2009-017905-13-FR)*.* 2010 2010.

5. Euctr SI. A randomized, multicenter, phase III open-label study of the efficacy and safety of trastuzumab-MCC-DM1 vs. capecitabine + lapatinib in patients with HER2-positive locally advanced or metastatic breast cancer who have received prior trastuzumab-based therapy - EMILIA. [*http://www.who.int/trialsearch/Trial2.aspx?TrialID=EUCTR2008-005713-22-SI*](http://www.who.int/trialsearch/Trial2.aspx?TrialID=EUCTR2008-005713-22-SI)*.* 2009 2009.

6. Isrctn. Lapatinib plus capecitabine versus continued trastuzumab plus capecitabine after local therapy in patients with ErbB2-positive metastatic breast cancer developing brain metastasis/es. [*http://www.who.int/trialsearch/Trial2.aspx?TrialID=ISRCTN58771616*](http://www.who.int/trialsearch/Trial2.aspx?TrialID=ISRCTN58771616)*.* 2011 2011.

7. Li C, Wang B, Chen SC, et al. Exposure-response analyses of trastuzumab emtansine in patients with HER2-positive advanced breast cancer previously treated with trastuzumab and a taxane. *Cancer Chemotherapy and Pharmacology.* 01-Dec;80(6):1079-1090.

8. Miles D, Baselga J, Amadori D, et al. Treatment of older patients with HER2-positive metastatic breast cancer with pertuzumab, trastuzumab, and docetaxel: subgroup analyses from a randomized, double-blind, placebo-controlled phase III trial (CLEOPATRA). *Breast Cancer Res Treat.* Nov;142(1):89-99.

9. Nct. A Study of Avastin (Bevacizumab) in Combination With Herceptin (Trastuzumab)/Docetaxel in Patients With HER2 Positive Metastatic Breast Cancer. *https://clinicaltrials.gov/show/NCT00391092.* 2006 2006.

10. Nct. ALTTO (Adjuvant Lapatinib And/Or Trastuzumab Treatment Optimisation) Study; BIG 2-06/N063D. *https://clinicaltrials.gov/show/NCT00490139.* 2007 2007.

11. Nct. A Study to Evaluate Pertuzumab + Trastuzumab + Docetaxel vs. Placebo + Trastuzumab + Docetaxel in Previously Untreated HER2-Positive Metastatic Breast Cancer. *https://clinicaltrials.gov/show/NCT00567190.* 2007 2007.

12. Nct. Study Evaluating Neratinib Versus Lapatinib Plus Capecitabine For ErbB2 Positive Advanced Breast Cancer. *https://clinicaltrials.gov/show/NCT00777101.* 2008 2008.

13. Nct. A Study of the Efficacy and Safety of Trastuzumab Emtansine (Trastuzumab-MCC-DM1) vs. Trastuzumab (Herceptin®) and Docetaxel (Taxotere®) in Patients With Metastatic HER2-positive Breast Cancer Who Have Not Received Prior Chemotherapy for Metastatic Disease. *https://clinicaltrials.gov/show/NCT00679341.* 2008 2008.

14. Nct. Everolimus in Combination With Trastuzumab and Paclitaxel in the Treatment of HER2 Positive Locally Advanced or Metastatic Breast Cancer. *https://clinicaltrials.gov/show/NCT00876395.* 2009 2009.

15. Nct. Study Evaluating Neratinib Plus Paclitaxel VS Trastuzumab Plus Paclitaxel In ErbB-2 Positive Advanced Breast Cancer. *https://clinicaltrials.gov/show/NCT00915018.* 2009 2009.

16. Nct. A Study of Trastuzumab Emtansine Versus Capecitabine + Lapatinib in Participants With HER2-positive Locally Advanced or Metastatic Breast Cancer. *https://clinicaltrials.gov/show/NCT00829166.* 2009 2009.

17. Nct. A Study of Trastuzumab Emtansine (T-DM1) Plus Pertuzumab/Pertuzumab Placebo Versus Trastuzumab [Herceptin] Plus a Taxane in Participants With Metastatic Breast Cancer (MARIANNE). *https://clinicaltrials.gov/show/NCT01120184.* 2010 2010.

18. Nct. A Phase Ib/II Study of BEZ235 and Trastuzumab in Patients With HER2-positive Breast Cancer Who Failed Prior to Trastuzumab. *https://clinicaltrials.gov/show/NCT01471847.* 2011 2011.

19. Nct. A Study of Trastuzumab Emtansine in Comparison With Treatment of Physician's Choice in Participants With HER2-positive Breast Cancer Who Have Received at Least Two Prior Regimens of HER2-directed Therapy. *https://clinicaltrials.gov/show/NCT01419197.* 2011 2011.

20. Nct. A Safety and Efficacy Study of BCD-022 With Paclitaxel Compared to Herceptin With Paclitaxel in HER2-Positive Metastatic Breast Cancer Patients. *https://clinicaltrials.gov/show/NCT01764022.* 2013 2013.

21. Nct. A Study Of PF-05280014 [Trastuzumab-Pfizer] Or Herceptin® [Trastuzumab-EU] Plus Paclitaxel In HER2 Positive First Line Metastatic Breast Cancer Treatment (REFLECTIONS B327-02). *https://clinicaltrials.gov/show/NCT01989676.* 2013 2013.

22. Nct. A Study to Evaluate the Efficacy and Safety of Trastuzumab Emtansine Versus the Combination of Trastuzumab Plus Docetaxel in Patients With HER2-positive Breast Cancer. *https://clinicaltrials.gov/show/NCT02144012.* 2014 2014.

23. Nct. A Study to Evaluate the Efficacy and Safety of Pertuzumab + Trastuzumab + Docetaxel Versus Placebo + Trastuzumab + Docetaxel in Previously Untreated Human Epidermal Growth Factor Receptor 2 (HER2)-Positive Metastatic Breast Cancer (MBC). *https://clinicaltrials.gov/show/NCT02896855.* 2016 2016.

24. Osoba D, Slamon DJ, Burchmore M, Murphy M. Effects on quality of life of combined trastuzumab and chemotherapy in women with metastatic breast cancer. *J Clin Oncol.* Jul-15;20(14):3106-3113.

25. Per. A PHASE III TRAIL TO COMPARE THE SAFETY AND EFFICACY OF LAPATINIB PLUS TRASTUZUMAB PLUS AN AROMATASE INHIBITOR (AI) VERSUS TRASTUZUMAB PLUS AN AI VERSUS LAPATINIB PLUS AN AI AS FIRST-LINE THERAPY IN POSTMENOPAUSAL SUBJECT WITH HORMONE RECEPTOR POSITIVE, HER2-POSITIVE METASTATIC BREAST CANCER (MBC) WHO HAVE RECEIVED TRASTUZUMAB AND ENDOCRINE THERAPY IN THE NEOADJUVANT AND/OR ADJUVANT SETTING. [*http://www.who.int/trialsearch/Trial2.aspx?TrialID=PER-126-11*](http://www.who.int/trialsearch/Trial2.aspx?TrialID=PER-126-11)*.* 2012 2012.

26. Per. A PHASE 3 RANDOMIZED, DOUBLE-BLIND STUDY OF PF-05280014 PLUS PACLITAXEL VERSUS TRASTUZUMAB PLUS PACLITAXEL FOR THE FIRST-LINE TREATMENT OF PATIENTS WITH HER2-POSITIVE METASTATIC BREAST CANCER. [*http://www.who.int/trialsearch/Trial2.aspx?TrialID=PER-010-14*](http://www.who.int/trialsearch/Trial2.aspx?TrialID=PER-010-14)*.* 2014 2014.

27. Swain SM, Baselga J, Miles D, et al. Incidence of central nervous system metastases in patients with HER2-positive metastatic breast cancer treated with pertuzumab, trastuzumab, and docetaxel: results from the randomized phase III study CLEOPATRA. *Ann Oncol.* Jun;25(6):1116-1121.

28. Swain SM, Ewer MS, Cortés J, et al. Cardiac tolerability of pertuzumab plus trastuzumab plus docetaxel in patients with HER2-positive metastatic breast cancer in CLEOPATRA: a randomized, double-blind, placebo-controlled phase III study. *Oncologist.* 2013 2013;18(3):257-264.

29. Swain SM, Im YH, Im SA, et al. Safety profile of pertuzumab with trastuzumab and docetaxel in patients from asia with human epidermal growth factor receptor 2-positive metastatic breast cancer: Results from the phase III trial CLEOPATRA. *Oncologist.* 2014 2014;19(7):693-701.

30. Swain SM, Kim SB, Cortés J, et al. Pertuzumab, trastuzumab, and docetaxel for HER2-positive metastatic breast cancer (CLEOPATRA study): overall survival results from a randomised, double-blind, placebo-controlled, phase 3 study. *The lancet. Oncology.* 2013 2013;14(6):461‐471.

31. Toi M, Shao Z, Hurvitz S, et al. Efficacy and safety of everolimus in combination with trastuzumab and paclitaxel in Asian patients with HER2+ advanced breast cancer in BOLERO-1. *Breast Cancer Res.* Apr-11;19(1):47.

32. Tripathy D, Seidman A, Keefe D, Hudis C, Paton V, Lieberman G. Effect of cardiac dysfunction of treatment outcomes in women receiving trastuzumab for HER2-overexpressing metastatic breast cancer. *Clinical Breast Cancer.* October;5(4):293-298.

33. Wang J, Song P, Schrieber S, et al. Exposure-response relationship of T-DM1: insight into dose optimization for patients with HER2-positive metastatic breast cancer. *Clinical pharmacology and therapeutics.* 2014 2014;95(5):558‐564.

34. Wardley A, Ant¢n-Torres A, Pivot X, Morales-Vasquez F, Zetina L, DiasGaui M. Evaluation of trastuzumab, docetaxel and capecitabine as.. rst-line therapy for HER2-positive locally advanced or metastatic breast cancer. 2007 2007.

35. Welslau M, Diéras V, Sohn JH, et al. Patient-reported outcomes from EMILIA, a randomized phase 3 study of trastuzumab emtansine (T-DM1) versus capecitabine and lapatinib in human epidermal growth factor receptor 2-positive locally advanced or metastatic breast cancer. *Cancer.* Mar-1;120(5):642-651.

36. Wu Y, Amonkar MM, Sherrill BH, et al. Impact of lapatinib plus trastuzumab versus single-agent lapatinib on quality of life of patients with trastuzumab-refractory HER2+ metastatic breast cancer. *Ann Oncol.* Dec;22(12):2582-2590.
