# Supplementary material for: Efficacy and Safety of Anti-HER2 Agents in Combination With Chemotherapy for Metastatic HER2-Positive Breast Cancer Patient: A Network Meta-Analysis
Source: Front Oncol. 2021 Aug 19;11:731210. doi: 10.3389/fonc.2021.731210 (PMC8416996; doi:10.3389/fonc.2021.731210)
Supplement: Supplementary file 1 [file DataSheet_1.zip › Supplementary data 4A Trace plot and density plot of PFS in first line studies.pdf]

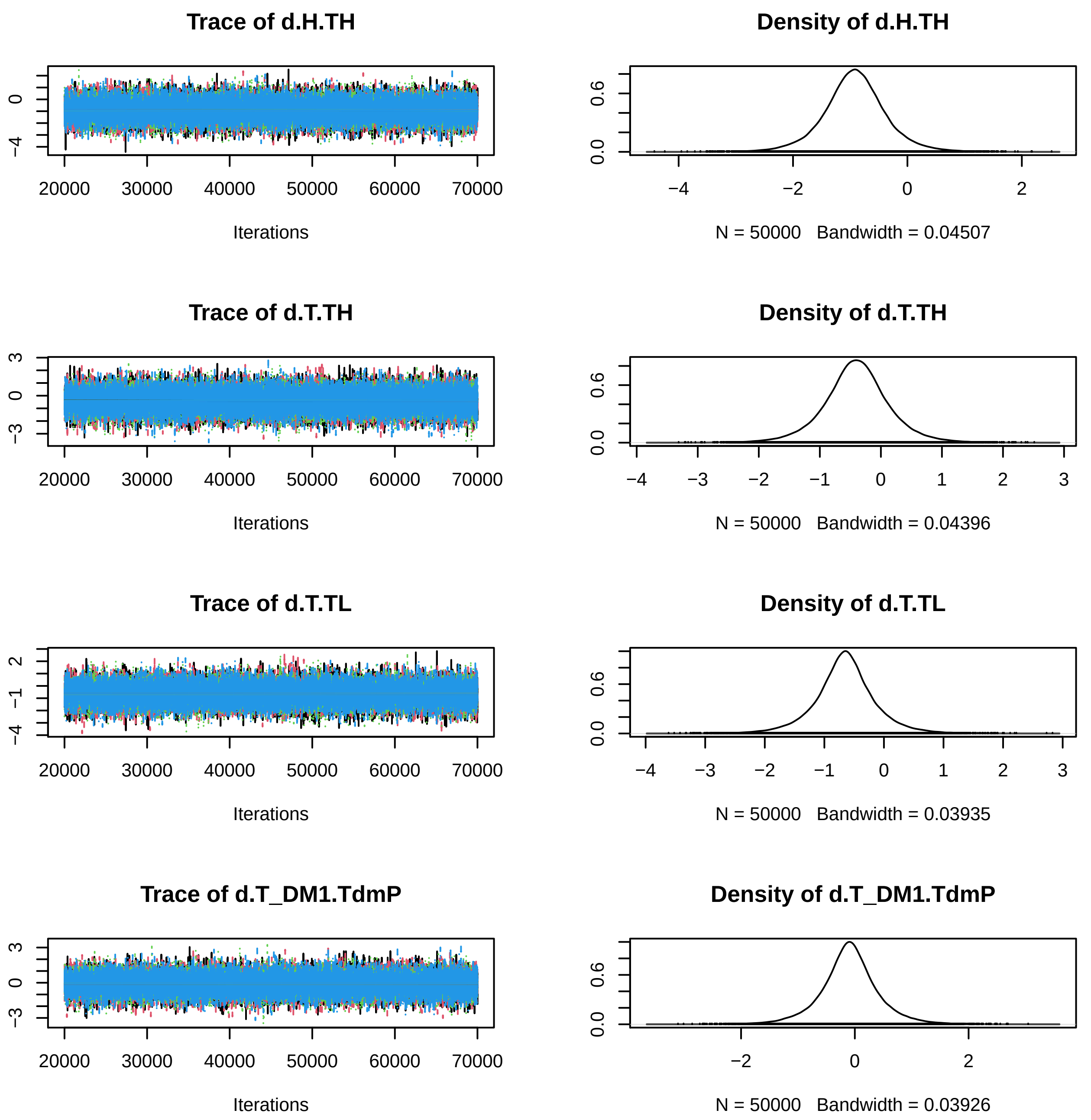
Trace of d.H.TH
Iterations
Density of d.H.TH
N = 50000 Bandwidth = 0.04507
Trace of d.T.TH
Iterations
Density of d.T.TH
N = 50000 Bandwidth = 0.04396
Trace of d.T.TL
Iterations
Density of d.T.TL
N = 50000 Bandwidth = 0.03935
Trace of d.T_DM1.TdmP
Iterations
Density of d.T_DM1.TdmP
N = 50000 Bandwidth = 0.03926
20000
30000
40000
50000
60000
70000

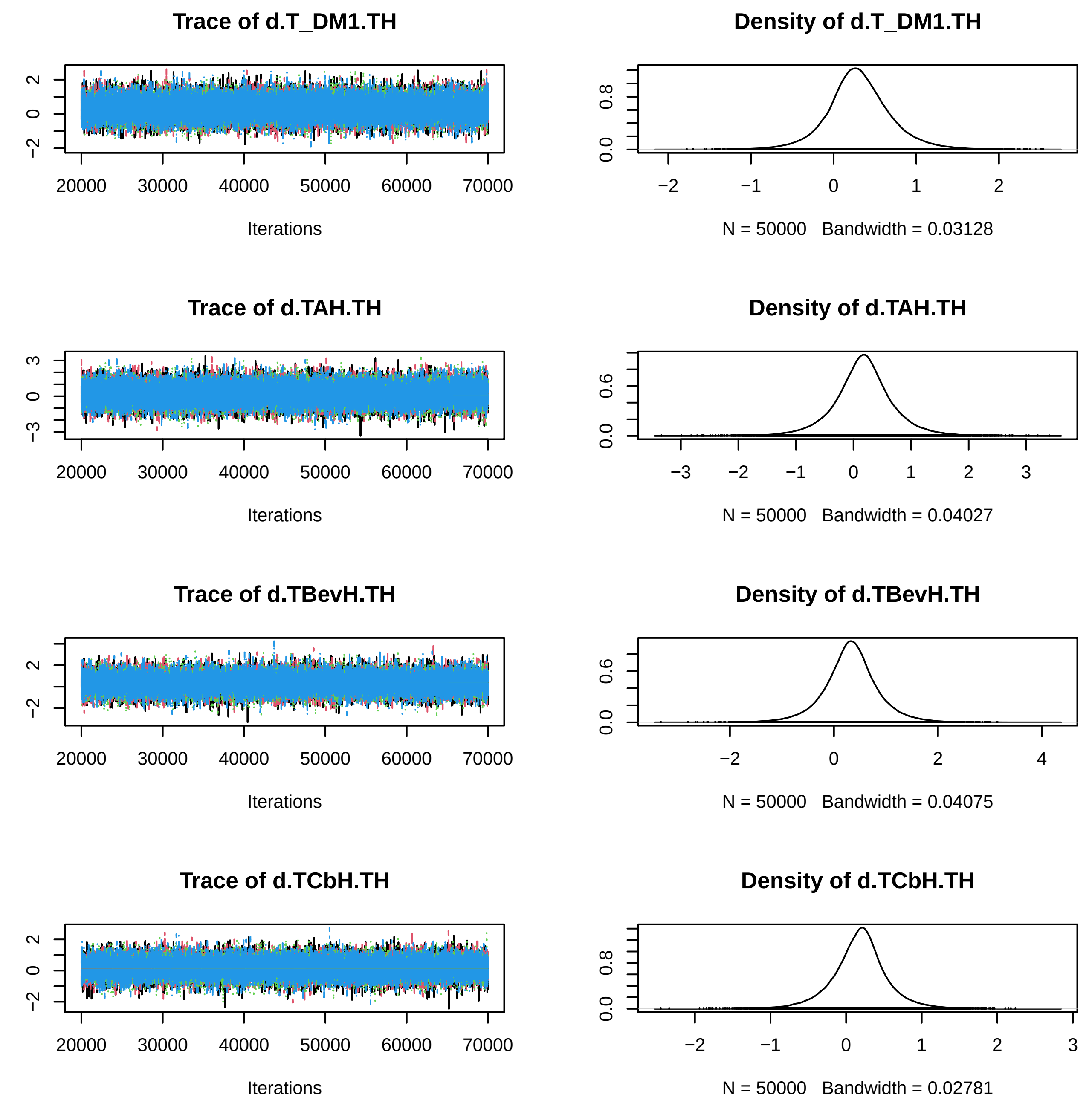
Trace of d.T_DM1.TH
Iterations
Density of d.T_DM1.TH
N = 50000 Bandwidth = 0.03128
Trace of d.TAH.TH
Iterations
Density of d.TAH.TH
N = 50000 Bandwidth = 0.04027
Trace of d.TBevH.TH
Iterations
Density of d.TBevH.TH
N = 50000 Bandwidth = 0.04075
Trace of d.TCbH.TH
Iterations
Density of d.TCbH.TH
N = 50000 Bandwidth = 0.02781

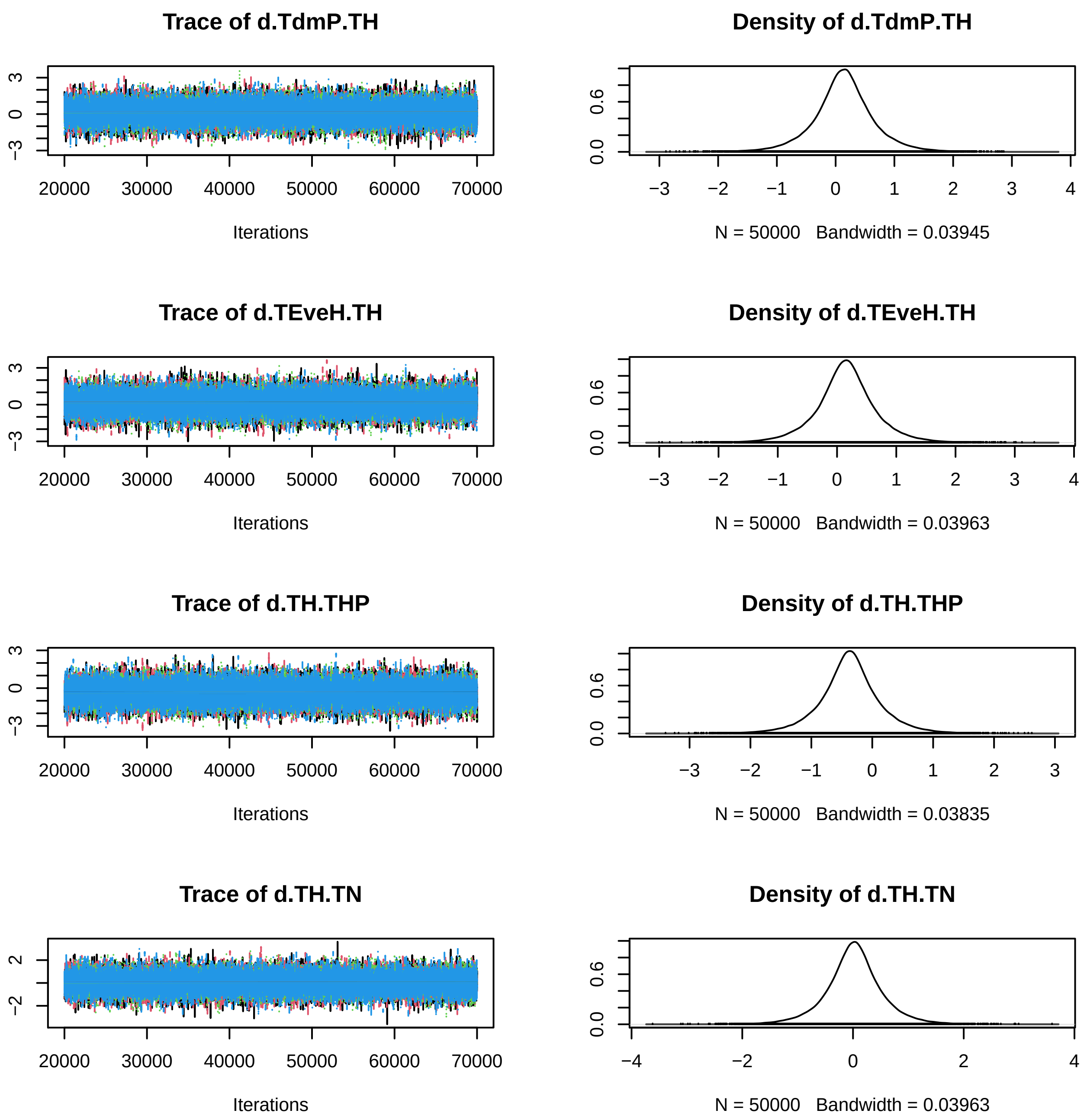
Trace of d.TdmP.TH
Density of d.TdmP.TH
N = 50000 Bandwidth = 0.03945
Trace of d.TEveH.TH
Density of d.TEveH.TH
N = 50000 Bandwidth = 0.03963
Trace of d.TH.THP
Density of d.TH.THP
N = 50000 Bandwidth = 0.03835
Trace of d.TH.TN
Density of d.TH.TN
N = 50000 Bandwidth = 0.03963
Iterations
20000
30000
40000
50000
60000
70000

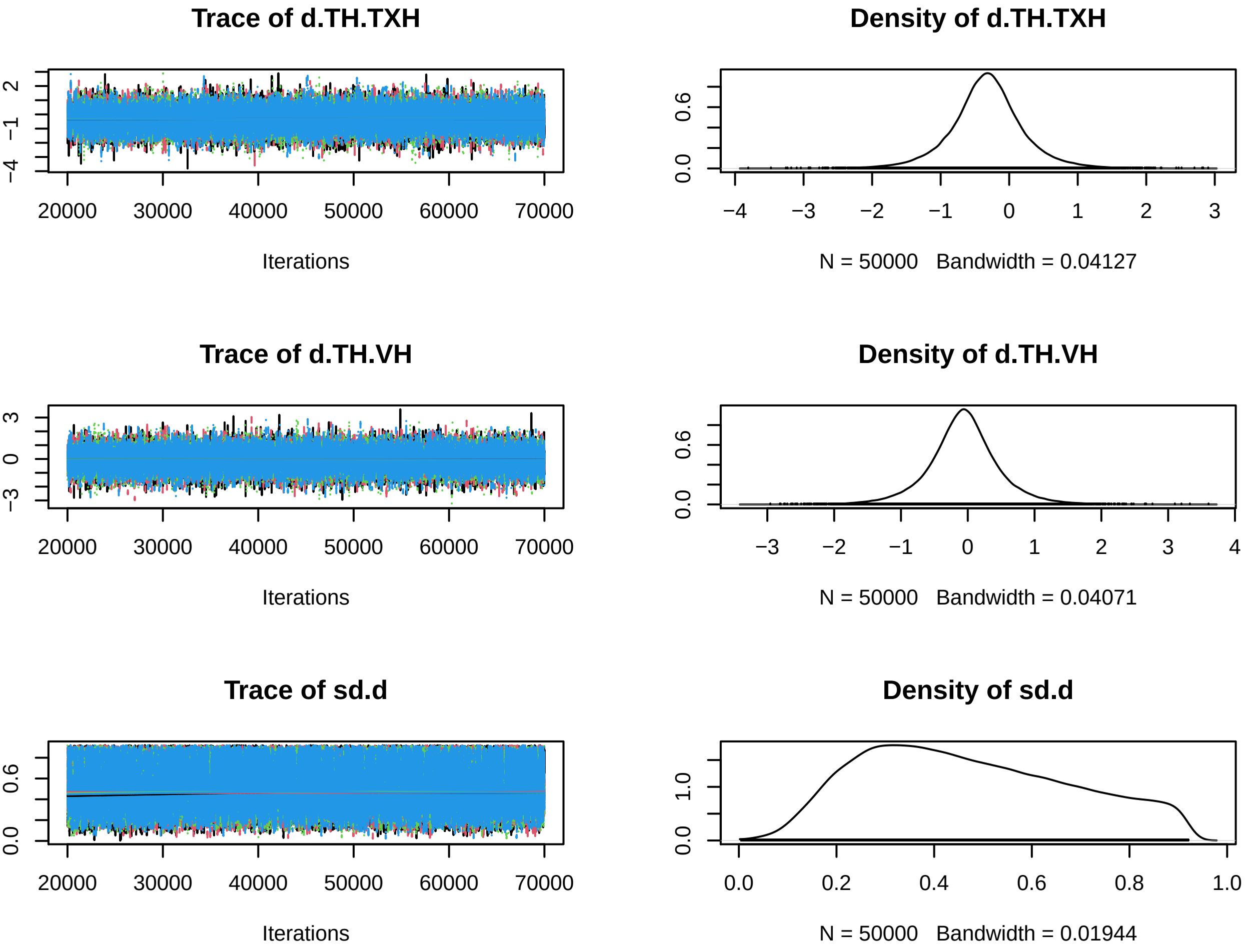
Trace of d.TH.TXH
Iterations
Density of d.TH.TXH
N = 50000 Bandwidth = 0.04127
Trace of d.TH.VH
Iterations
Density of d.TH.VH
N = 50000 Bandwidth = 0.04071
Trace of sd.d
Iterations
Density of sd.d
N = 50000 Bandwidth = 0.01944
