# Supplementary figures and images for: Efficacy and Safety of Anti-HER2 Agents in Combination With Chemotherapy for Metastatic HER2-Positive Breast Cancer Patient: A Network Meta-Analysis
Source: Front Oncol. 2021 Aug 19;11:731210. doi: 10.3389/fonc.2021.731210 (PMC8416996; doi:10.3389/fonc.2021.731210)

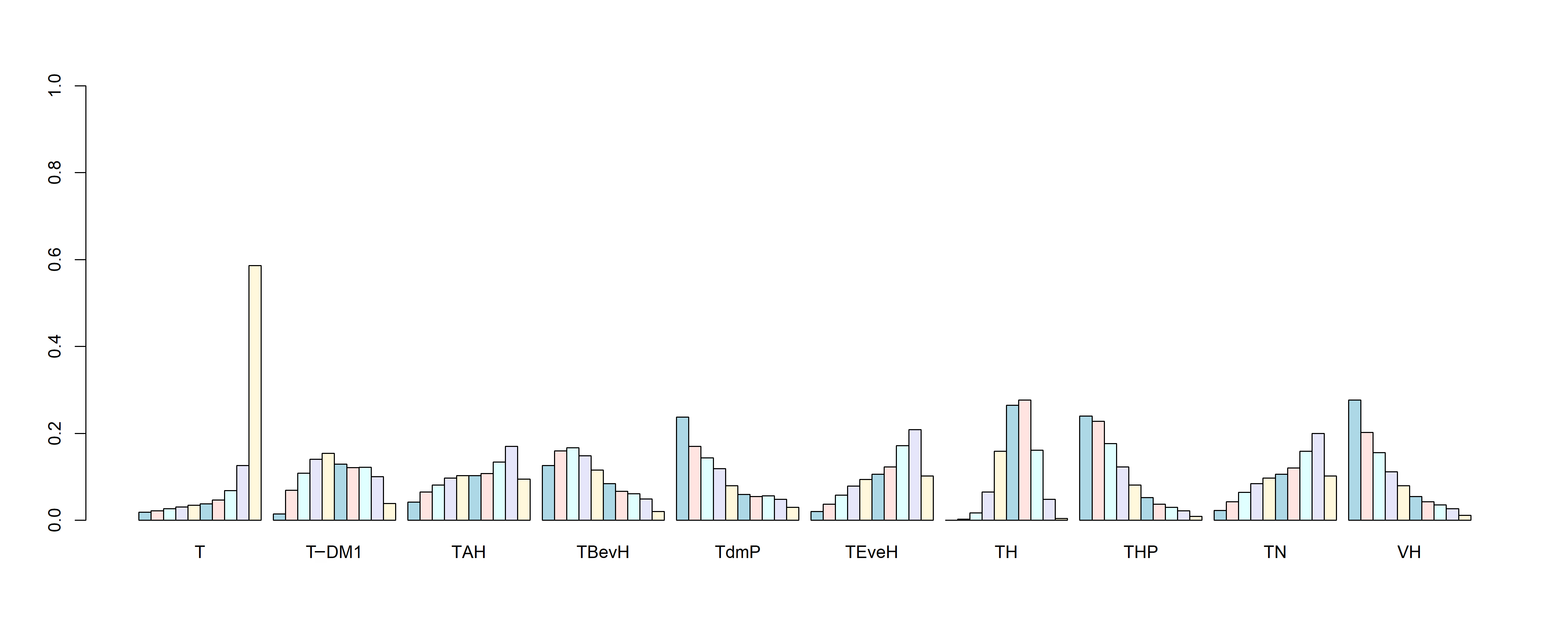

Supplement: Supplementary file 1 [file DataSheet_1.zip › Supplementary data 5D-1 Ranking histogram for PFS in first line HR+ studies.tiff]

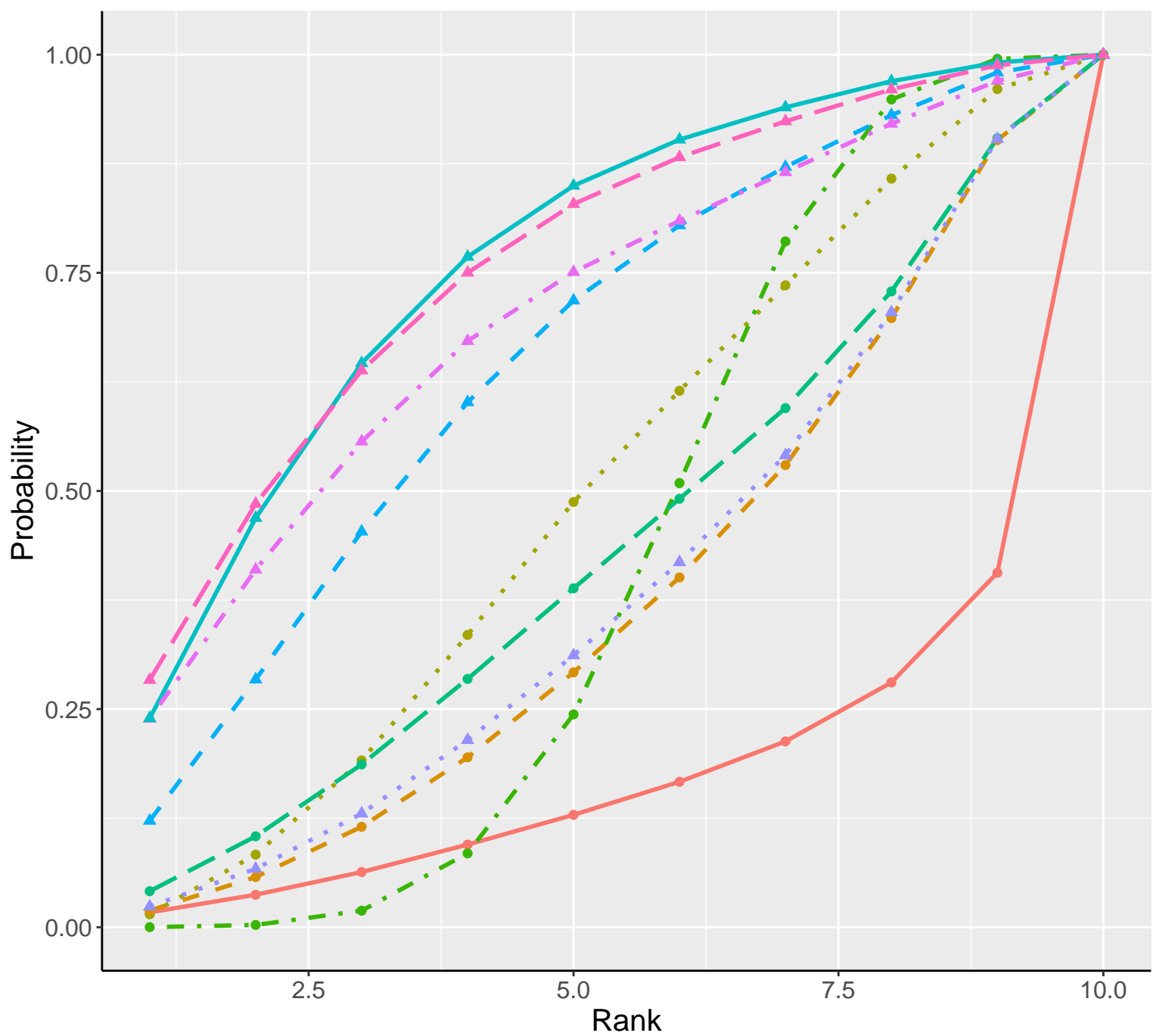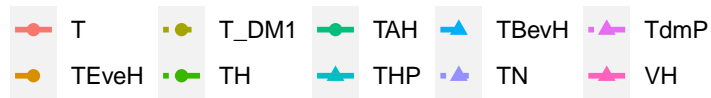

Supplement: Supplementary file 1 [file DataSheet_1.zip › Supplementary data 5D-2 SUCRA results for PFS in first line HR+ studies.pdf]

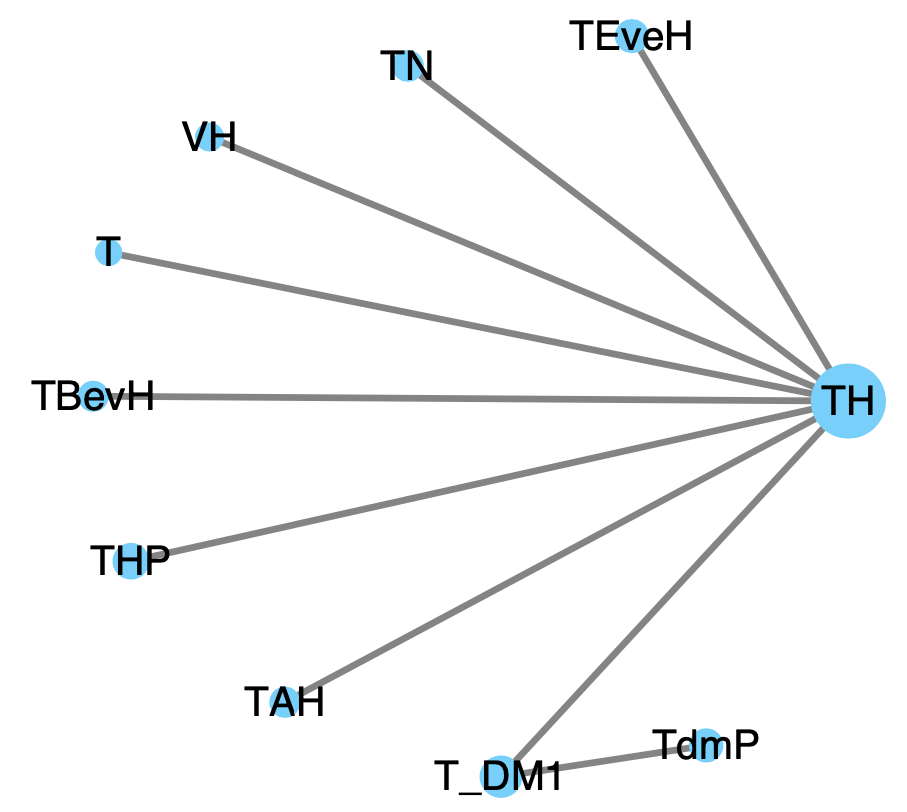

Supplement: Supplementary file 1 [file DataSheet_1.zip › Supplementary data 6A Net-work plot of PFS in first line HR- studies.png]

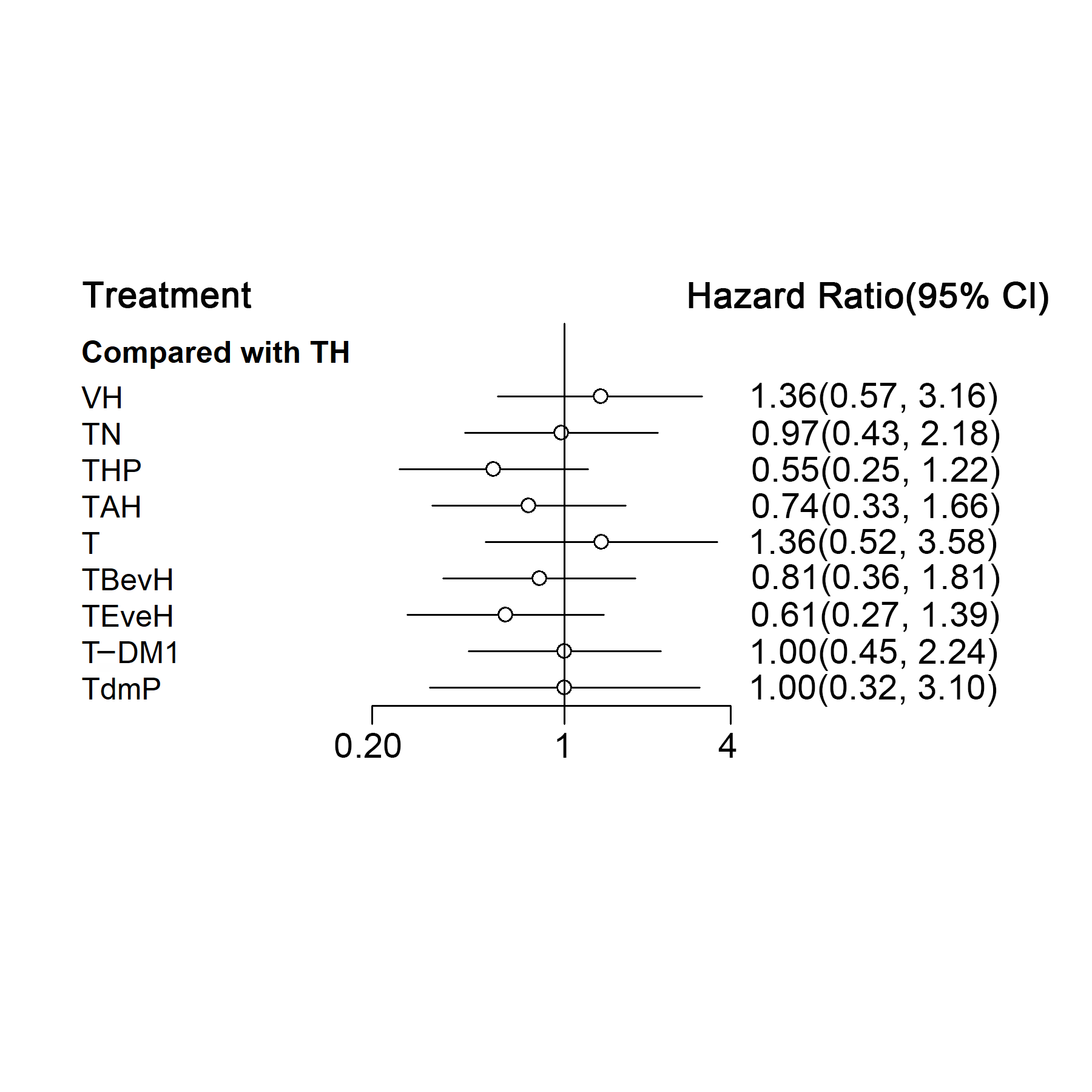

Supplement: Supplementary file 1 [file DataSheet_1.zip › Supplementary data 6B Forest plot of PFS in first line HR- studies.tiff]

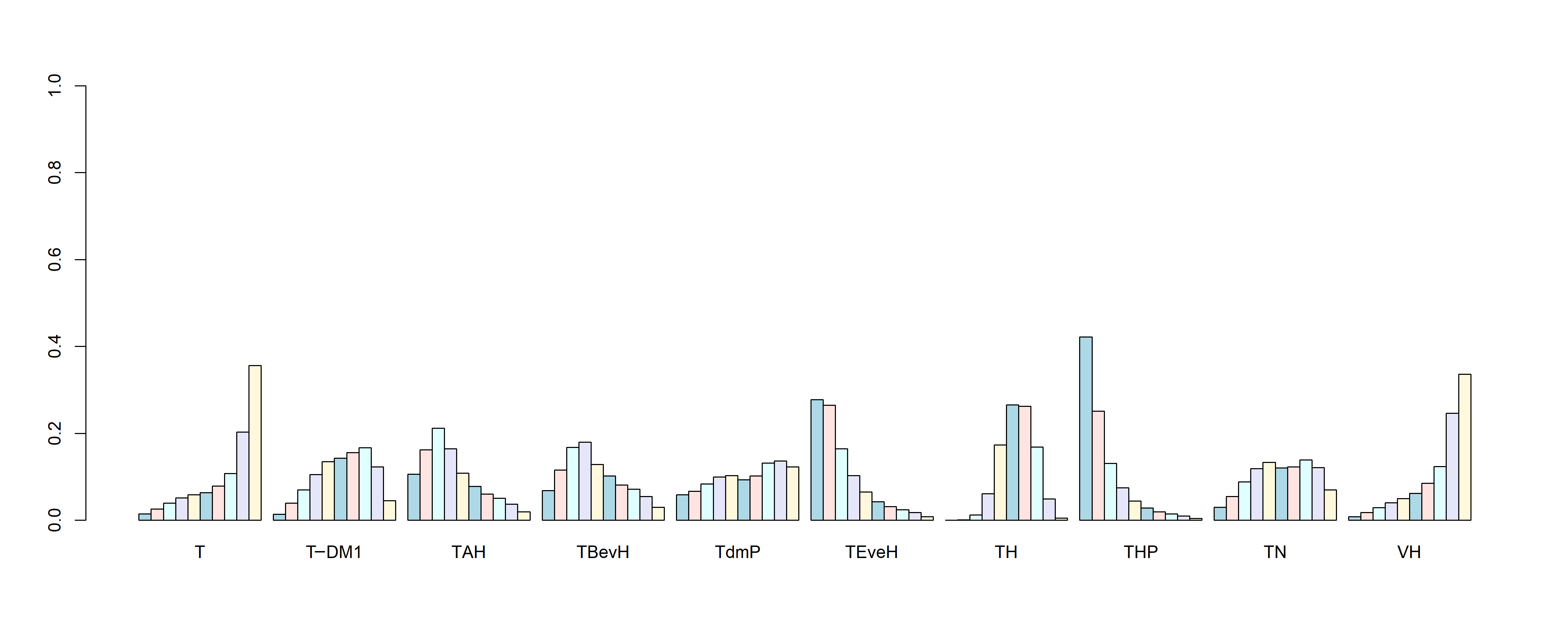

Supplement: Supplementary file 1 [file DataSheet_1.zip › Supplementary data 6D-1 Ranking histogram for PFS in first line HR- studies.tiff]

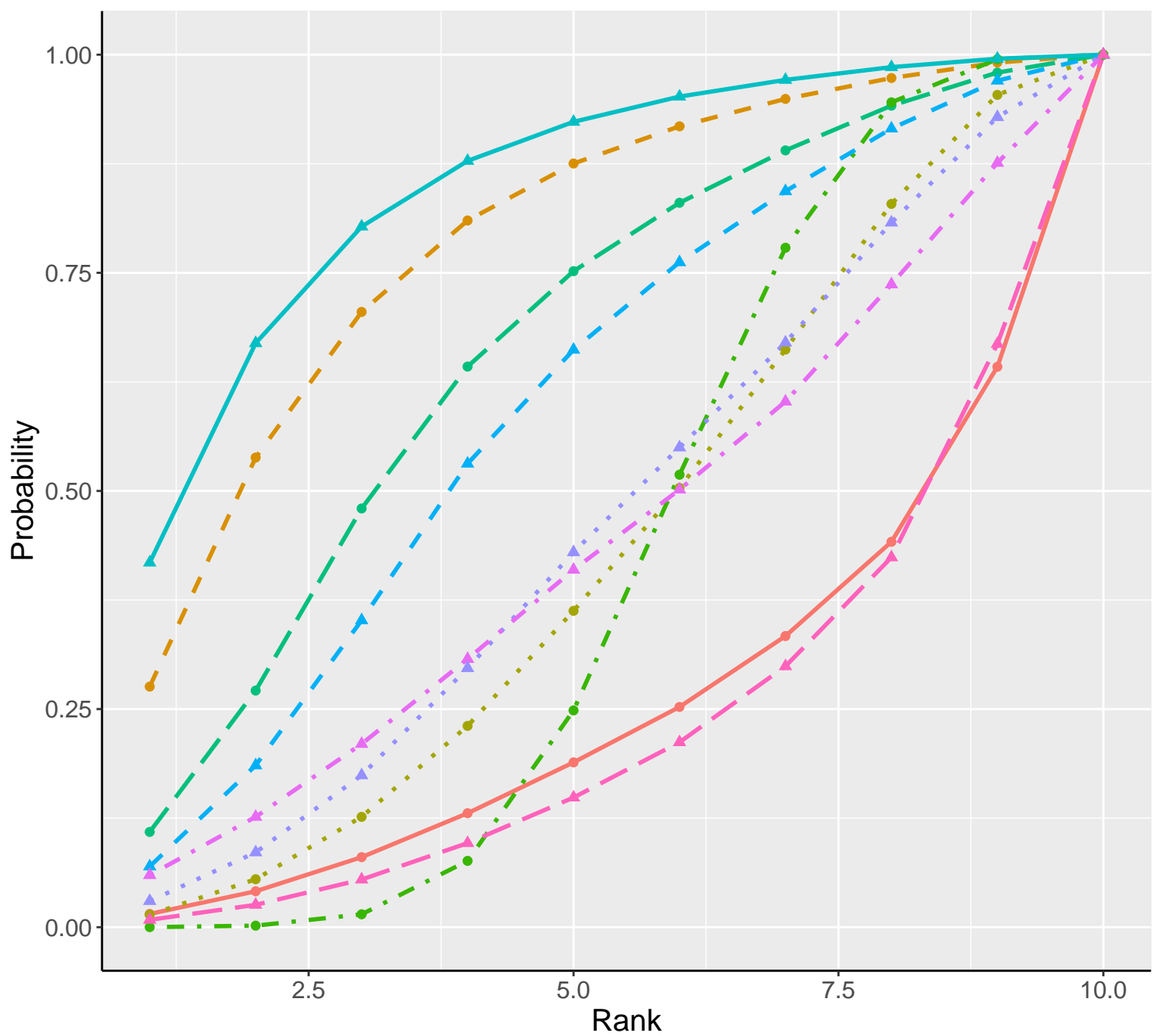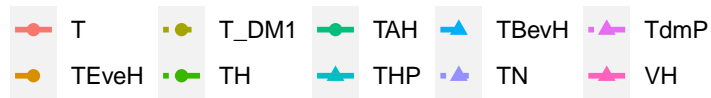

Supplement: Supplementary file 1 [file DataSheet_1.zip › Supplementary data 6D-2 SUCRA results for PFS in first line HR- studies.pdf]

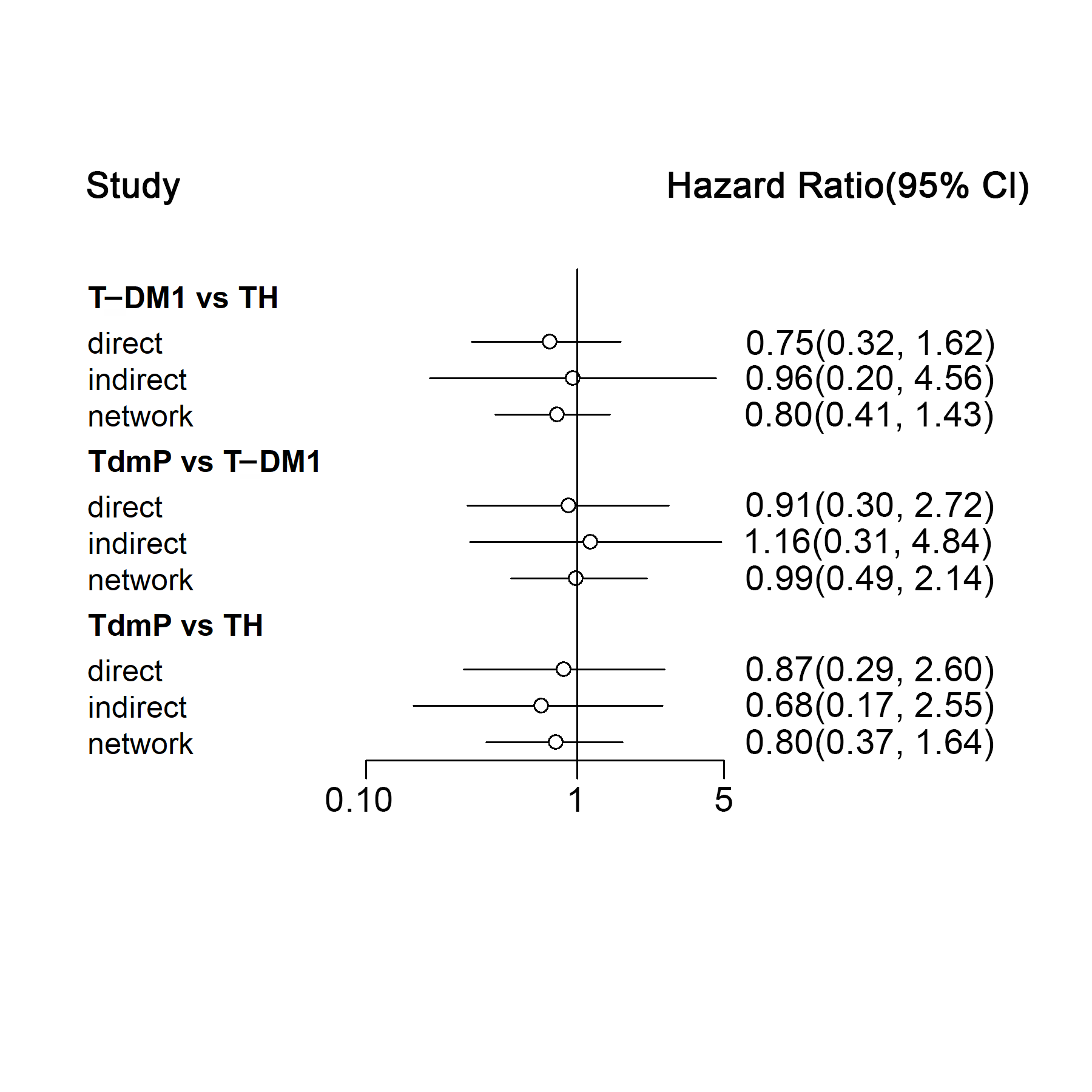

Supplement: Supplementary file 1 [file DataSheet_1.zip › Supplementary data 4B Inconsistency test of PFS in first line studies.tiff]

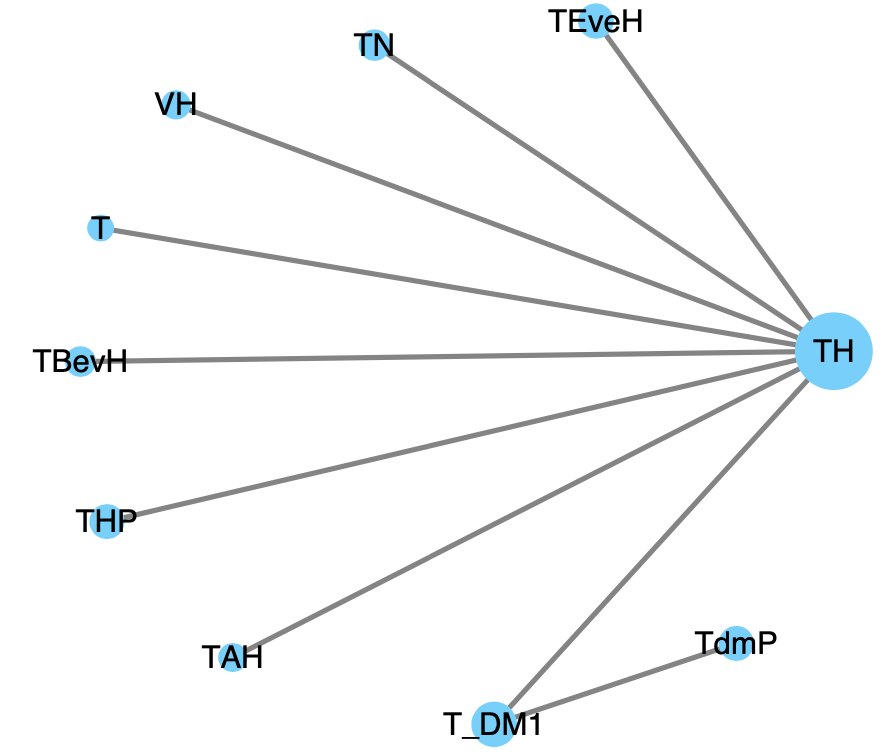

Supplement: Supplementary file 1 [file DataSheet_1.zip › Supplementary data 5A Net-work plot of PFS in first line HR+ studies.png]

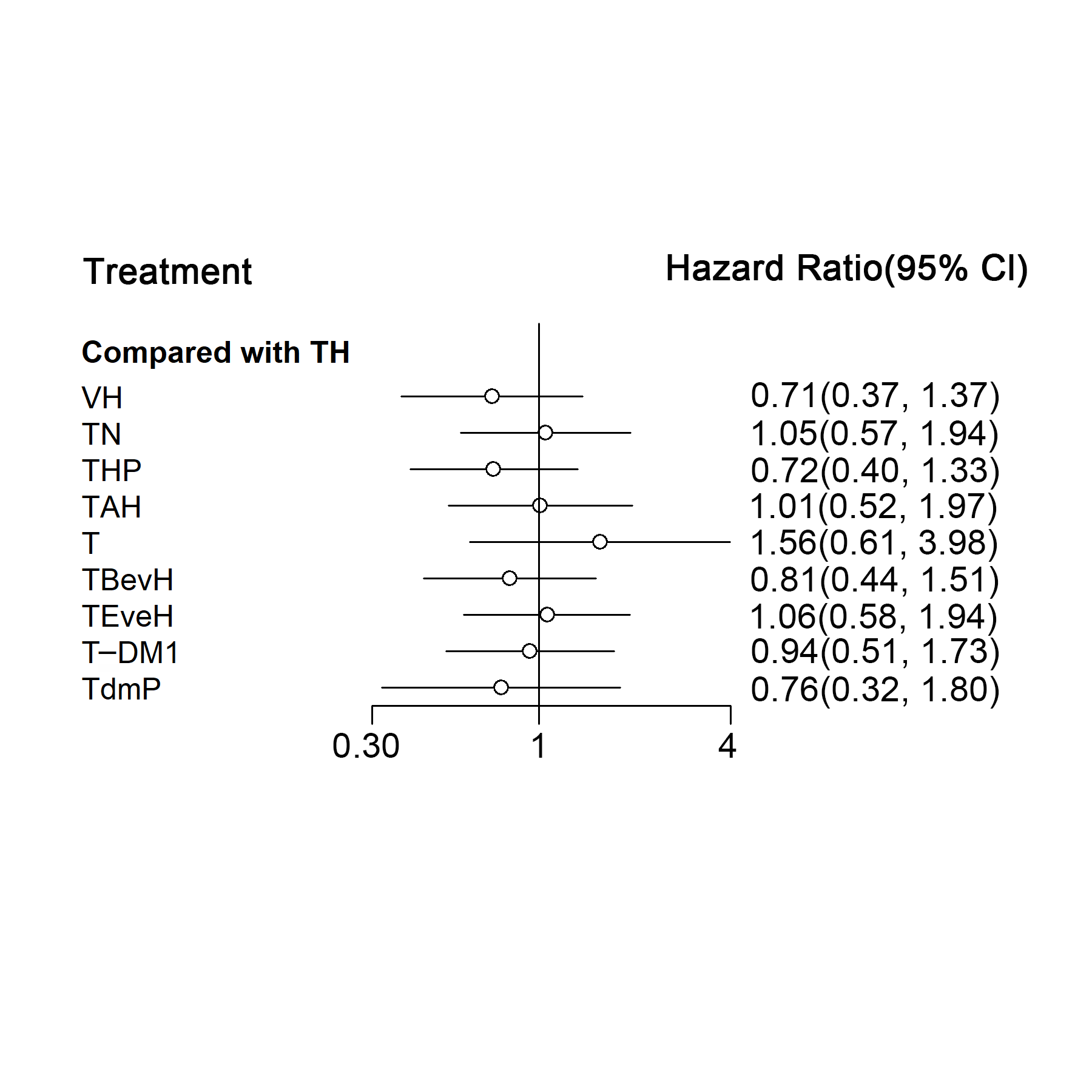

Supplement: Supplementary file 1 [file DataSheet_1.zip › Supplementary data 5B Forest plot of PFS in first line HR+ studies.tiff]

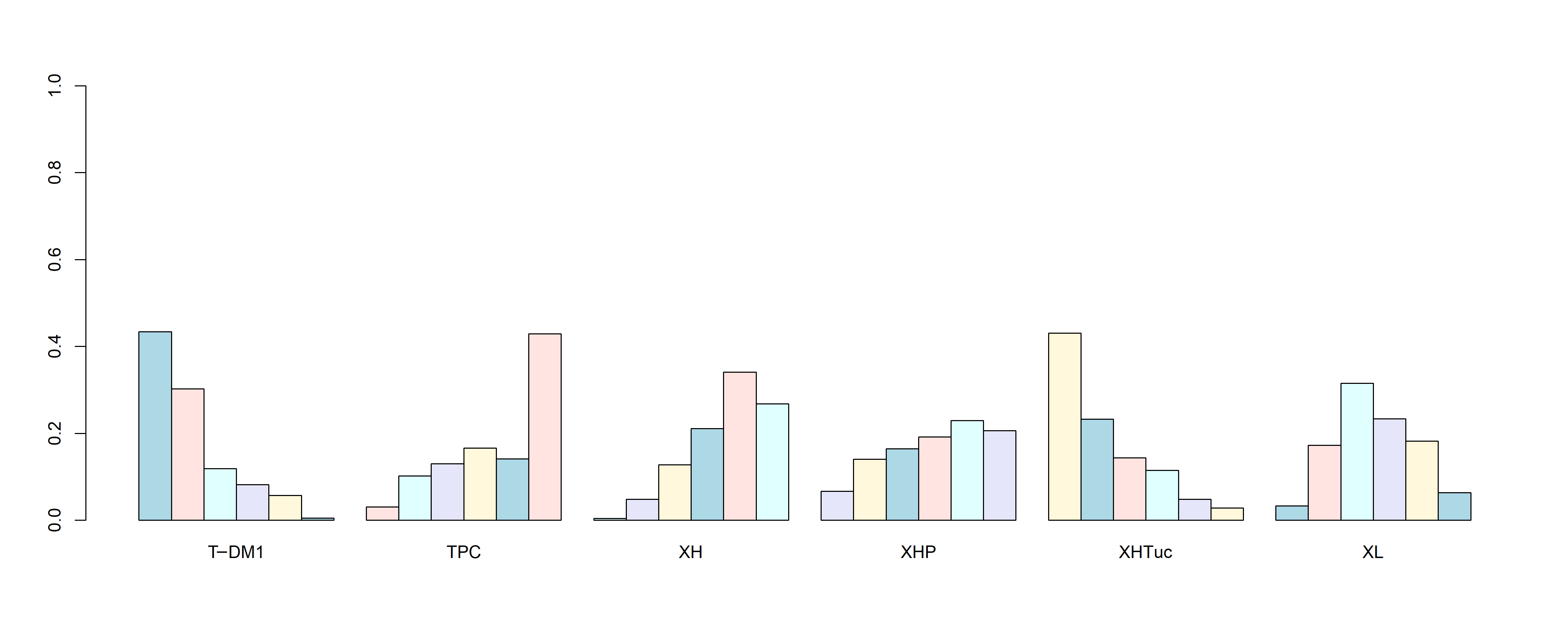

Supplement: Supplementary file 2 [file DataSheet_2.zip › Supplementary data 9D-1 Ranking histogram for PFS in second or other line HR+ studies.tiff]

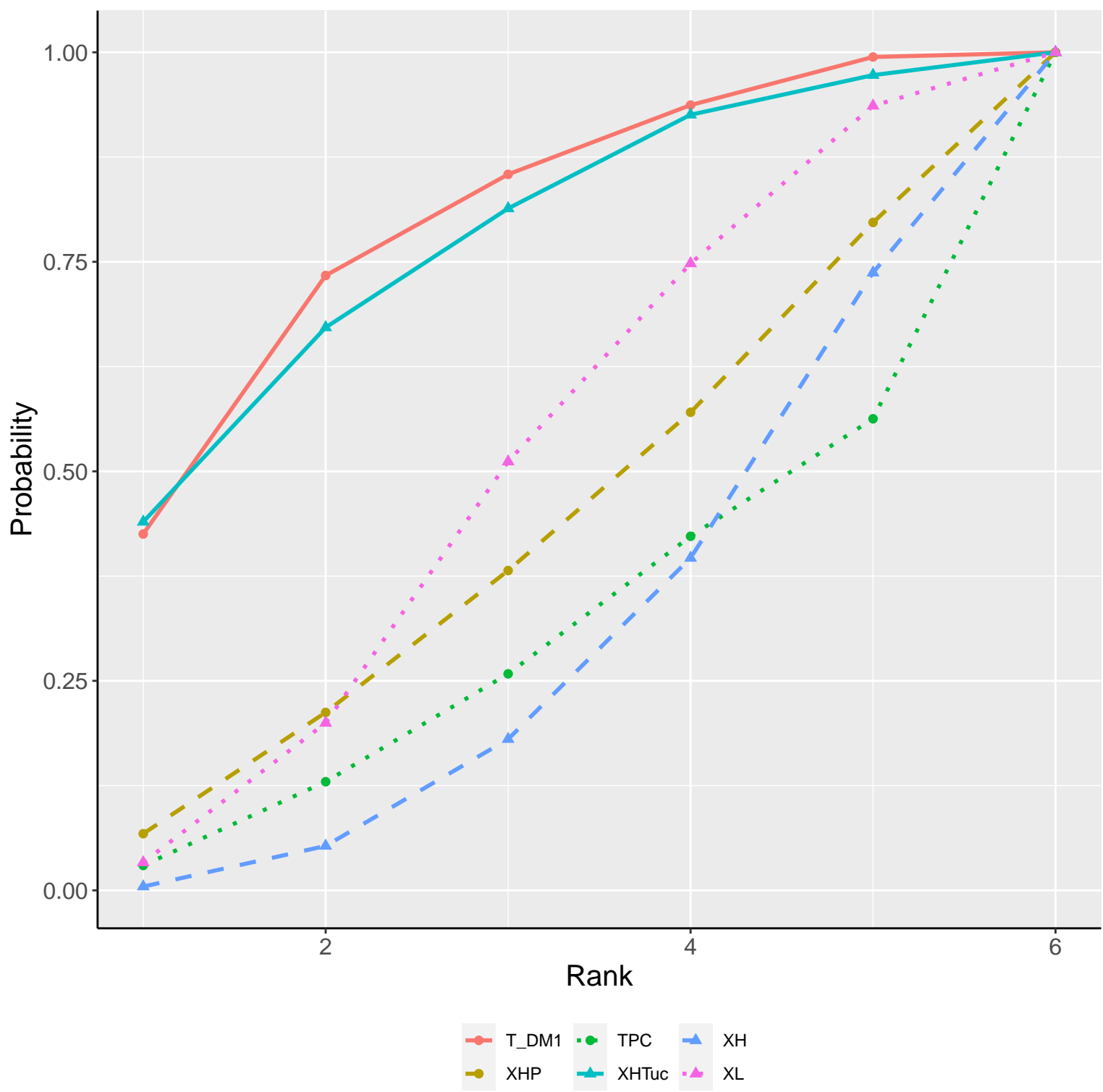

Supplement: Supplementary file 2 [file DataSheet_2.zip › Supplementary data 9D-2 SUCRA results for PFS in second or other line HR+ studies.pdf]

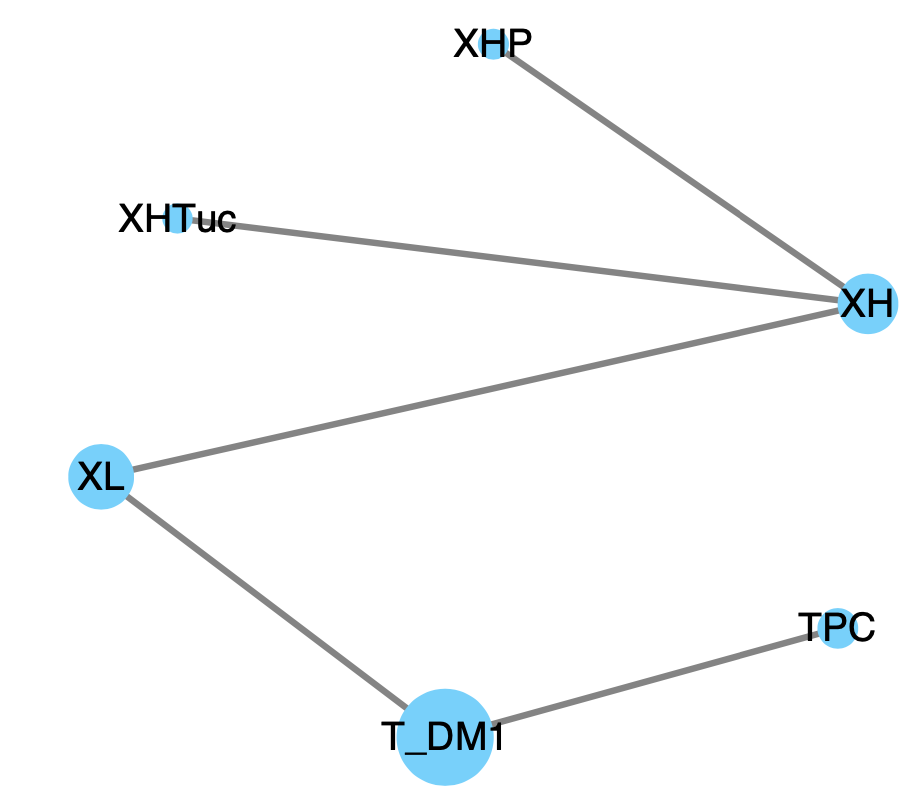

Supplement: Supplementary file 2 [file DataSheet_2.zip › Supplementary data 10A Net-work plot of PFS in second or other line HR- studies.png]

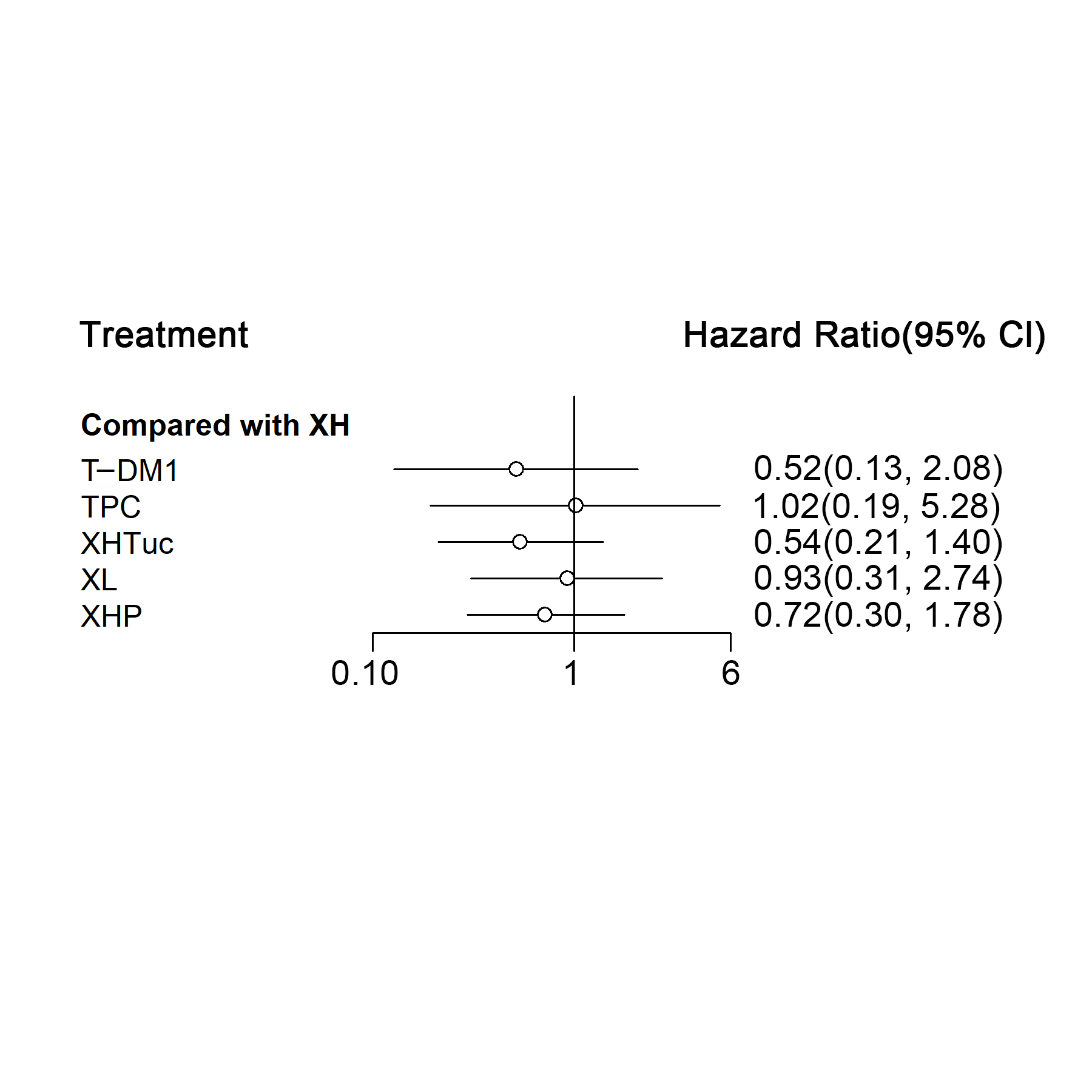

Supplement: Supplementary file 2 [file DataSheet_2.zip › Supplementary data 10B Forest plot of PFS in second or other line HR- studies.tiff]

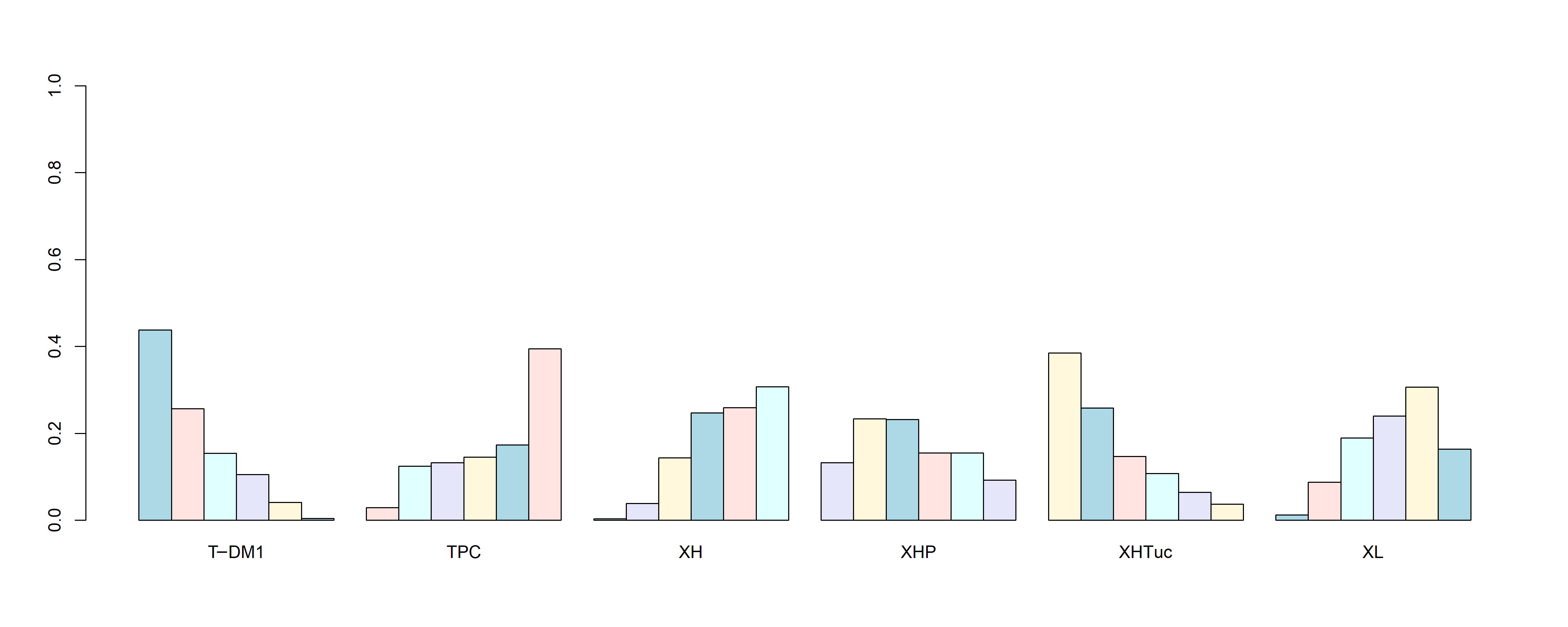

Supplement: Supplementary file 2 [file DataSheet_2.zip › Supplementary data 10D-1 Ranking histogram for PFS in second or other line HR- studies.tiff]

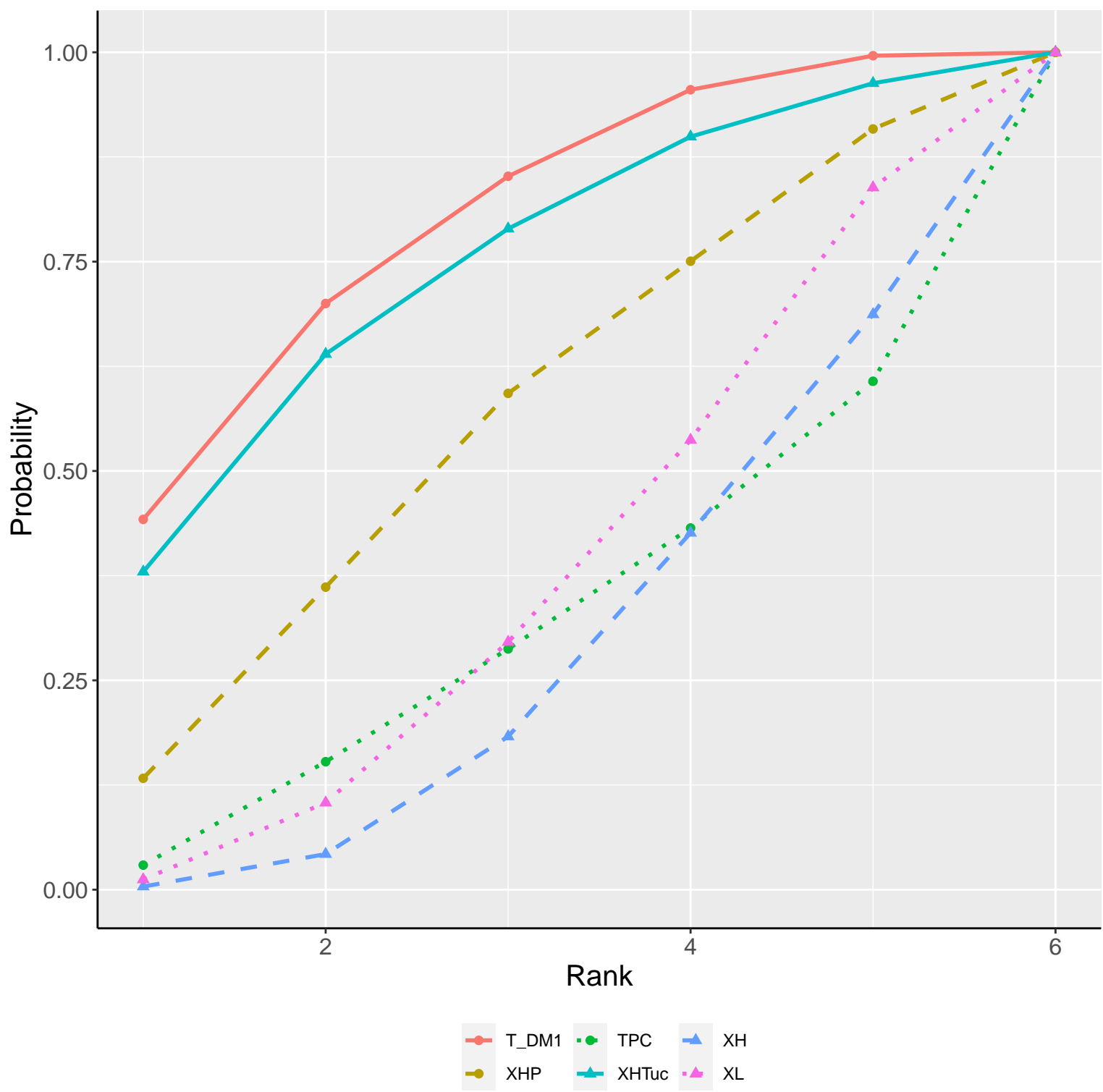

Supplement: Supplementary file 2 [file DataSheet_2.zip › Supplementary data 10D-2 SUCRA results for PFS in second or other line HR- studies.pdf]

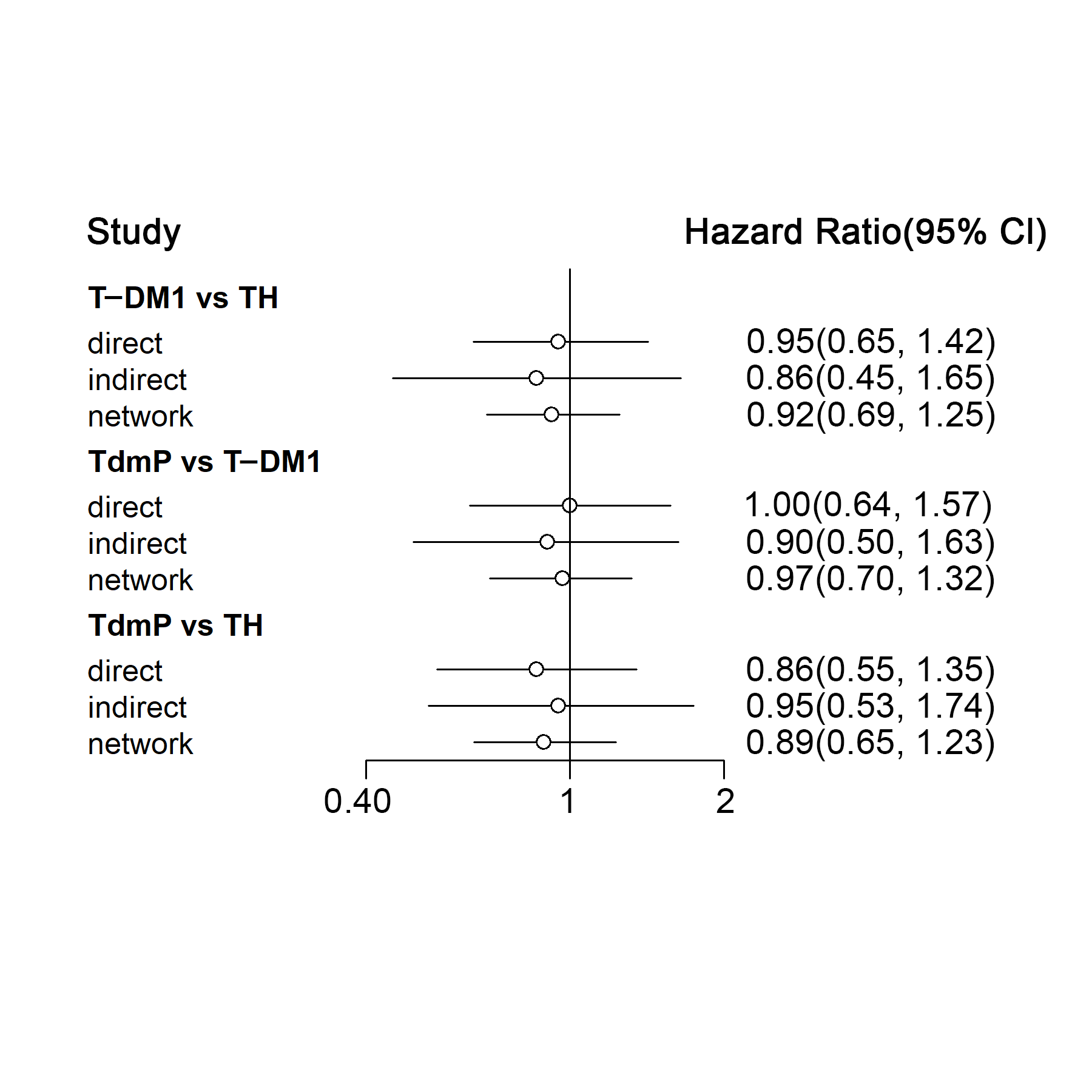

Supplement: Supplementary file 2 [file DataSheet_2.zip › Supplementary data 12B Inconsistency test of OS in first line studies.tiff]

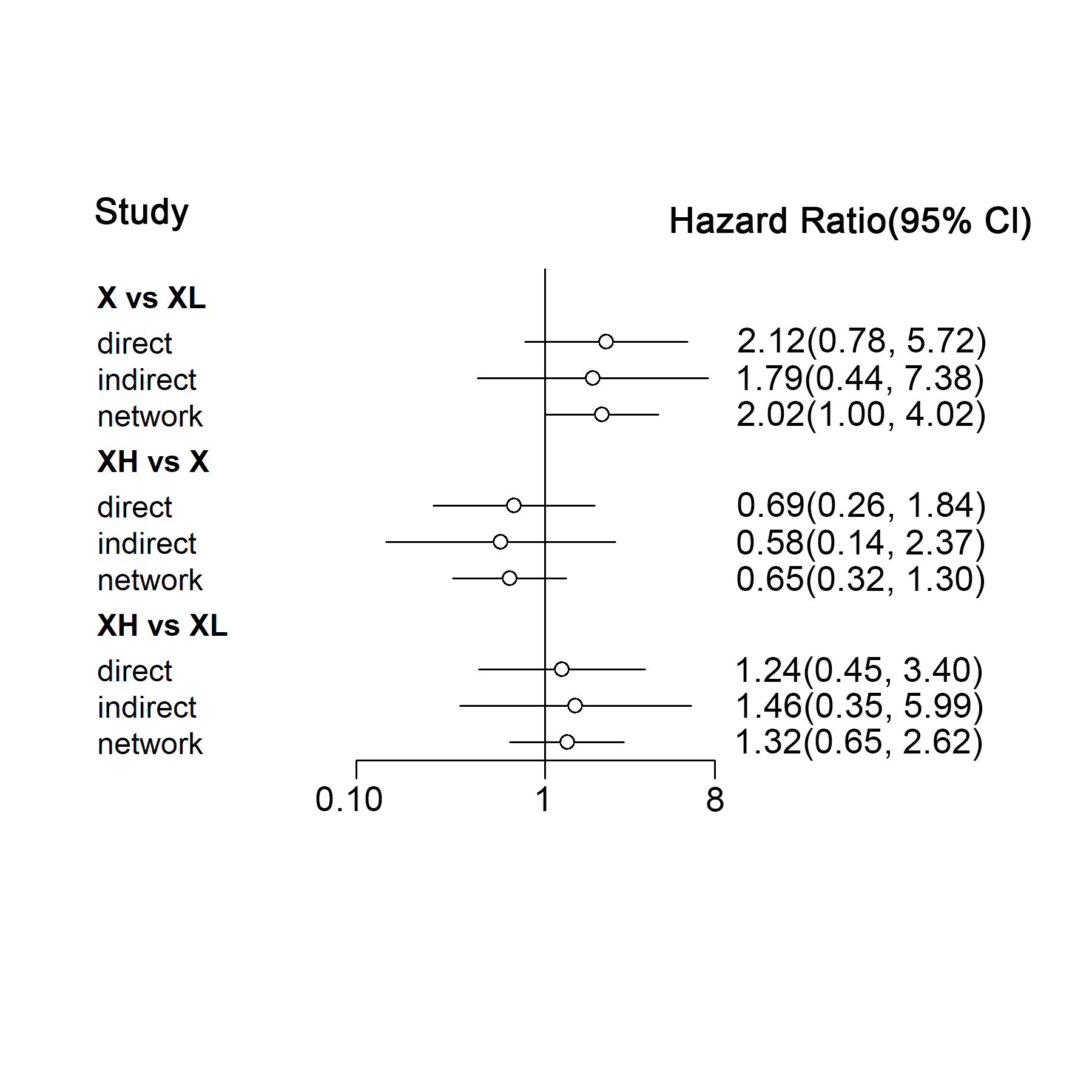

Supplement: Supplementary file 2 [file DataSheet_2.zip › Supplementary data 8B Inconsistency test of PFS in second or other line studies.tiff]

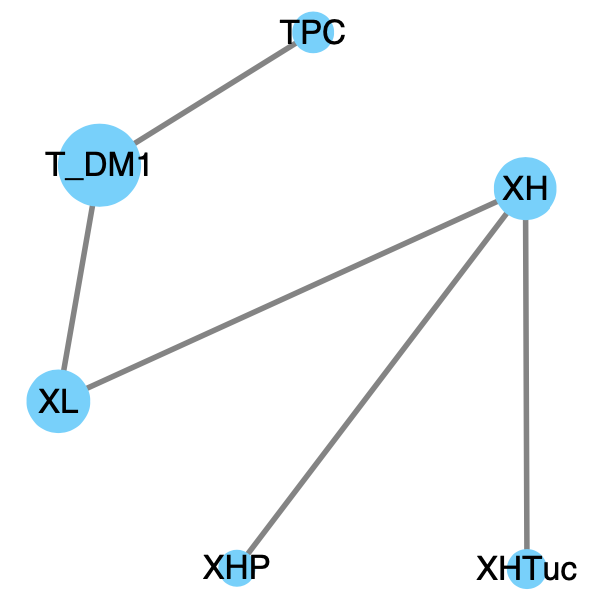

Supplement: Supplementary file 2 [file DataSheet_2.zip › Supplementary data 9A Net-work plot of PFS in second or other line HR+ studies.png]

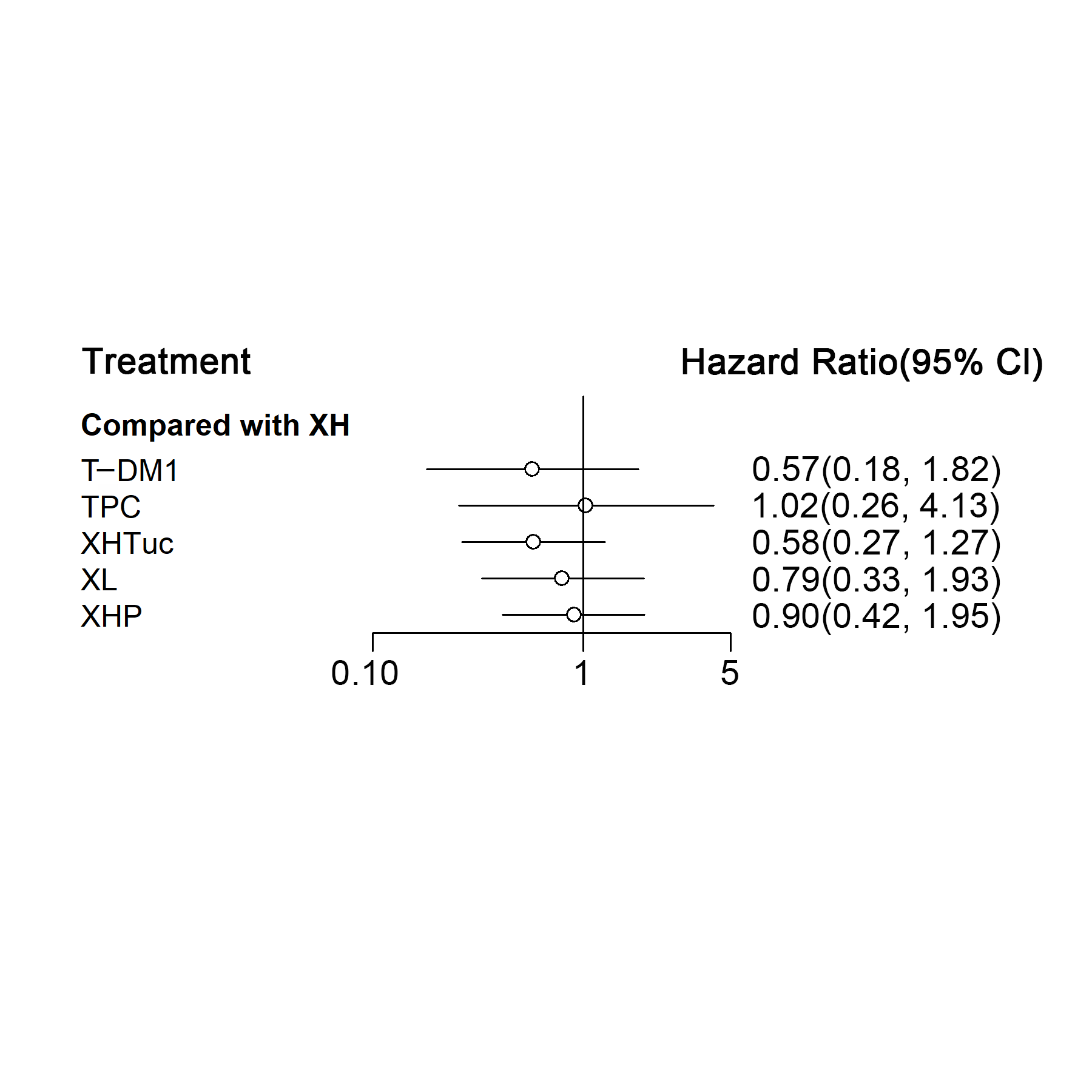

Supplement: Supplementary file 2 [file DataSheet_2.zip › Supplementary data 9B Forest plot of PFS in second or other line HR+ studies.tiff]

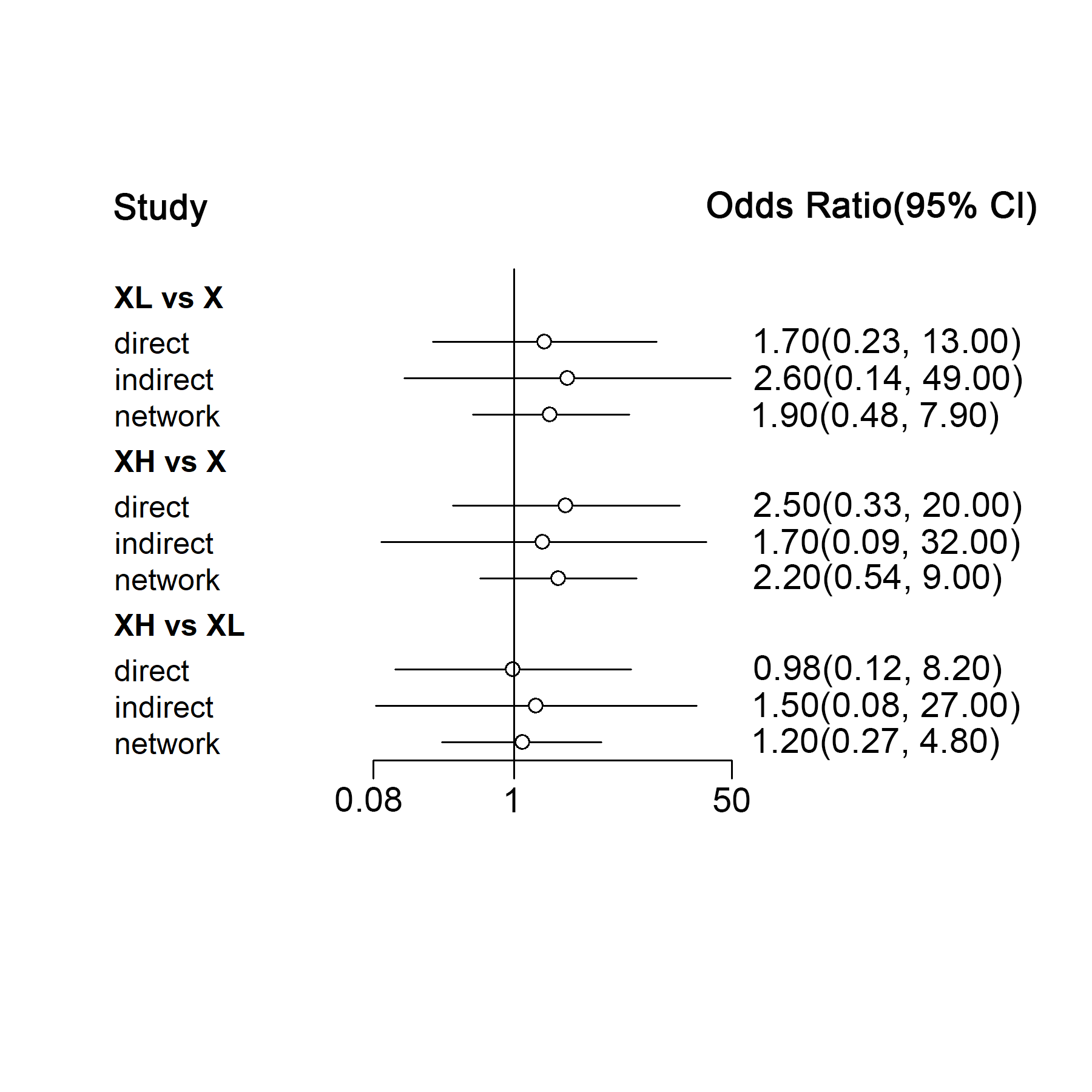

Supplement: Supplementary file 3 [file DataSheet_3.zip › Supplementary data 18B Inconsistency test of ORR in second or other line studies.tiff]

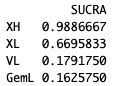

Supplement: Supplementary file 3 [file DataSheet_3.zip › Supplementary data 19A SUCRA results for leucopenia in second or other line studies.png]

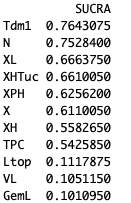

Supplement: Supplementary file 3 [file DataSheet_3.zip › Supplementary data 19B SUCRA results for neutropenia in second or other line studies.png]

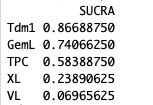

Supplement: Supplementary file 3 [file DataSheet_3.zip › Supplementary data 19C SUCRA results for febrile neutropenia in second or other line studies.png]

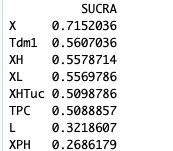

Supplement: Supplementary file 3 [file DataSheet_3.zip › Supplementary data 19D SUCRA results for cardiac adverse events in second or other line studies.png]

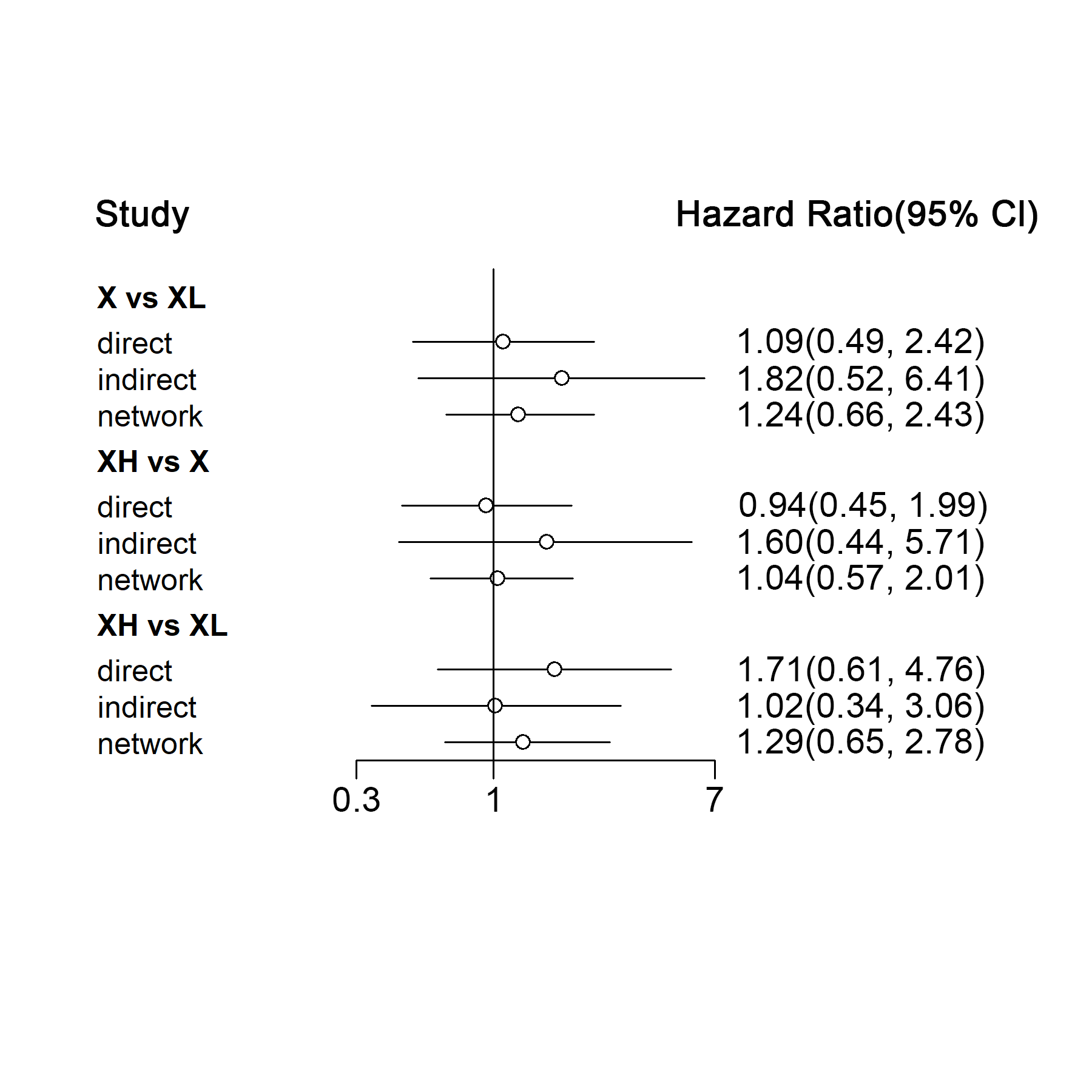

Supplement: Supplementary file 3 [file DataSheet_3.zip › Supplementary data 14B Inconsistency test of OS in second or other line studies.tiff]
