# Supplementary material for: Efficacy and Safety of Anti-HER2 Agents in Combination With Chemotherapy for Metastatic HER2-Positive Breast Cancer Patient: A Network Meta-Analysis
Source: Front Oncol. 2021 Aug 19;11:731210. doi: 10.3389/fonc.2021.731210 (PMC8416996; doi:10.3389/fonc.2021.731210)
Supplement: Supplementary file 2 [file DataSheet_2.zip › Supplementary data 9E Trace plot and density plot of PFS in second or other line HR+ studies.pdf]

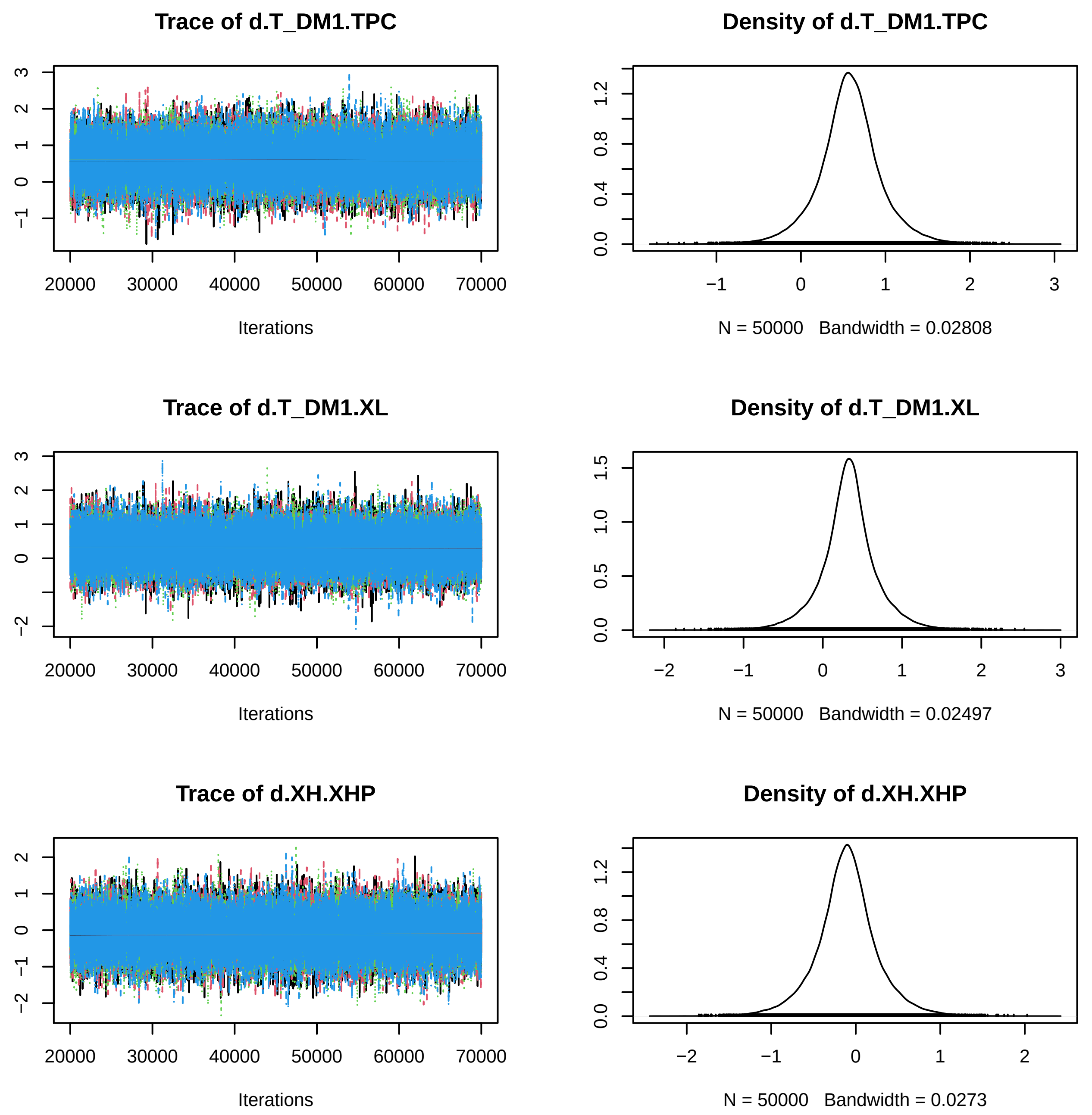
Trace of d.T_DM1.TPC
Iterations
Density of d.T_DM1.TPC
N = 50000 Bandwidth = 0.02808
Trace of d.T_DM1.XL
Iterations
Density of d.T_DM1.XL
N = 50000 Bandwidth = 0.02497
Trace of d.XH.XHP
Iterations
Density of d.XH.XHP
N = 50000 Bandwidth = 0.0273

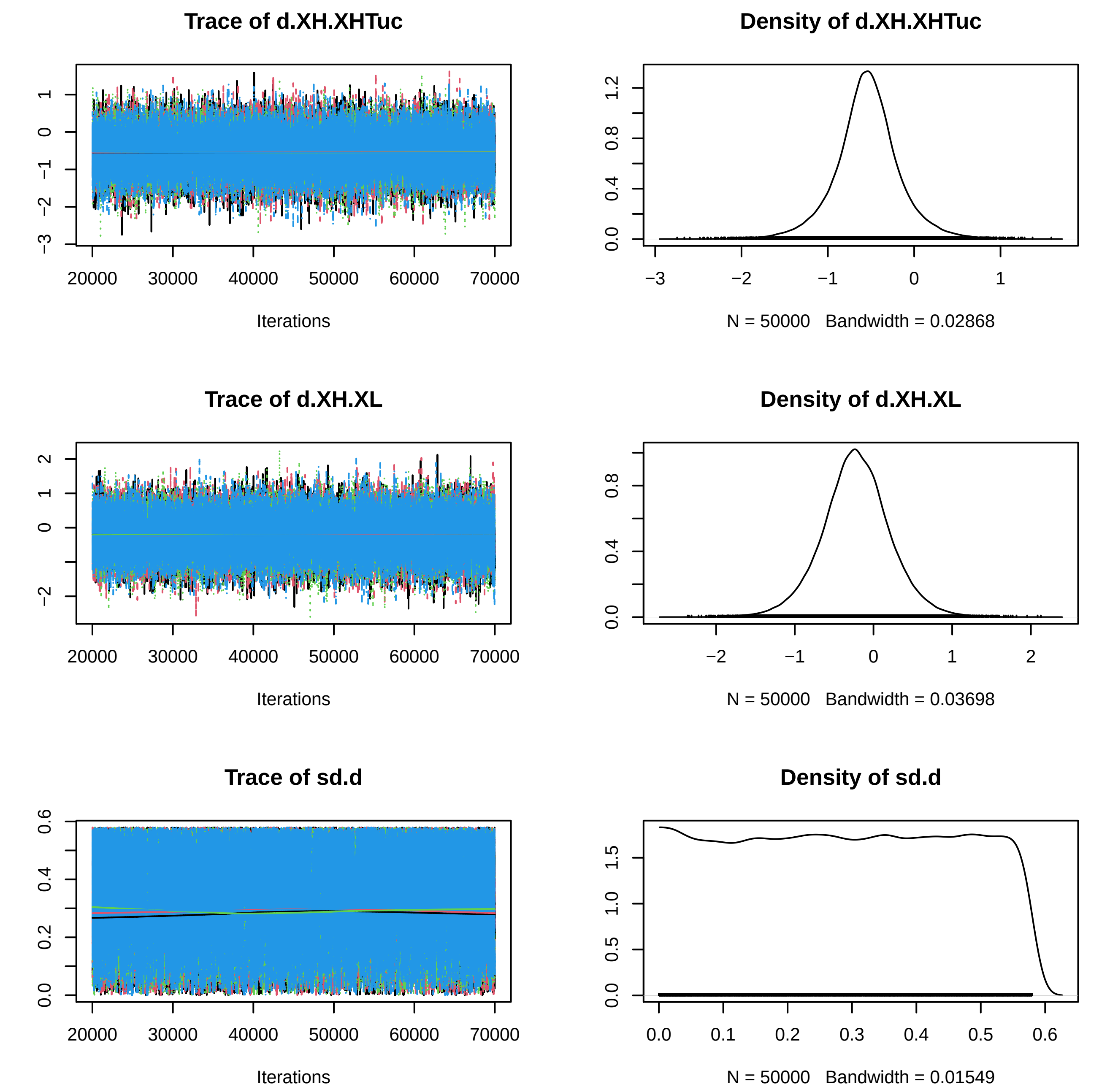
Trace of d.XH.XHTuc
Density of d.XH.XHTuc
Iterations
N = 50000   Bandwidth = 0.02868
Trace of d.XH.XL
Density of d.XH.XL
Iterations
N = 50000   Bandwidth = 0.03698
Trace of sd.d
Density of sd.d
Iterations
N = 50000   Bandwidth = 0.01549
