# Supplementary material for: Efficacy and Safety of Anti-HER2 Agents in Combination With Chemotherapy for Metastatic HER2-Positive Breast Cancer Patient: A Network Meta-Analysis
Source: Front Oncol. 2021 Aug 19;11:731210. doi: 10.3389/fonc.2021.731210 (PMC8416996; doi:10.3389/fonc.2021.731210)
Supplement: Supplementary file 2 [file DataSheet_2.zip › Supplementary data 10E Trace plot and density plot of PFS in second or other line HR- studies.pdf]

Trace of d.T_DM1.TPC

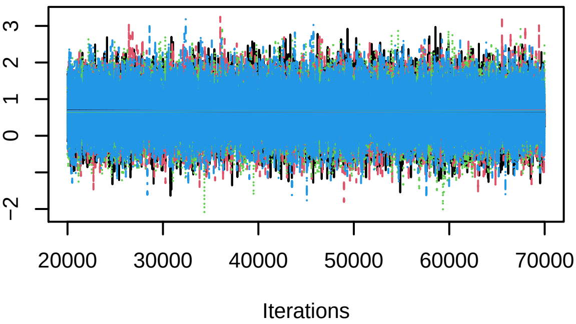

Density of d.T_DM1.TPC

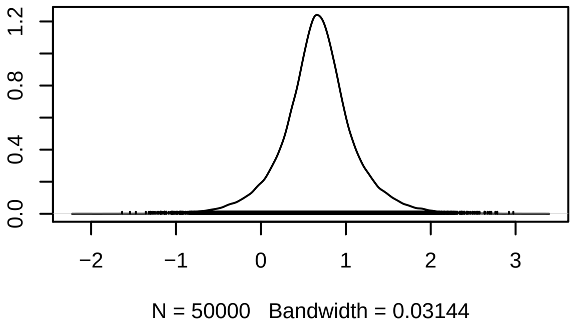

Trace of d.T_DM1.XL

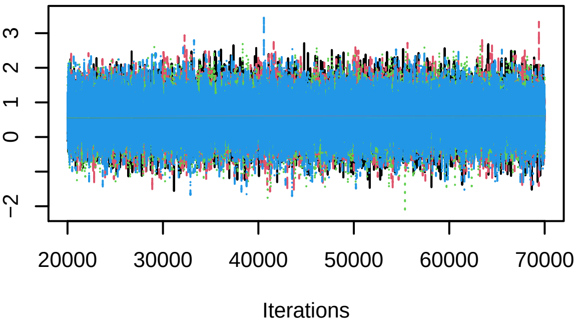

Density of d.T_DM1.XL

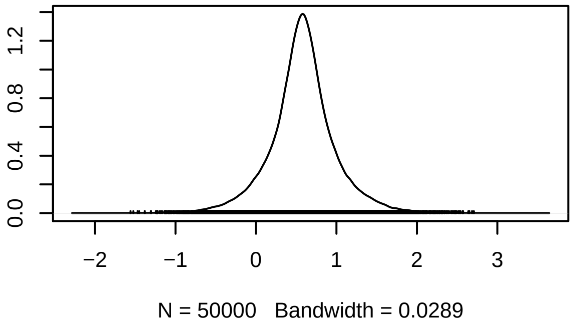

Trace of d.XH.XHP

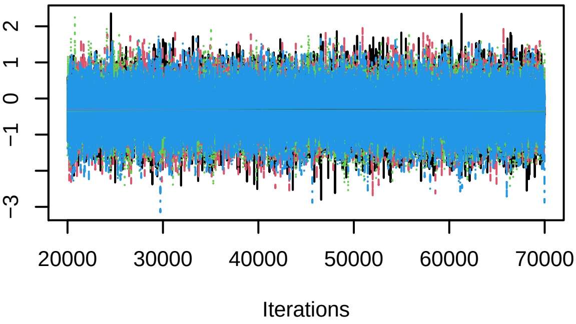

Density of d.XH.XHP

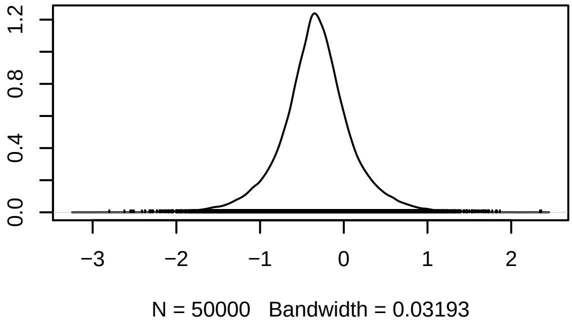

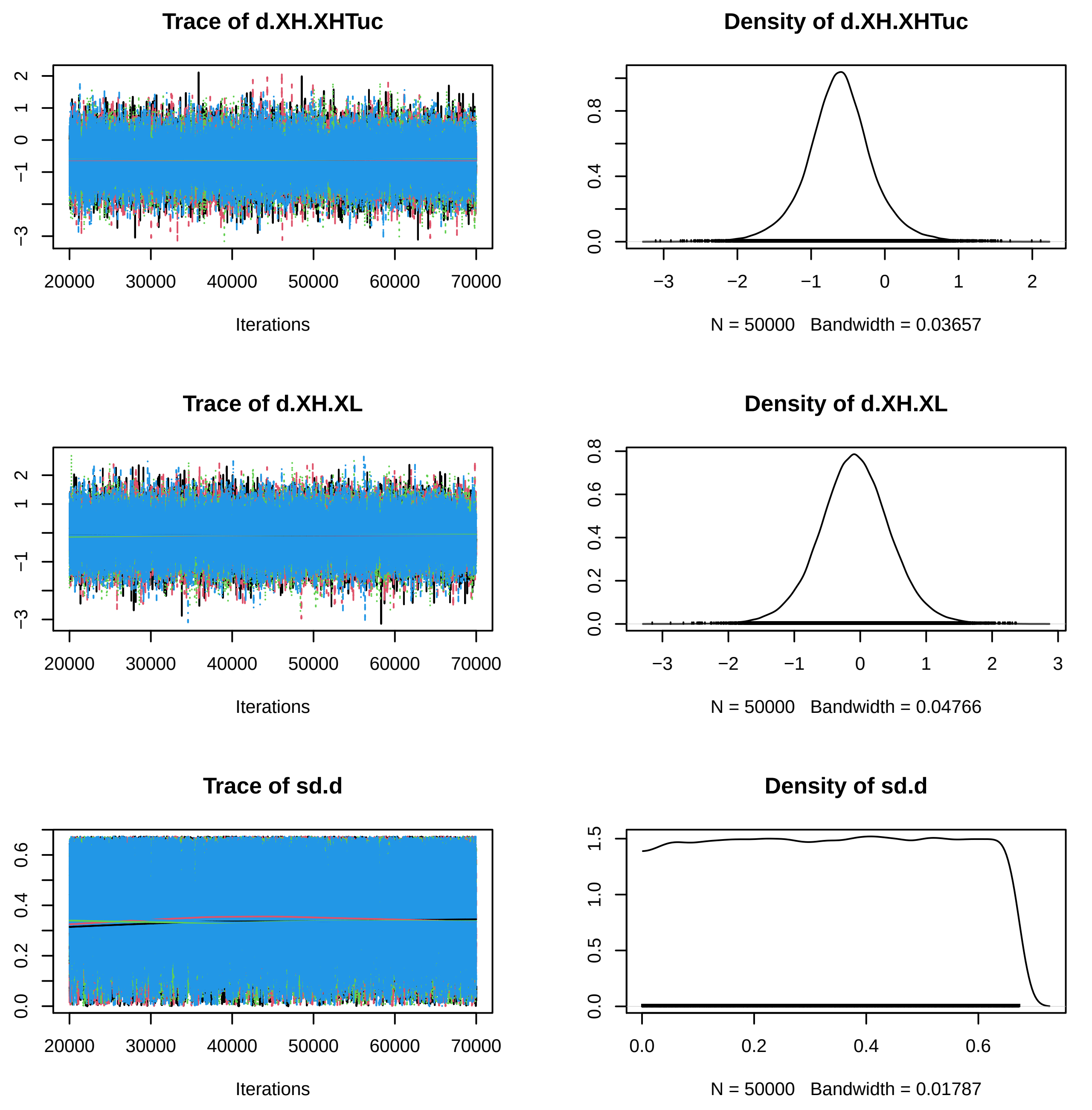

Trace of d.XH.XHTuc
Density of d.XH.XHTuc
Iterations
N = 50000 Bandwidth = 0.03657
Trace of d.XH.XL
Density of d.XH.XL
Iterations
N = 50000 Bandwidth = 0.04766
Trace of sd.d
Density of sd.d
Iterations
N = 50000 Bandwidth = 0.01787
