# Supplementary material for: Efficacy and Safety of Anti-HER2 Agents in Combination With Chemotherapy for Metastatic HER2-Positive Breast Cancer Patient: A Network Meta-Analysis
Source: Front Oncol. 2021 Aug 19;11:731210. doi: 10.3389/fonc.2021.731210 (PMC8416996; doi:10.3389/fonc.2021.731210)
Supplement: Supplementary file 2 [file DataSheet_2.zip › Supplementary data 12A Trace plot and density plot of OS in first line studies.pdf]

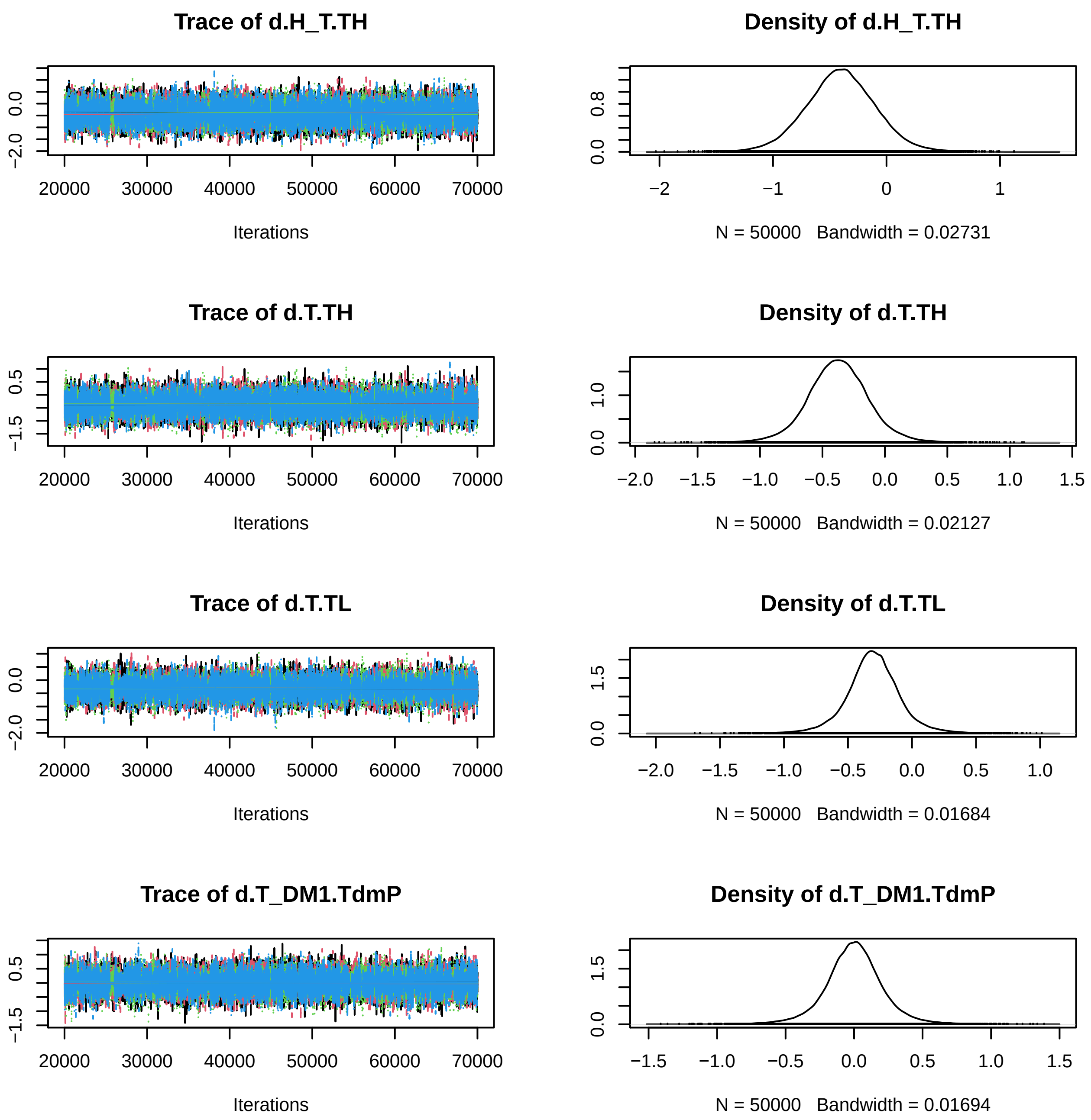

Trace of d.H_T.TH
Density of d.H_T.TH
N = 50000 Bandwidth = 0.02731
Trace of d.T.TH
Density of d.T.TH
N = 50000 Bandwidth = 0.02127
Trace of d.T.TL
Density of d.T.TL
N = 50000 Bandwidth = 0.01684
Trace of d.T_DM1.TdmP
Density of d.T_DM1.TdmP
N = 50000 Bandwidth = 0.01694
Iterations

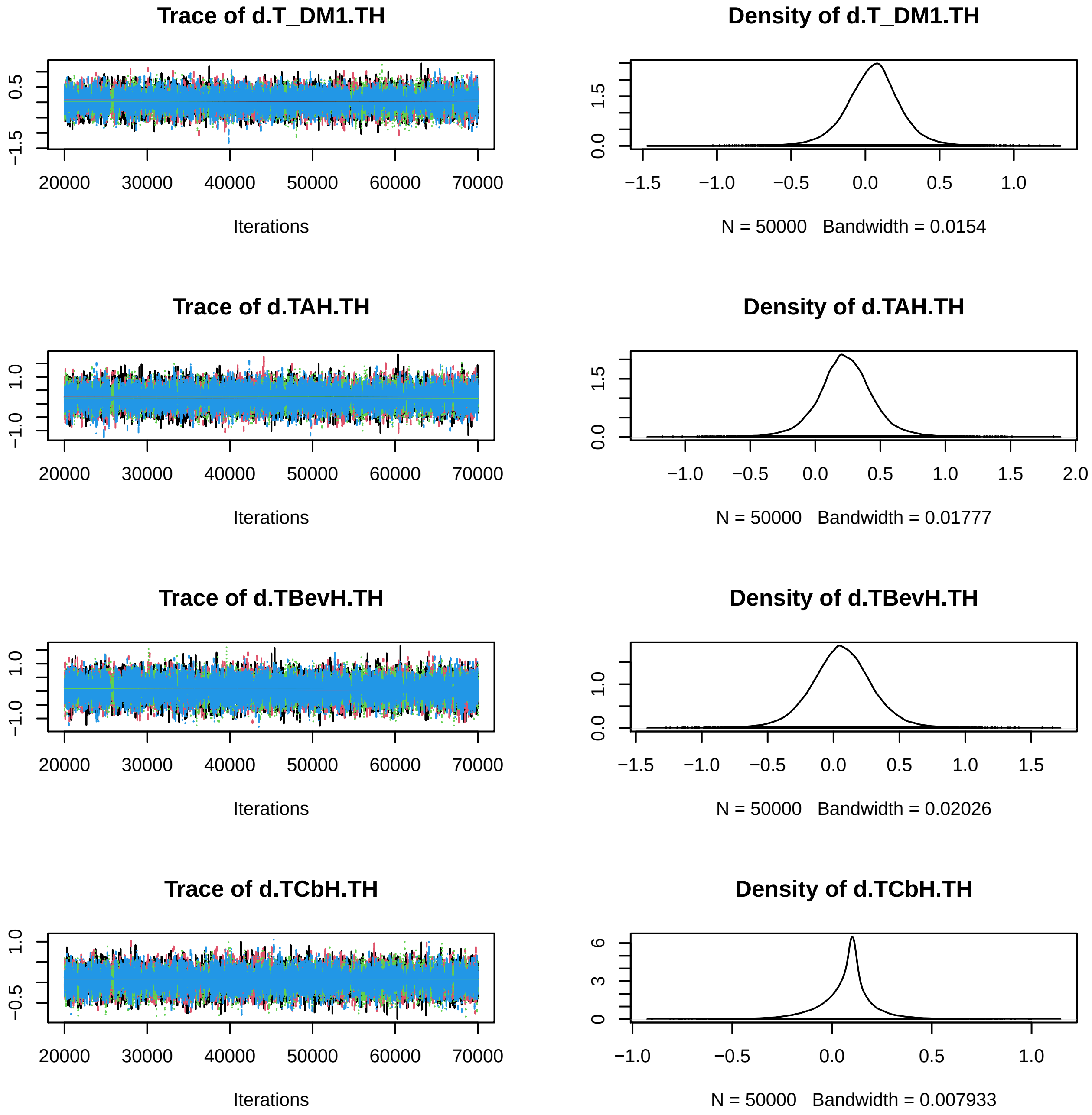
Trace of d.T_DM1.TH
0.5
−1.5
20000 30000 40000 50000 60000 70000
Iterations
Density of d.T_DM1.TH
1.5
0.0
−1.5 −1.0 −0.5 0.0 0.5 1.0
N = 50000 Bandwidth = 0.0154
Trace of d.TAH.TH
1.0
−1.0
20000 30000 40000 50000 60000 70000
Iterations
Density of d.TAH.TH
1.5
0.0
−1.0 −0.5 0.0 0.5 1.0 1.5 2.0
N = 50000 Bandwidth = 0.01777
Trace of d.TBevH.TH
1.0
−1.0
20000 30000 40000 50000 60000 70000
Iterations
Density of d.TBevH.TH
1.0
0.0
−1.5 −1.0 −0.5 0.0 0.5 1.0 1.5
N = 50000 Bandwidth = 0.02026
Trace of d.TCbH.TH
1.0
−0.5
20000 30000 40000 50000 60000 70000
Iterations
Density of d.TCbH.TH
6
3
0
−1.0 −0.5 0.0 0.5 1.0
N = 50000 Bandwidth = 0.007933

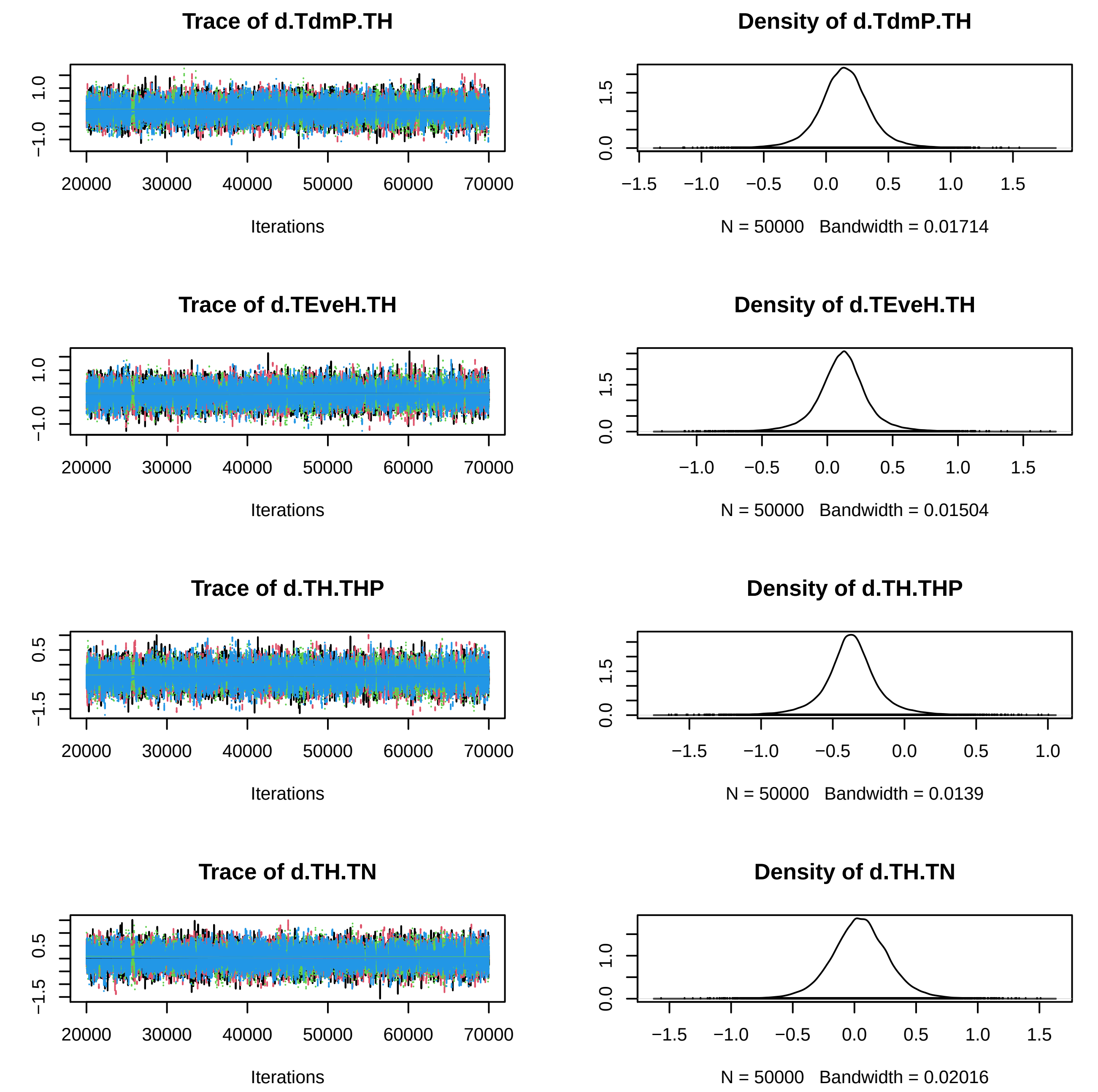

Trace of d.TdmP.TH
Density of d.TdmP.TH
Iterations
N = 50000 Bandwidth = 0.01714
Trace of d.TEveH.TH
Density of d.TEveH.TH
Iterations
N = 50000 Bandwidth = 0.01504
Trace of d.TH.THP
Density of d.TH.THP
Iterations
N = 50000 Bandwidth = 0.0139
Trace of d.TH.TN
Density of d.TH.TN
Iterations
N = 50000 Bandwidth = 0.02016

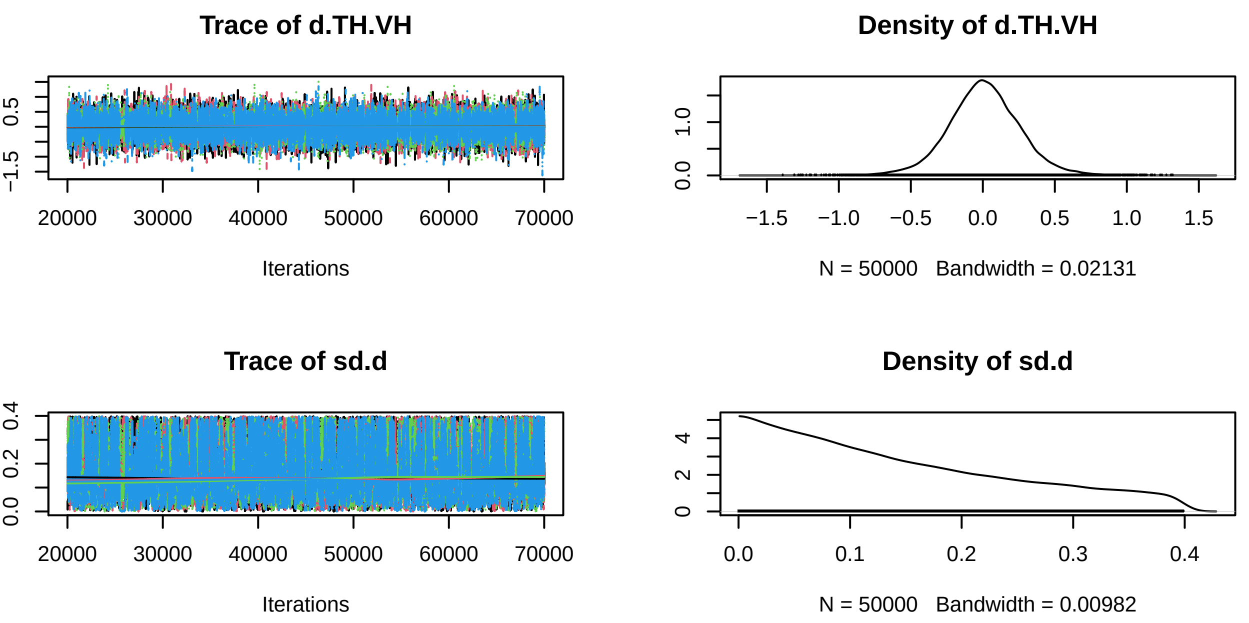
Trace of d.TH.VH
Iterations
Density of d.TH.VH
N = 50000 Bandwidth = 0.02131
Trace of sd.d
Iterations
Density of sd.d
N = 50000 Bandwidth = 0.00982
20000 30000 40000 50000 60000 70000
−1.5 −1.0 −0.5 0.0 0.5 1.0 1.5
0.0 0.1 0.2 0.3 0.4
