# Supplementary material for: Efficacy and Safety of Anti-HER2 Agents in Combination With Chemotherapy for Metastatic HER2-Positive Breast Cancer Patient: A Network Meta-Analysis
Source: Front Oncol. 2021 Aug 19;11:731210. doi: 10.3389/fonc.2021.731210 (PMC8416996; doi:10.3389/fonc.2021.731210)
Supplement: Supplementary file 2 [file DataSheet_2.zip › Supplementary data 8A Trace plot and density plot of PFS in second or other line studies.pdf]

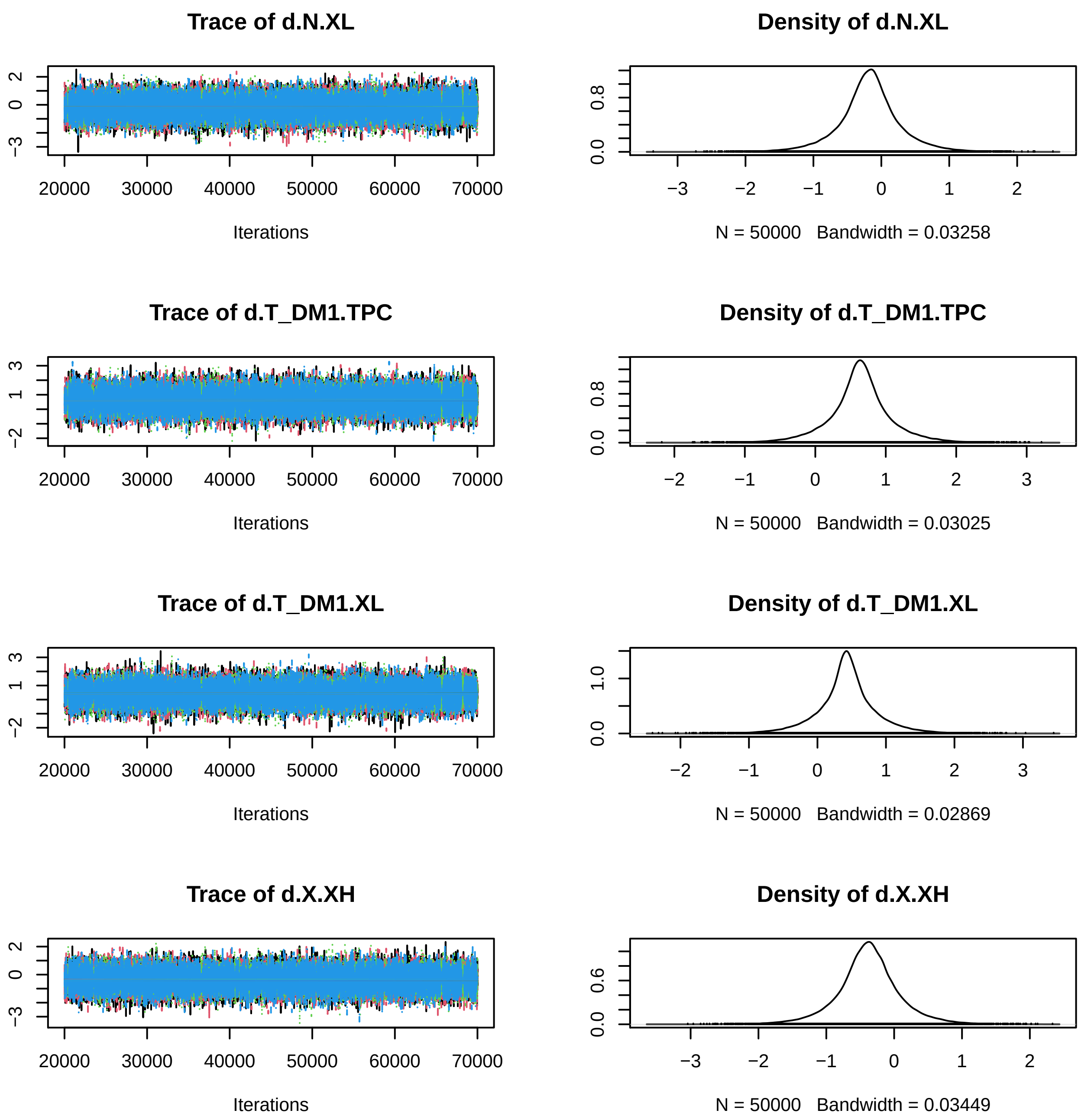
Trace of d.N.XL
Iterations
Density of d.N.XL
N = 50000 Bandwidth = 0.03258
Trace of d.T_DM1.TPC
Iterations
Density of d.T_DM1.TPC
N = 50000 Bandwidth = 0.03025
Trace of d.T_DM1.XL
Iterations
Density of d.T_DM1.XL
N = 50000 Bandwidth = 0.02869
Trace of d.X.XH
Iterations
Density of d.X.XH
N = 50000 Bandwidth = 0.03449

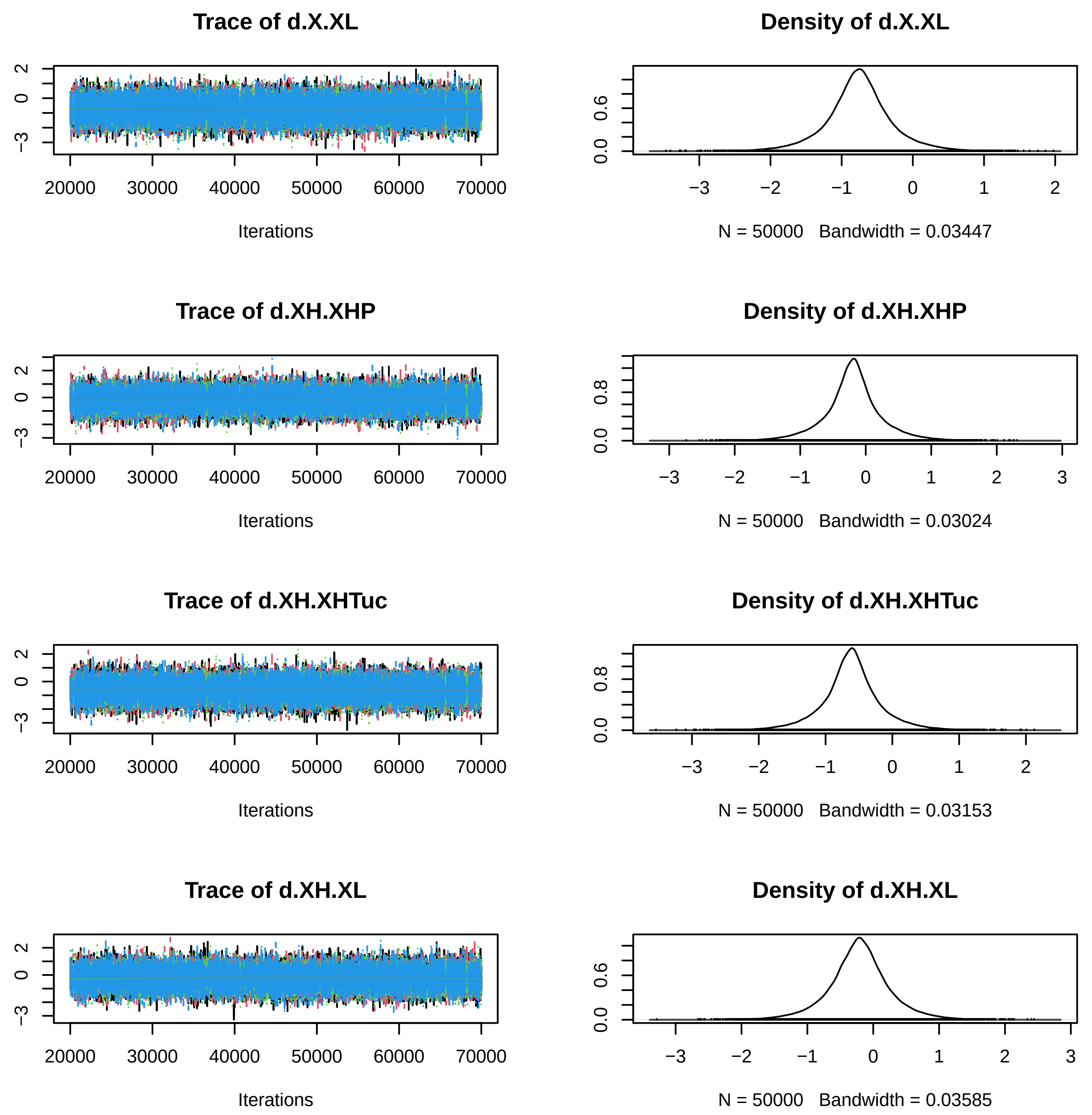
Trace of d.X.XL
Iterations
Density of d.X.XL
N = 50000 Bandwidth = 0.03447
Trace of d.XH.XHP
Iterations
Density of d.XH.XHP
N = 50000 Bandwidth = 0.03024
Trace of d.XH.XHTuc
Iterations
Density of d.XH.XHTuc
N = 50000 Bandwidth = 0.03153
Trace of d.XH.XL
Iterations
Density of d.XH.XL
N = 50000 Bandwidth = 0.03585

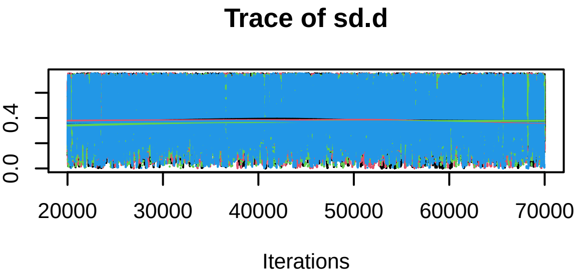
Trace of sd.d
0.0
0.4
20000
30000
40000
50000
60000
70000
Iterations

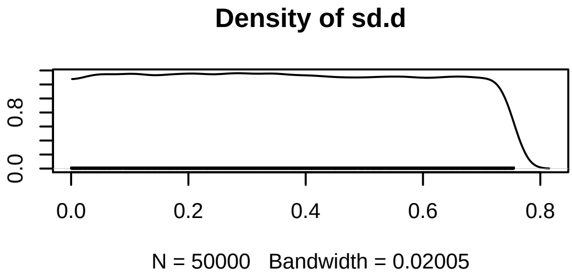
Density of sd.d
0.0
0.8
0.0
0.2
0.4
0.6
0.8
N = 50000 Bandwidth = 0.02005
