# Supplementary material for: Efficacy and Safety of Anti-HER2 Agents in Combination With Chemotherapy for Metastatic HER2-Positive Breast Cancer Patient: A Network Meta-Analysis
Source: Front Oncol. 2021 Aug 19;11:731210. doi: 10.3389/fonc.2021.731210 (PMC8416996; doi:10.3389/fonc.2021.731210)
Supplement: Supplementary file 3 [file DataSheet_3.zip › Supplementary data 18A Trace plot and density plot of ORR in second or other line studies.pdf]

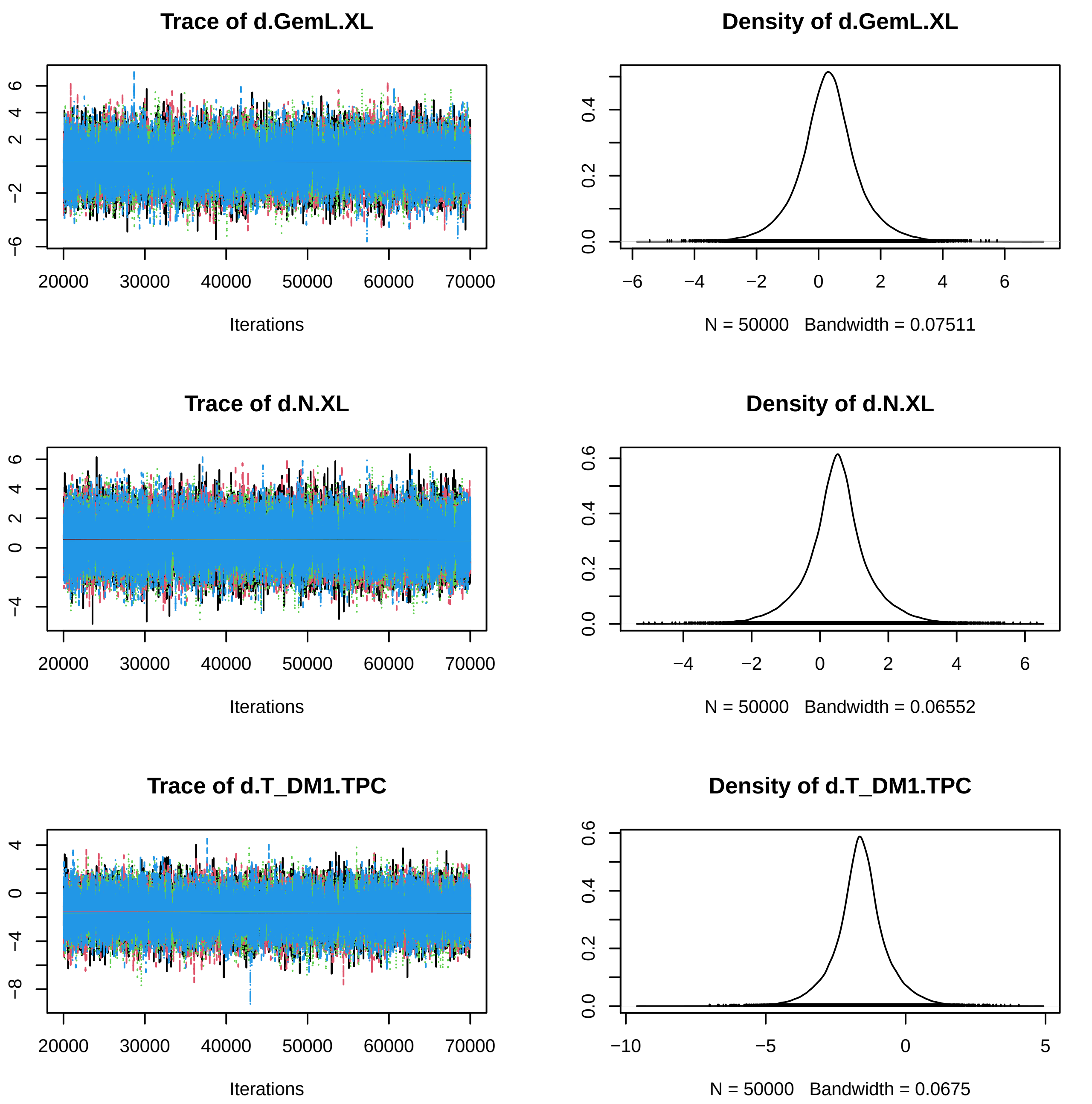
Trace of d.GemL.XL
Iterations
Density of d.GemL.XL
N = 50000   Bandwidth = 0.07511
Trace of d.N.XL
Iterations
Density of d.N.XL
N = 50000   Bandwidth = 0.06552
Trace of d.T_DM1.TPC
Iterations
Density of d.T_DM1.TPC
N = 50000   Bandwidth = 0.0675

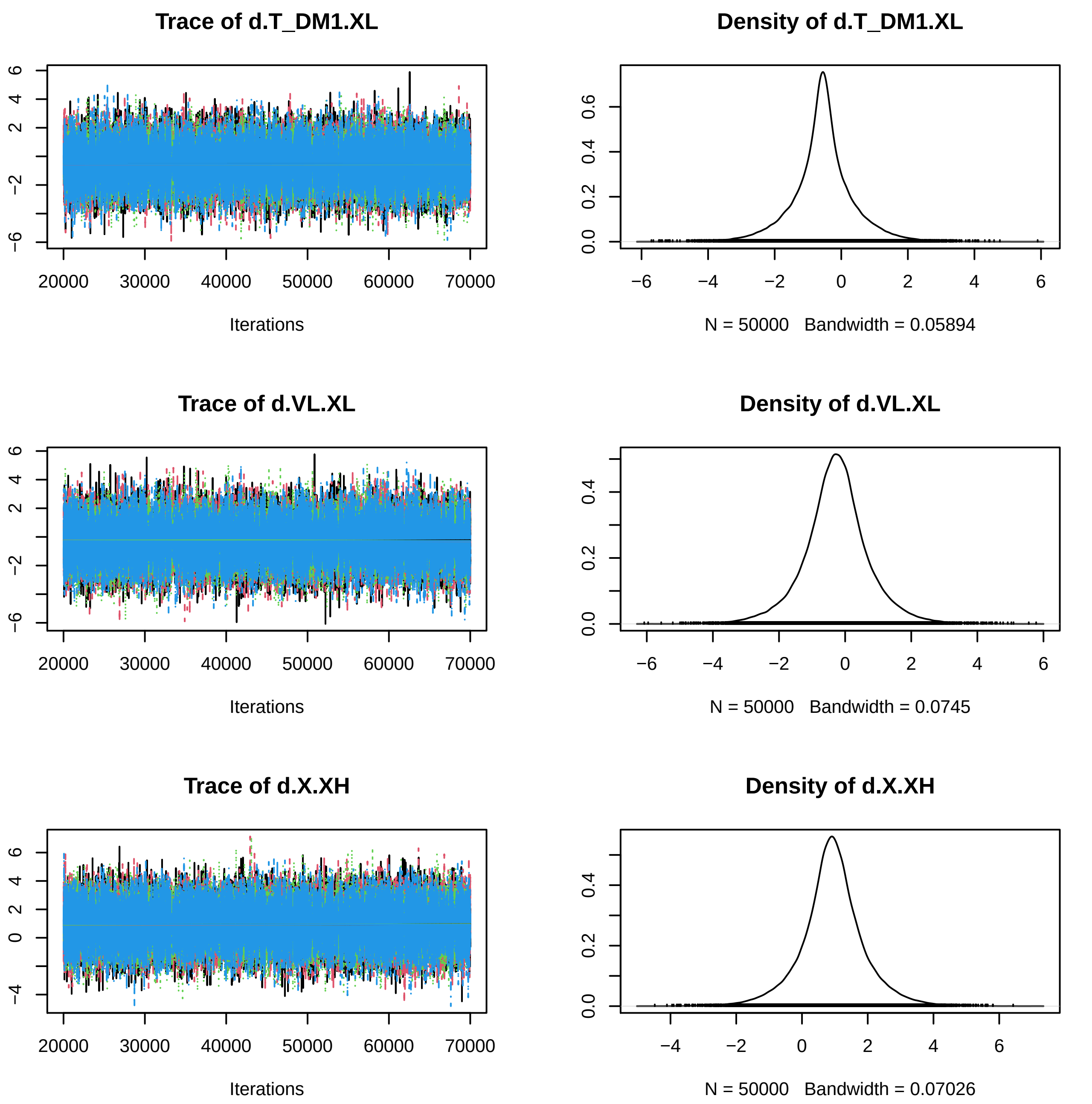

Trace of d.T_DM1.XL
Density of d.T_DM1.XL
Iterations
N = 50000   Bandwidth = 0.05894
Trace of d.VL.XL
Density of d.VL.XL
Iterations
N = 50000   Bandwidth = 0.0745
Trace of d.X.XH
Density of d.X.XH
Iterations
N = 50000   Bandwidth = 0.07026

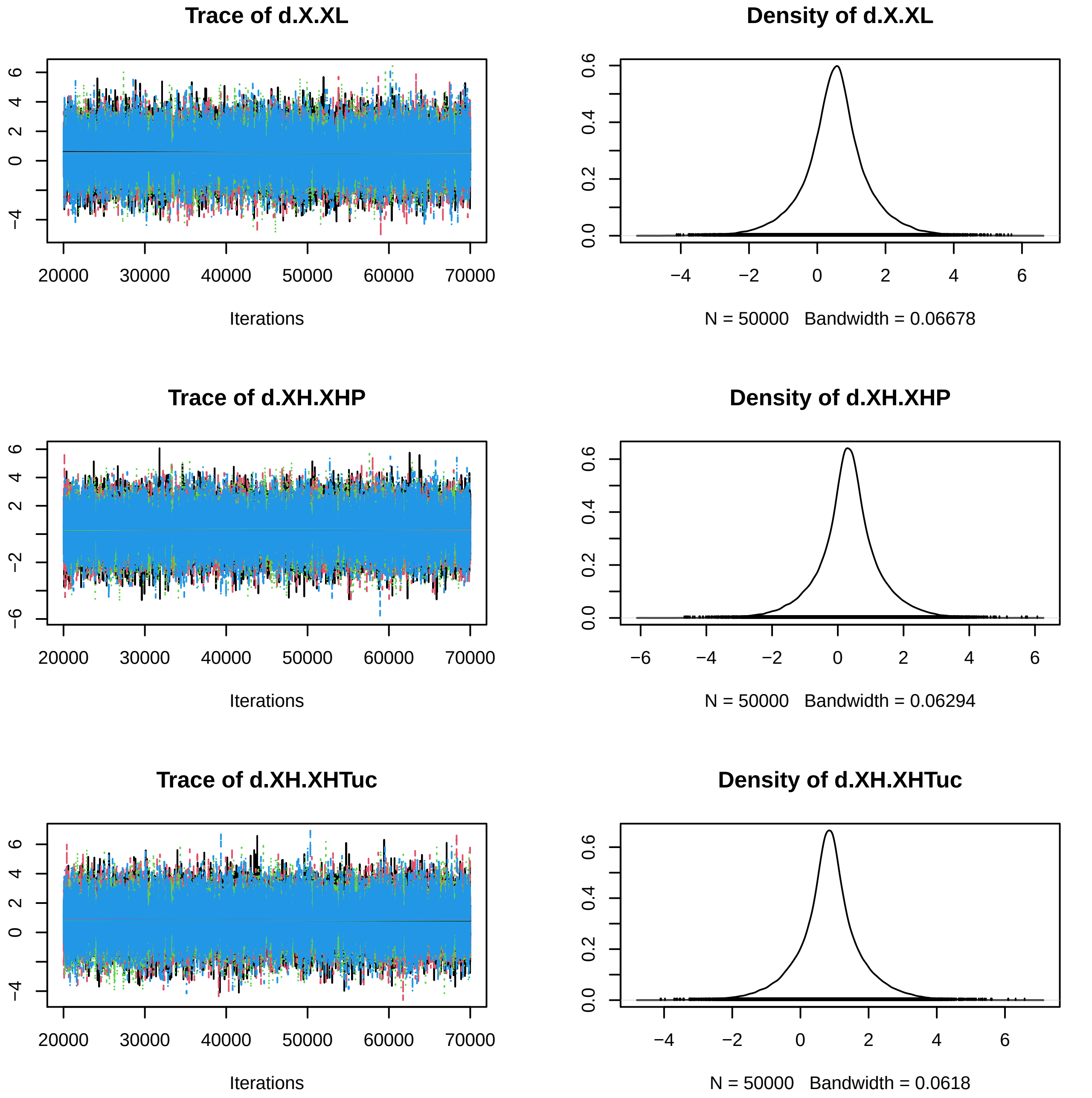

Trace of d.X.XL
Iterations
Density of d.X.XL
N = 50000 Bandwidth = 0.06678
Trace of d.XH.XHP
Iterations
Density of d.XH.XHP
N = 50000 Bandwidth = 0.06294
Trace of d.XH.XHTuc
Iterations
Density of d.XH.XHTuc
N = 50000 Bandwidth = 0.0618

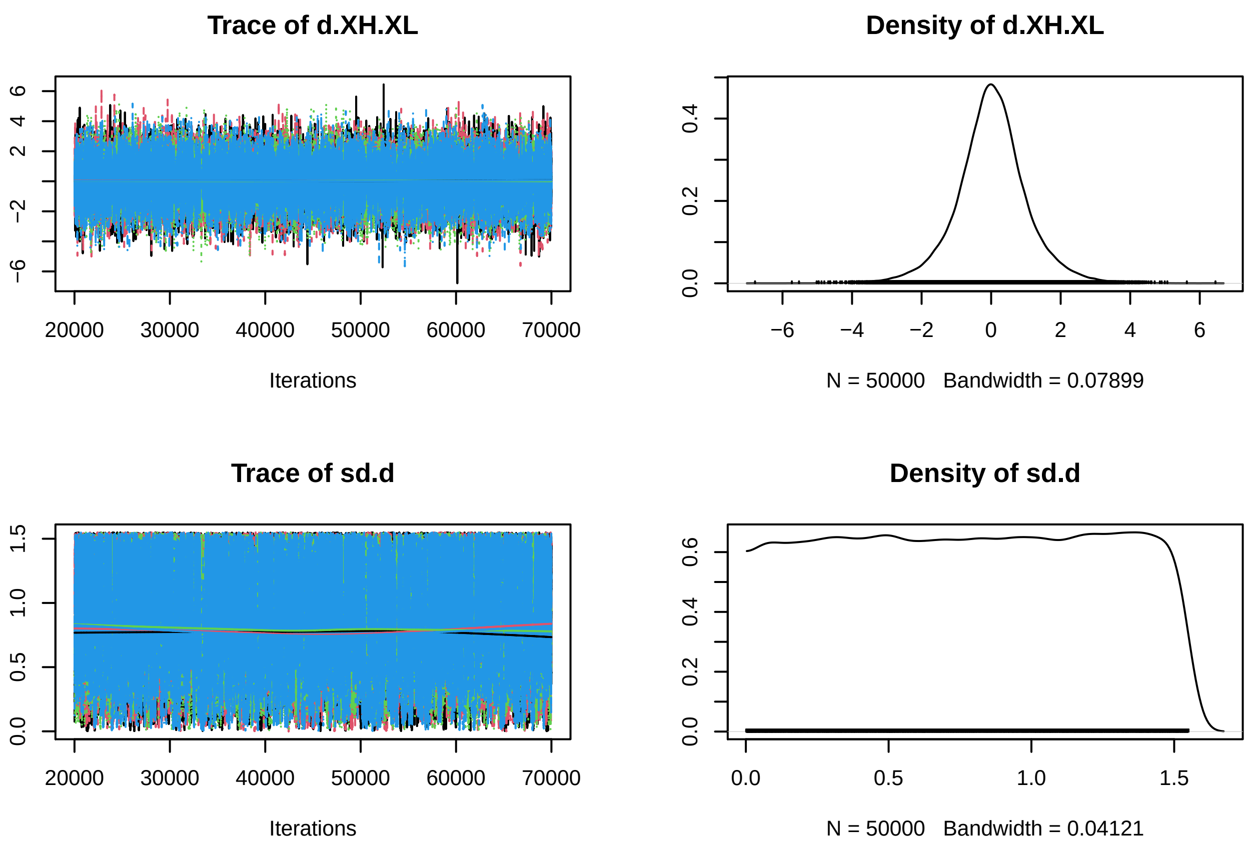
Trace of d.XH.XL
Iterations
Density of d.XH.XL
N = 50000 Bandwidth = 0.07899
Trace of sd.d
Iterations
Density of sd.d
N = 50000 Bandwidth = 0.04121
