# Supplementary material for: Efficacy and Safety of Anti-HER2 Agents in Combination With Chemotherapy for Metastatic HER2-Positive Breast Cancer Patient: A Network Meta-Analysis
Source: Front Oncol. 2021 Aug 19;11:731210. doi: 10.3389/fonc.2021.731210 (PMC8416996; doi:10.3389/fonc.2021.731210)
Supplement: Supplementary file 3 [file DataSheet_3.zip › Supplementary table 1 Intervention and outcome characteristics.docx]

**Supplementary table 1 Intervention and outcome characteristics**

| Study ID | Interventions | Outcome reported | PFS in HR subgroups |
| --- | --- | --- | --- |
| First Line |  |  |  |
| Andersson 2011([14](#_ENREF_14)) | Docetaxel 100 mg/m^2^/3 weeks + Trastuzumab 6 mg/kg/3 weeks vs Vinorelbine 30-35 mg/m^2^d1,8/3 weeks + Trastuzumab 6 mg/kg/3 weeks | TTP, OS, ORR, safety | HR+, HR- |
| Awada 2016([15](#_ENREF_15)) | Neratinib 240 mg/day + Paclitaxel 80 mg/m² d1,8,15/28 days vs Trastuzumab 2 mg/kg d1,8,15,22/28 days + Paclitaxel 80 mg/m² d1,8,15/28 days | PFS, OS, ORR, safety | HR+, HR- |
| Baselga 2012([16](#_ENREF_16))/Swain 2015([5](#_ENREF_5))/2020([30](#_ENREF_30)) | Trastuzumab 6 mg/kg/3 weeks + Docetaxel 75-100 mg/m^2^/3 weeks + Placebo420 mg/3 weeks vs Trastuzumab 6mg/kg/3 weeks + Docetaxel 75-100 mg/m^2^/3 weeks + Pertuzumab 420 mg/3 weeks | PFS, OS, ORR, safety | HR+, HR- |
| Baselga 2014([8](#_ENREF_8)) | NPLD 50 mg/m^2^/3 weeks *6 cycles + Trastuzumab 2 mg/kg/week + Paclitaxel 80 mg/m^2^/week vs Trastuzumab 2 mg/kg/week + Paclitaxel 80 mg/m^2^/week | PFS, OS, ORR, safety | HR+, HR- |
| Burstein 2007([17](#_ENREF_17)) | Trastuzumab 2 mg/kg/week + Vinorelbine 25 mg/m^2^/week vs Trastuzumab 2 mg/kg/week + Paclitaxel 80 mg/m^2^/week or Docetaxel 35 mg/m^2^ w1,2,3,5,6,7/8 weeks | TTP (no HR results), ORR, safety | NR |
| Gasparini 2007([18](#_ENREF_18)) | Trastuzumab 2 mg/kg/week + Paclitaxel 80 mg/m^2^/week vs Paclitaxel 80 mg/m^2^/week | TTP (HR result extracted from figure), ORR, safety | HR+, HR- (OR result was reported, which should be HR result according to the methodology description.) |
| Gianni 2013([20](#_ENREF_20)) | Docetaxel 100 mg/m^2^/3 weeks + Trastuzumab 6 mg/kg/3 weeks + Bevacizumab 15 mg/kg/3 weeks vs Docetaxel 100 mg/m^2^/3 weeks + Trastuzumab 6 mg/kg/3 weeks | PFS, OS, ORR, safety | HR+, HR- |
| Guan 2013([21](#_ENREF_21)) | Paclitaxel 80 mg/m^2^ d1,8,15/28 days + Lapatinib 1500 mg/day vs Paclitaxel 80 mg/m^2^ d1,8,15/28 days + Placebo | PFS, OS, ORR, safety | NR |
| Hamberg 2011([22](#_ENREF_22)) | Docetaxel 100mg/m^2^/3 weeks ×6 cycles+ Trastuzumab 2 mg/kg/week vs sequential Trastuzumab 2 mg/kg/week then Docetaxel 100mg/m^2^/3 weeks ×6cycles if progressed | PFS (data from PFScomb* vs PFStras** used in meta-analysis), OS, ORR, safety | NR |
| Hurvitz 2013([23](#_ENREF_23)) | T-DM1 3.6 mg/kg/3 weeks vs Trastuzumab 6 mg/kg/3 weeks + Docetaxel 75-100 mg/m^2^/3 weeks | PFS, OS, ORR, safety | NR |
| Hurvitz 2015([24](#_ENREF_24)) | Everolimus 10 mg/day + Paclitaxel 80mg/m^2^ d1,8,15/28 days + Trastuzumab 2mg/kg/week vs Placebo + Paclitaxel 80mg/m^2^ d1,8,15/28 days + Trastuzumab 2mg/kg/week | PFS, OS (HR result extracted from figure), ORR, safety | HR+, HR- |
| Marty 2005([31](#_ENREF_31)) | Docetaxel 100 mg/m^2^/3 weeks *6 cycles + Trastuzumab 2 mg/kg/week vs Docetaxel 100 mg/m^2^/3 weeks *6 cycles | TTP (no HR results), OS, ORR, safety | NR |
| Perez 2017([33](#_ENREF_33))/2019([32](#_ENREF_32)) | Trastuzumab 6 mg/kg/3 weeks (2mg/kg/3 weeks) + Docetaxel 75-100 mg/m^2^/3 weeks or Paclitaxel 80 mg/m^2^/week vs Placebo + T-DM1 3.6 mg/kg/3 weeks vs Pertuzumab 420 mg/3 weeks + T-DM1 3.6 mg/kg/3 weeks | PFS, OS, ORR, safety | HR+, HR- |
| Robert 2006([34](#_ENREF_34)) | [Trastuzumab 2 mg/kg/week + Paclitaxel 175 mg/m^2^ + Carboplatin (area under the time-concentration curve AUC=6 mg/mL/min)] 21day-cycles *6 vs (Trastuzumab 2 mg/kg/week + Paclitaxel 175 mg/m^2^) 21day-cycles *6 | PFS, OS, ORR, safety | NR |
| Valero 2011([35](#_ENREF_35)) | Trastuzumab 2 mg/kg d1,8,15/3 weeks + Docetaxel 100 mg/m^2^/3 weeks vs Trastuzumab 2 mg/kg d1,8,15/3 weeks + Docetaxel 75 mg/m^2^/3 weeks + Carboplatin (area under the serum concentration-time curve 6 mg/mL/min)/3 weeks | PFS, OS, ORR, safety | NR |
| Wardley 2010([36](#_ENREF_36)) | Trastuzumab 6 mg/kg/3 weeks + Docetaxel 75 mg/m^2^/3 weeks + Capecitabine 1900 mg/m^2^d1-14/21 days vs Trastuzumab 6 mg/kg/3 weeks + Docetaxel 100 mg/m^2^/3 weeks | PFS, ORR, safety | HR+, HR- (Data not available due to no 95% CI for HR subgroups) |
| Second or other Line | | | |
| Geyer 2006([19](#_ENREF_19)) | Lapatinib 1250 mg/day + Capecitabine 2000 mg/m^2^ d1-14/21 days vs Capecitabine 2500 mg/m^2^ d1-14/21 days | PFS, OS, ORR, safety | NR |
| Gómez 2016([7](#_ENREF_7)) | Lapatinib 1250 mg/day + Capecitabine 2000 mg/m^2^ d1-14/21 days vs Lapatinib 1250 mg/day + Vinorelbine 25 mg/m^2^ d1,8/21 days vs Lapatinib 1250 mg/day + Gemcitabine 1000 mg/m^2^ d1,8/21 days | PRS (no HR result), OS (no HR results), ORR, safety | NR |
| Krop 2014([25](#_ENREF_25))/2017([26](#_ENREF_26)) | physician’s choice (chemotherapy, hormonal therapy, or HER2-directed therapy) vs T-DM1 3.6 mg/kg/3 weeks | PFS, OS, ORR, safety | HR+, HR- |
| Lin 2011([37](#_ENREF_37)) | Lapatinib 1250 mg/day + Capecitabine 2000 mg/m^2^ d1-14/21 days vs Lapatinib 1250 mg/day + Topotecan 3.2 mg/m^2^ d1,8,15/28 days | CNS ORR (not used in meta-analysis), safety | NR |
| Martin 2013([38](#_ENREF_38)) | Neratinib 240 mg/day vs Lapatinib 1250 mg/day + Capecitabine 2000 mg/m^2^ d1-14/21 days | PFS, OS, ORR, safety | NR |
| Murthy 2020([39](#_ENREF_39))/Lin 2020([27](#_ENREF_27)) | Trastuzumab 6mg/kg/ 21days+ Capecitabine 1000mg/m^2^ Bid d1-14/ 21days + Tucatinib 300 mg Bid vs Trastuzumab 6mg/kg/ 21days+ Capecitabine 1000mg/m^2^ Bid d1-14/ 21days + Placebo | PFS (data form first 480 patients was used in meta-analysis), OS, ORR, safety | HR+, HR- |
| Takano 2018([40](#_ENREF_40)) | Trastuzumab 2 mg/kg/week + Capecitabine 2500 mg/m^2^ d1-14/21 days vs Lapatinib 1250 mg/day + Capecitabine 2000 mg/m^2^ d1-14/21 days | PFS, OS, ORR, safety | HR+, HR- |
| Urruticoechea 2017([41](#_ENREF_41)) | Trastuzumab 6 mg/kg/3 weeks + Capecitabine 2500 mg/m^2^ d1-14/21 days vs Pertuzumab 420 mg/3 weeks + Trastuzumab 6 mg/kg/3 weeks + Capecitabine 2000 mg/m^2^ d1-14/21 days | PFS, OS, ORR, safety | HR+, HR- |
| Verma 2012([42](#_ENREF_42))/Diéras 2017([6](#_ENREF_6)) | Lapatinib 1250 mg/day + Capecitabine 2000 mg/m^2^ d1-14/21 days vs T-DM1 3.6 mg/kg/3 weeks | PFS, OS, ORR, safety | HR+, HR- |
| von Minckwitz 2009([43](#_ENREF_43))/2011([44](#_ENREF_44)) | Capecitabine 2500 mg/m^2^ d1-14/21 days + Trastuzumab 6 mg/kg/3 weeks vs Capecitabine 2500 mg/m^2^ d1-14/21 days | TTP, OS, ORR, safety | NR |
| Third or other Line | | | |
| Tolaney 2020([29](#_ENREF_29)) | Abemaciclib 150 mg/12 hours/3 weeks + Trastuzumab 6 mg/kg/3 weeks + Fulvestrant 500 mg/4 weeks vs Abemaciclib 150 mg/ 12 hours/ 3 weeks + Trastuzumab 6 mg/kg/3 weeks vs Standard-of-care chemotherapy + Trastuzumab 6 mg/kg/3 weeks | PFS, ORR, safety | HR+, HR- |

*: PFScomb: PFS in the TH group was defined as time from start of treatment to the data of documented disease progression, initiation of another systemic anticancer treatment without documented objective (radiologic) tumor progression, or death, whichever occurred first.

**: PFStras: PFS in the H→Tgroup,by using the same criteria as already mentioned.
