# Supplementary material for: Efficacy and Safety of Anti-HER2 Agents in Combination With Chemotherapy for Metastatic HER2-Positive Breast Cancer Patient: A Network Meta-Analysis
Source: Front Oncol. 2021 Aug 19;11:731210. doi: 10.3389/fonc.2021.731210 (PMC8416996; doi:10.3389/fonc.2021.731210)
Supplement: Supplementary file 3 [file DataSheet_3.zip › Supplementary table 2 Risk of bias.docx]

**Supplementary table 2 Risk of bias**

| Study ID | RANDOMISATION | ALLOCATION CONCEALMENT | BLINDING OF PARTICIPANTS AND INVESTIGATORS | BLINDING OF OUTCOME ASSESSMENT | Selective report of outcomes | STUDY ATTRITION | OTHER |
| --- | --- | --- | --- | --- | --- | --- | --- |
| Andersson 2011([14](#_ENREF_14)) | Low | Low | Moderate | Low | Low | Low | Moderate |
| Awada 2016([15](#_ENREF_15)) | Low | Low | High | Low | Low | Low | Moderate |
| Burstein 2007([17](#_ENREF_17)) | Low | Moderate | Moderate | Low | Low | Moderate | Low |
| Baselga 2012([16](#_ENREF_16))/Swain 2015([5](#_ENREF_5))/2020([30](#_ENREF_30)) | Low | Low | Low | Low | Low | Low | Moderate |
| Krop 2014([25](#_ENREF_25))/2017([26](#_ENREF_26)) | Low | Moderate | High | High | Low | Low | Moderate |
| Lin 2011([37](#_ENREF_37)) | Moderate | Moderate | High | High | Moderate | Low | High |
| Valero 2011([35](#_ENREF_35)) | Moderate | Moderate | High | High | Low | Low | Moderate |
| Verma 2012([42](#_ENREF_42))/Diéras 2017([6](#_ENREF_6)) | Low | Low | High | Low | Low | Low | Moderate |
| Gasparini 2007([18](#_ENREF_18)) | Low | Low | High | Low | Low | Low | Moderate |
| Gianni 2013([20](#_ENREF_20)) | Low | Moderate | High | Low | Low | Low | Moderate |
| Guan 2013([21](#_ENREF_21)) | Low | Low | Low | Low | Low | Low | Moderate |
| Takano 2018([40](#_ENREF_40)) | Moderate | High | Moderate | Moderate | Moderate | Low | High |
| Murthy 2020([39](#_ENREF_39))/Lin 2020([26](#_ENREF_26)) | Low | Moderate | Low | Low | Low | Low | High |
| von Minckwitz 2009/2011([44](#_ENREF_44)) | Moderate | High | High | High | Low | Low | High |
| Tolaney 2020([29](#_ENREF_29)) | Low | Low | High | Moderate | Low | Low | Low |
| Urruticoechea 2017([41](#_ENREF_41)) | Low | Low | High | Moderate | Low | Low | Low |
| Martin 2013([38](#_ENREF_38)) | Low | Moderate | High | Moderate | Low | Low | Low |
| Baselga 2014([8](#_ENREF_8)) | Moderate | Moderate | High | Low | Low | Low | Moderate |
| Geyer 2006([19](#_ENREF_19)) | Low | Low | High | Low | Low | Low | Moderate |
| Gómez 2016([7](#_ENREF_7)) | Low | Moderate | High | Low | Low | Low | Moderate |
| Hamberg 2011([22](#_ENREF_22)) | Low | Low | Moderate | Moderate | Low | High | Moderate |
| Hurvitz 2013([23](#_ENREF_23)) | Low | Moderate | High | Low | Low | Low | Moderate |
| Hurvitz 2015([24](#_ENREF_24)) | Low | Low | Low | Low | Low | Low | Moderate |
| Marty 2005([31](#_ENREF_31)) | Moderate | Low | Low | Low | Low | Low | Low |
| Perez 2017([33](#_ENREF_33))/2019([33](#_ENREF_33)) | Low | Low | Low | Low | Low | Low | Moderate |
| Robert 2006([34](#_ENREF_34)) | Low | Moderate | Moderate | Low | Low | Low | Moderate |
| Wardley 2010([36](#_ENREF_36)) | Moderate | Moderate | High | Low | Low | Low | Moderate |
