# Supplementary material for: Efficacy and Safety of Anti-HER2 Agents in Combination With Chemotherapy for Metastatic HER2-Positive Breast Cancer Patient: A Network Meta-Analysis
Source: Front Oncol. 2021 Aug 19;11:731210. doi: 10.3389/fonc.2021.731210 (PMC8416996; doi:10.3389/fonc.2021.731210)
Supplement: Supplementary file 3 [file DataSheet_3.zip › Supplementary data 14A Trace plot and density plot of OS in second or other line studies.pdf]

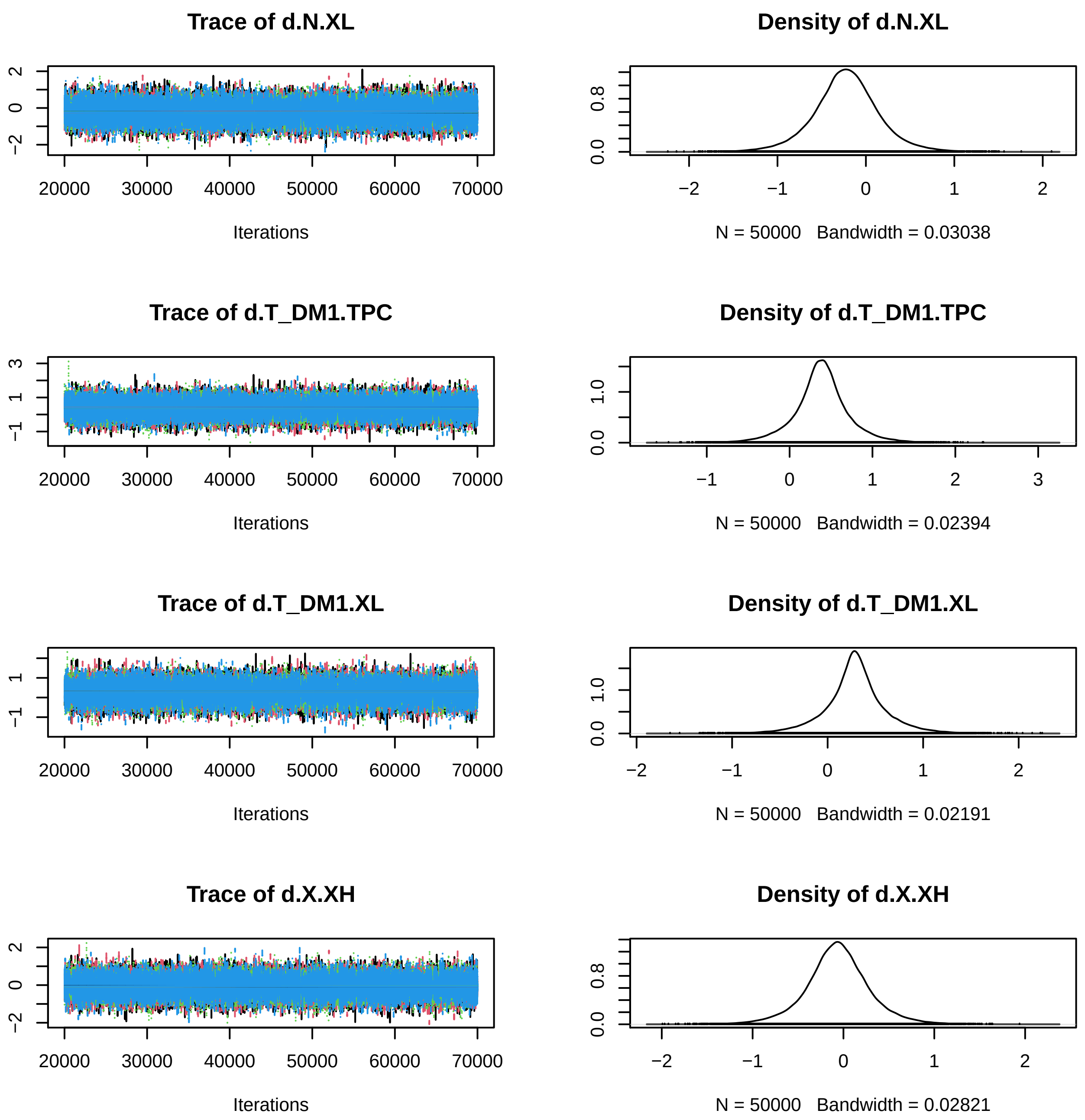
Trace of d.N.XL
Iterations
Density of d.N.XL
N = 50000 Bandwidth = 0.03038
Trace of d.T_DM1.TPC
Iterations
Density of d.T_DM1.TPC
N = 50000 Bandwidth = 0.02394
Trace of d.T_DM1.XL
Iterations
Density of d.T_DM1.XL
N = 50000 Bandwidth = 0.02191
Trace of d.X.XH
Iterations
Density of d.X.XH
N = 50000 Bandwidth = 0.02821

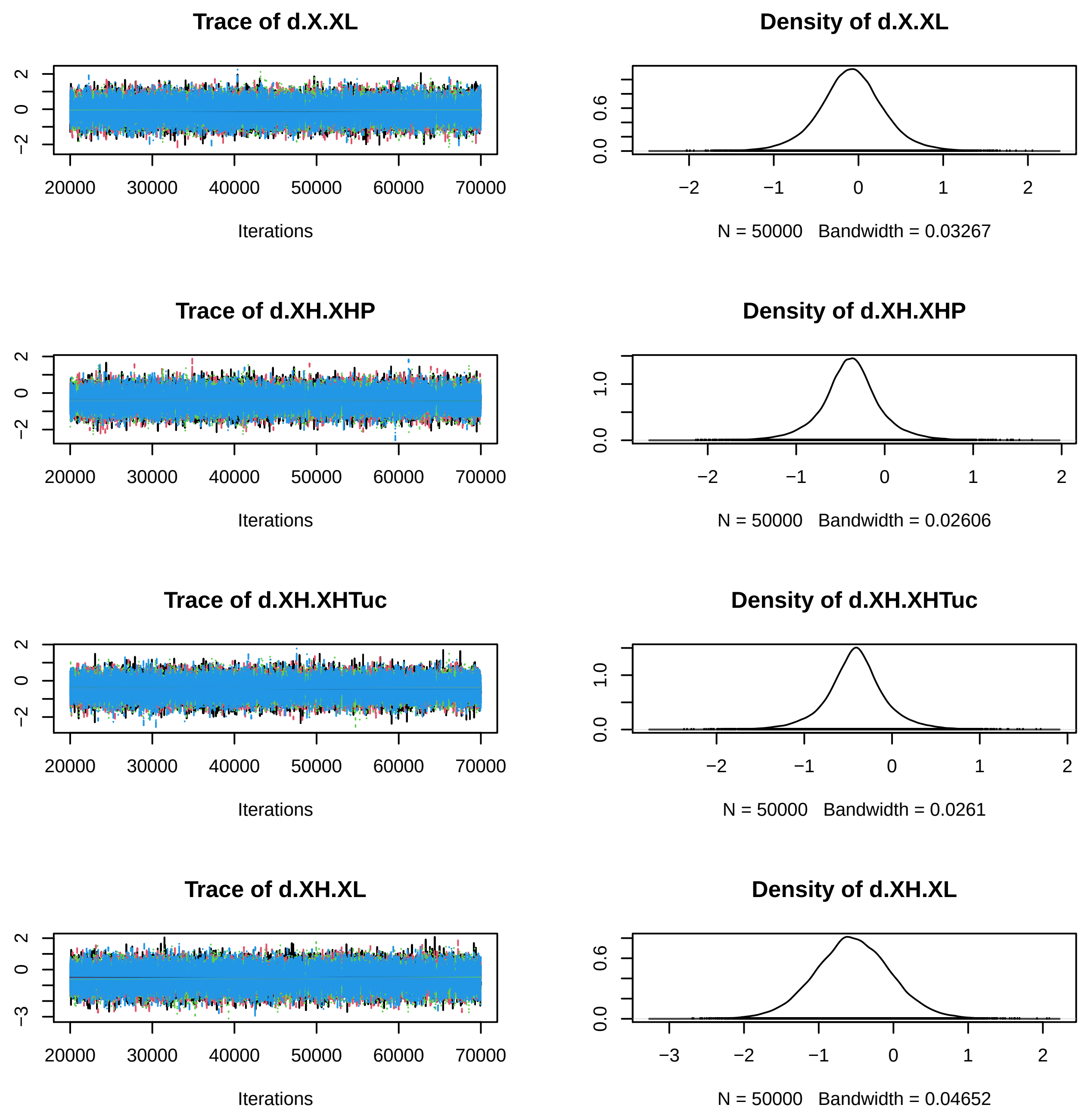

Trace of d.X.XL
Iterations
Density of d.X.XL
N = 50000 Bandwidth = 0.03267
Trace of d.XH.XHP
Iterations
Density of d.XH.XHP
N = 50000 Bandwidth = 0.02606
Trace of d.XH.XHTuc
Iterations
Density of d.XH.XHTuc
N = 50000 Bandwidth = 0.0261
Trace of d.XH.XL
Iterations
Density of d.XH.XL
N = 50000 Bandwidth = 0.04652

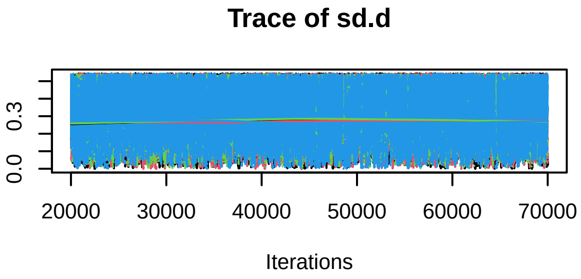

Trace of sd.d
0.0
0.3
20000
30000
40000
50000
60000
70000
Iterations

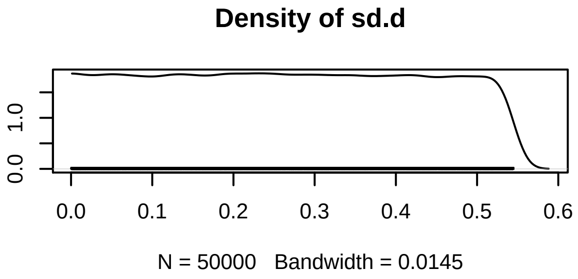

Density of sd.d
0.0
1.0
0.0
0.1
0.2
0.3
0.4
0.5
0.6
N = 50000 Bandwidth = 0.0145
