# Supplementary material for: Efficacy and Safety of Anti-HER2 Agents in Combination With Chemotherapy for Metastatic HER2-Positive Breast Cancer Patient: A Network Meta-Analysis
Source: Front Oncol. 2021 Aug 19;11:731210. doi: 10.3389/fonc.2021.731210 (PMC8416996; doi:10.3389/fonc.2021.731210)
Supplement: Supplementary file 3 [file DataSheet_3.zip › Supplementary data 16 Trace plot and density plot of ORR in first line studies.pdf]

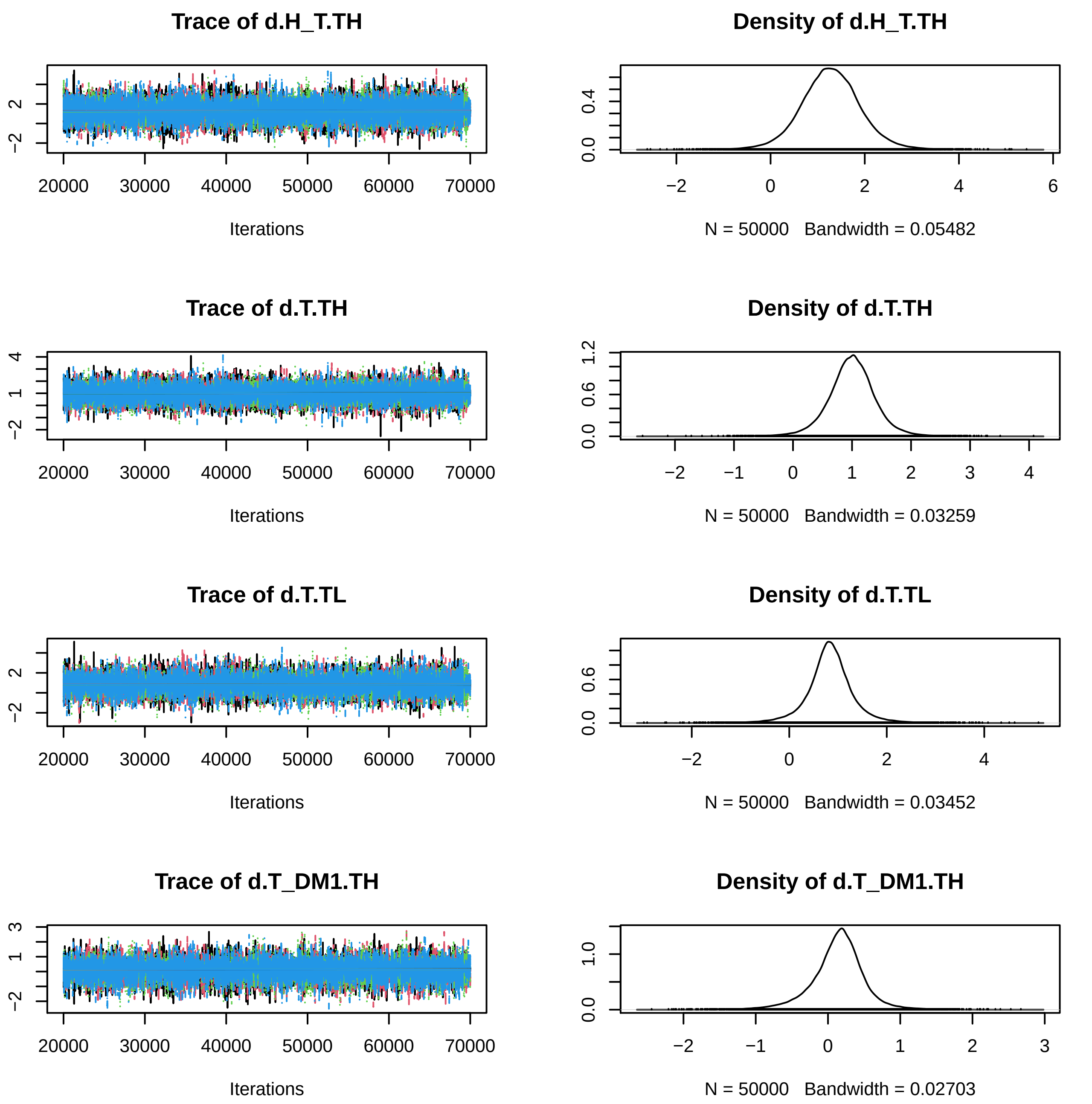

Trace of d.H_T.TH
Iterations
Density of d.H_T.TH
N = 50000 Bandwidth = 0.05482
Trace of d.T.TH
Iterations
Density of d.T.TH
N = 50000 Bandwidth = 0.03259
Trace of d.T.TL
Iterations
Density of d.T.TL
N = 50000 Bandwidth = 0.03452
Trace of d.T_DM1.TH
Iterations
Density of d.T_DM1.TH
N = 50000 Bandwidth = 0.02703

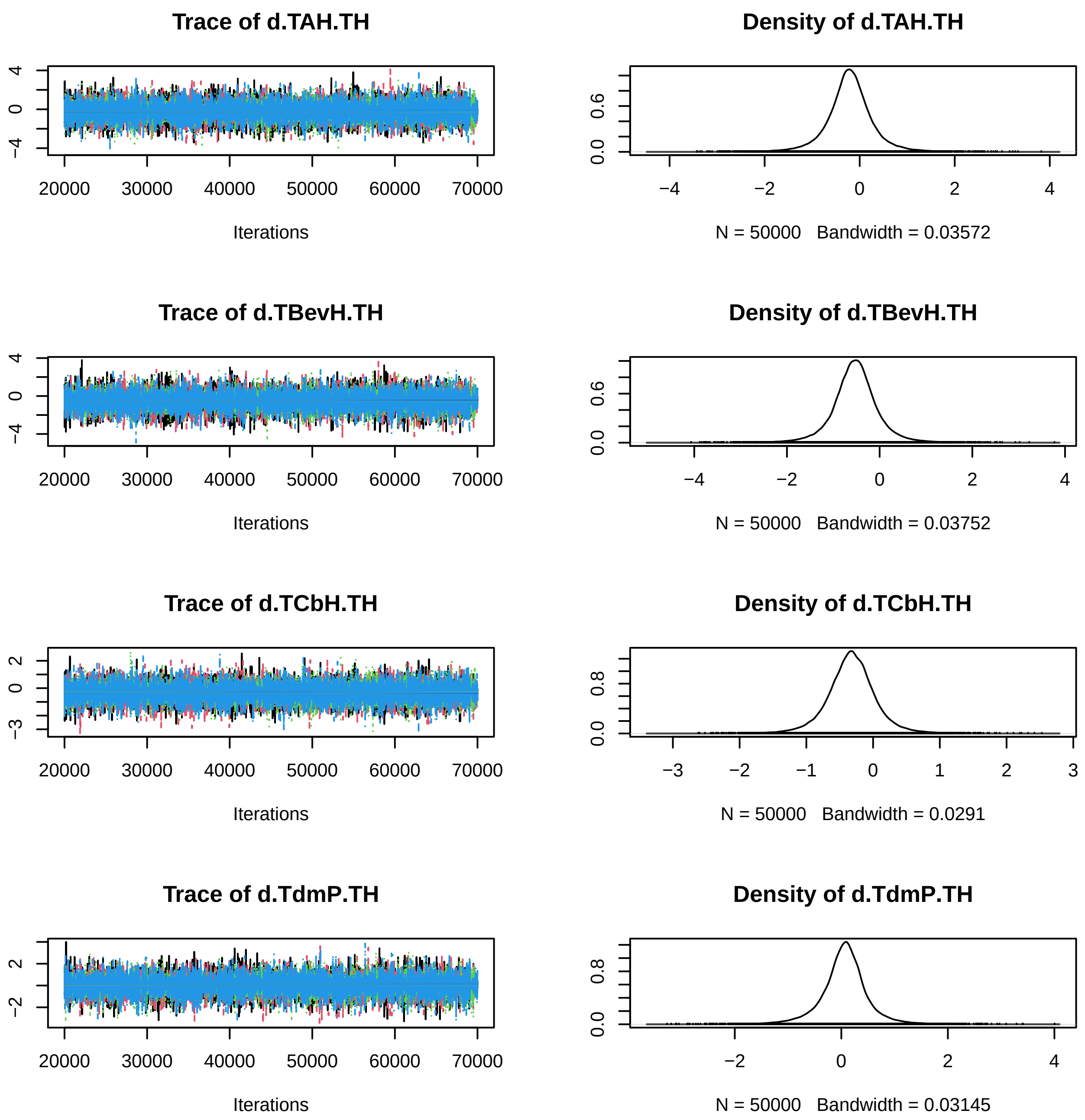
Trace of d.TAH.TH
Iterations
Density of d.TAH.TH
N = 50000 Bandwidth = 0.03572
Trace of d.TBevH.TH
Iterations
Density of d.TBevH.TH
N = 50000 Bandwidth = 0.03752
Trace of d.TCbH.TH
Iterations
Density of d.TCbH.TH
N = 50000 Bandwidth = 0.0291
Trace of d.TdmP.TH
Iterations
Density of d.TdmP.TH
N = 50000 Bandwidth = 0.03145

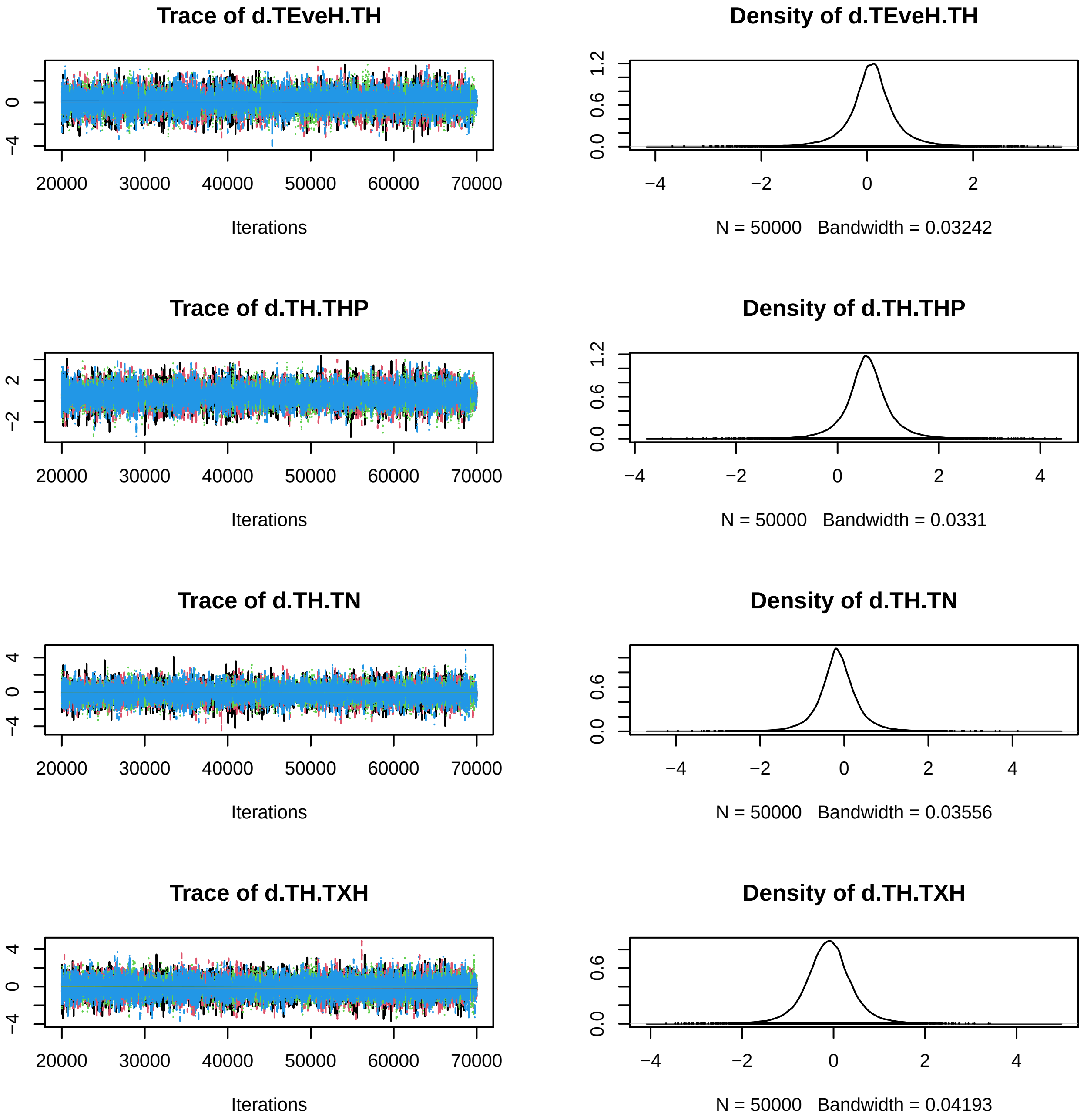

Trace of d.TEveH.TH
Iterations
Density of d.TEveH.TH
N = 50000 Bandwidth = 0.03242
Trace of d.TH.THP
Iterations
Density of d.TH.THP
N = 50000 Bandwidth = 0.0331
Trace of d.TH.TN
Iterations
Density of d.TH.TN
N = 50000 Bandwidth = 0.03556
Trace of d.TH.TXH
Iterations
Density of d.TH.TXH
N = 50000 Bandwidth = 0.04193

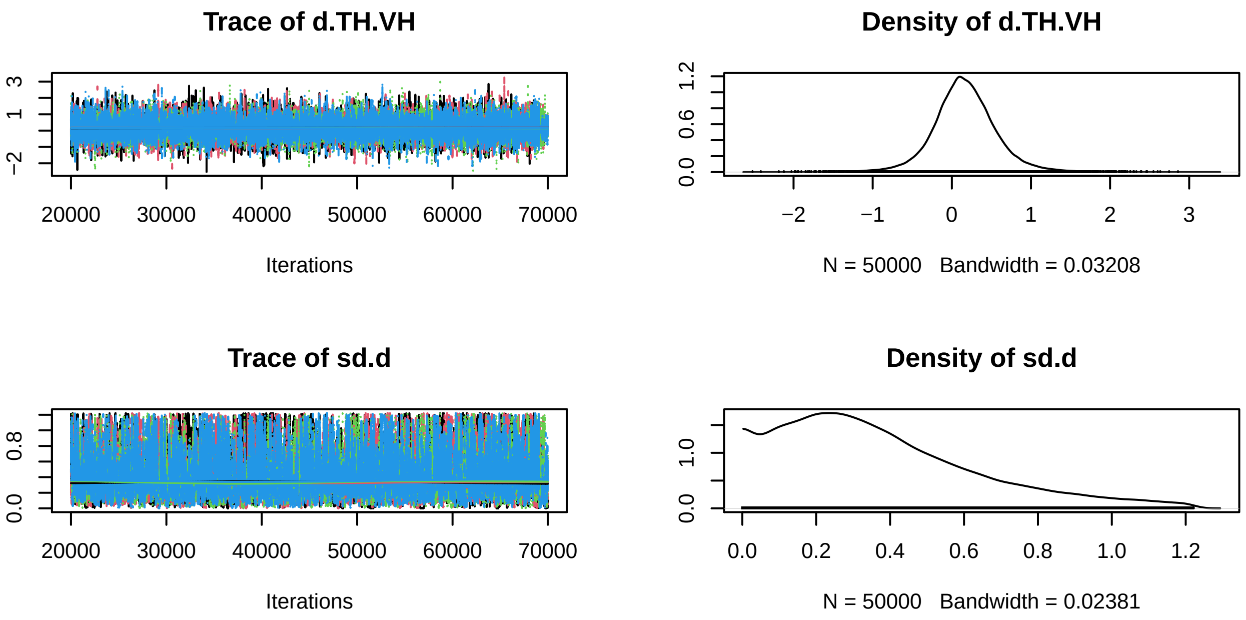
Trace of d.TH.VH
3
1
−2
20000
30000
40000
50000
60000
70000
Iterations
Density of d.TH.VH
1.2
0.6
0.0
−2
−1
0
1
2
3
N = 50000   Bandwidth = 0.03208
Trace of sd.d
0.8
0.0
20000
30000
40000
50000
60000
70000
Iterations
Density of sd.d
1.0
0.0
0.0
0.2
0.4
0.6
0.8
1.0
1.2
N = 50000   Bandwidth = 0.02381
